# Supplementary material for: New Compounds with Bioisosteric Replacement of Classic Choline Kinase Inhibitors Show Potent Antiplasmodial Activity
Source: Pharmaceutics. 2021 Nov 2;13(11):1842. doi: 10.3390/pharmaceutics13111842 (PMC8621770; doi:10.3390/pharmaceutics13111842)
Supplement: Supplementary file 1 [file pharmaceutics-13-01842-s001.zip › pharmaceutics-1417808-supplementary.pdf]

pharmaceutics

MDPI

Supplementary Materials: New Compounds with Bioisosteric Replacement of Classic Choline Kinase Inhibitors Show Potent Antiplasmodial Activity

Francisco José Aguilar-Troyano, Archimede Torretta, Gianluca Rubbini, Alberto Fasiolo, Pilar María Luque-Navarro, María Paz Carrasco-Jimenez, Guiomar Pérez-Moreno, Cristina Bosch-Navarrete, Dolores González-Pacanowska, Emilio Parisini and Luisa Carlota Lopez-Cara

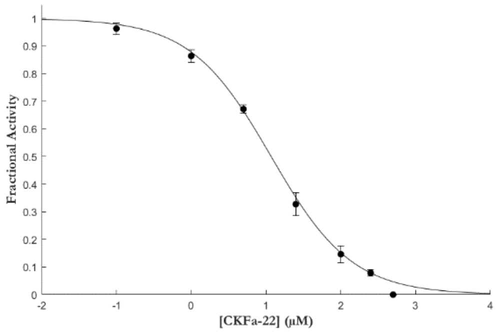

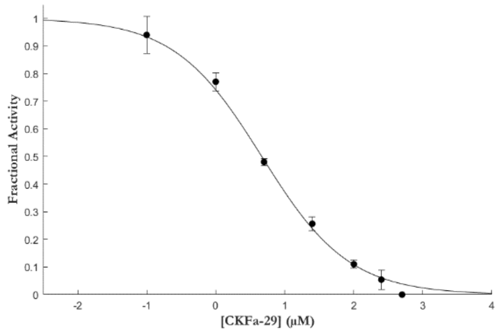

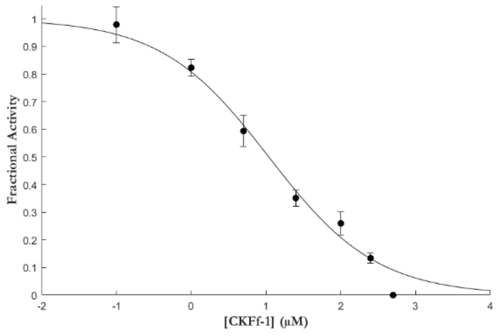

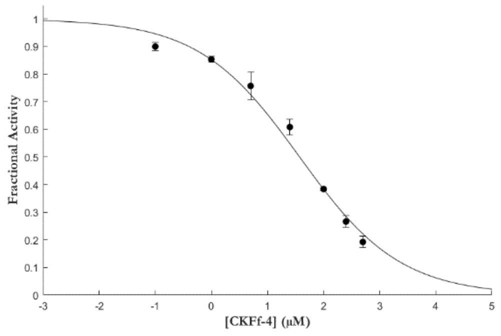

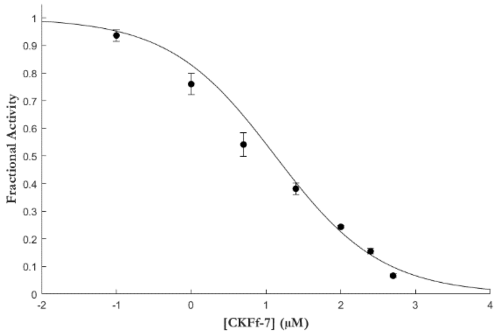

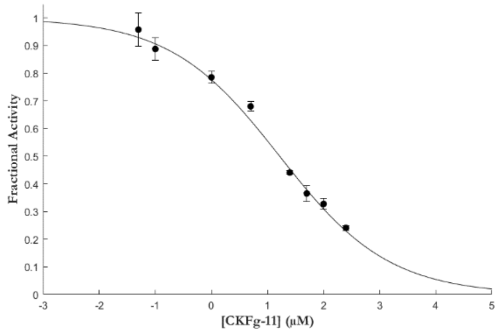

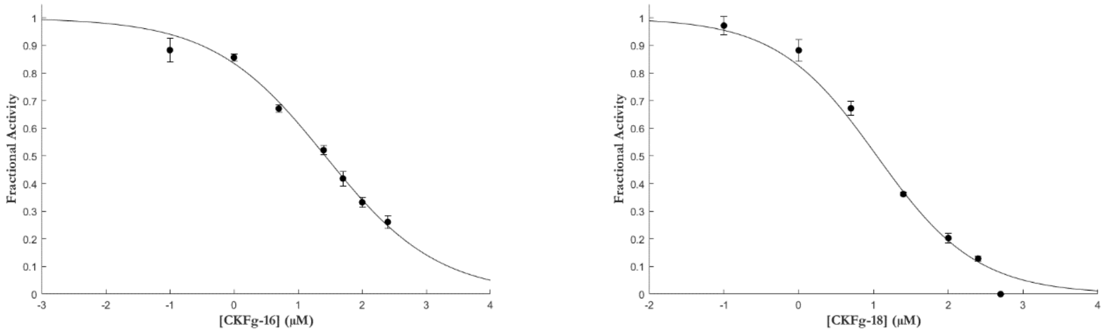

Figure S1. IC_{50} Curves Inhibition of *Pf*CK.

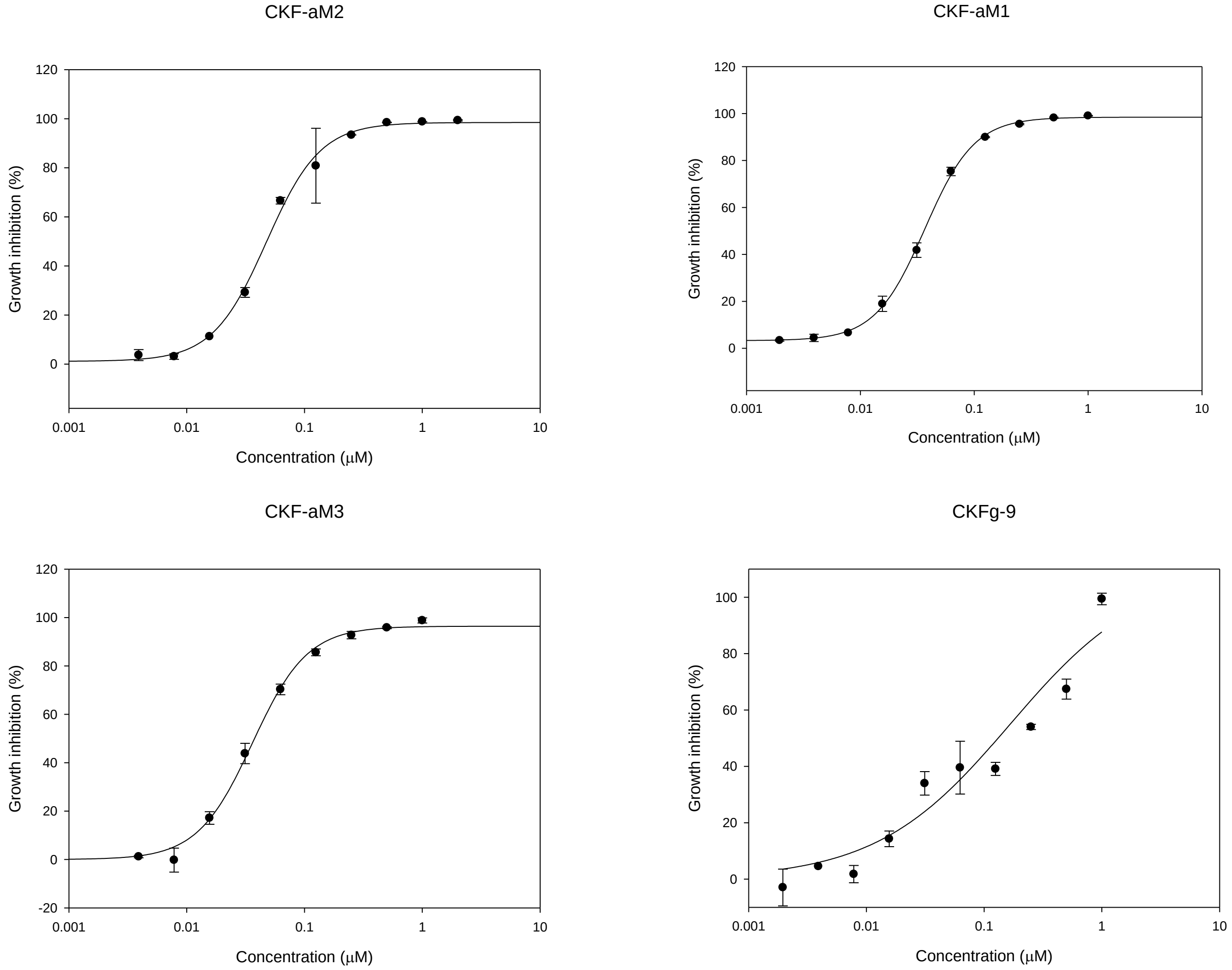

CKFa-21

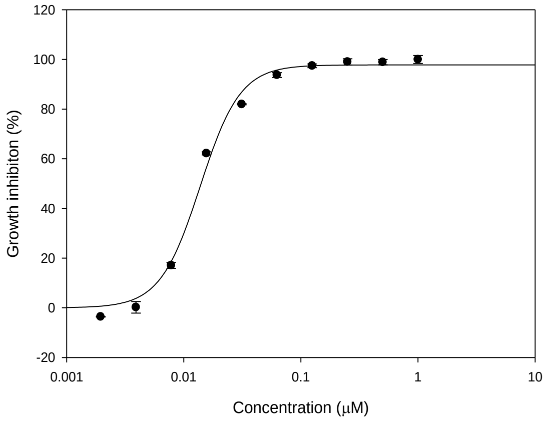

CKFg-14

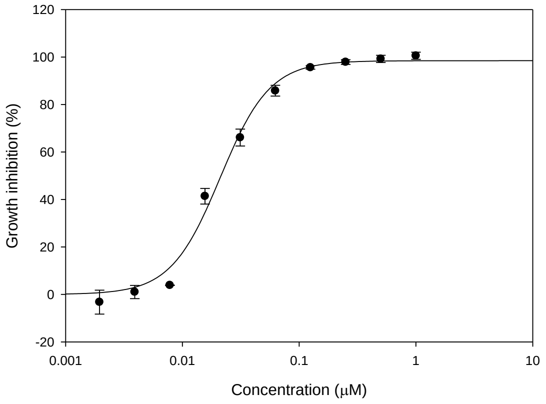

CKF-a24

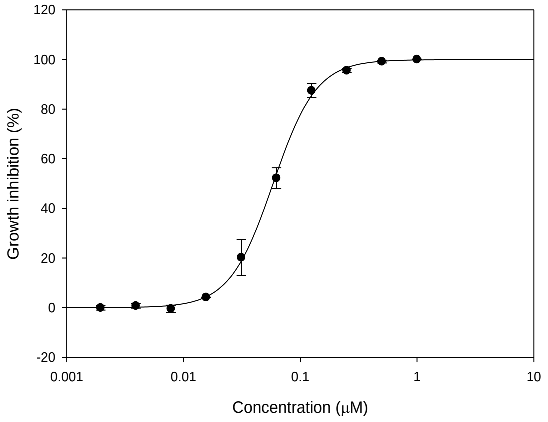

CKFg-30

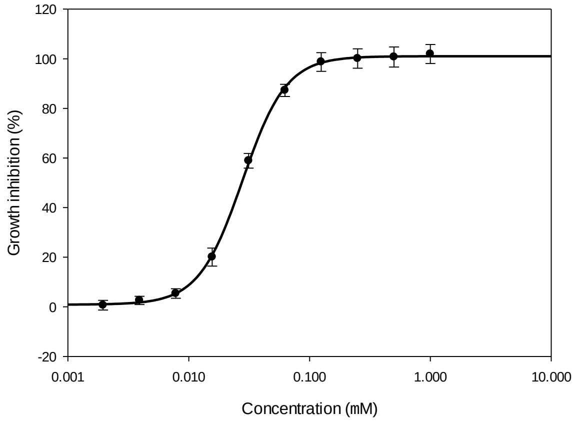

CKFg-30

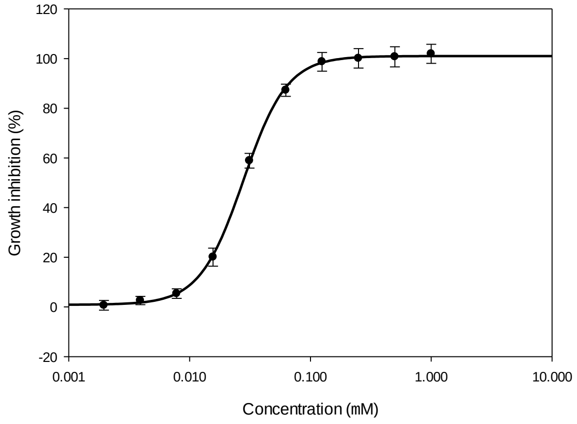

CKF-g10

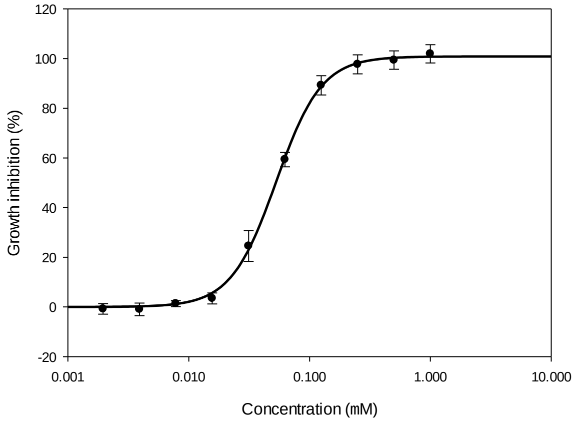

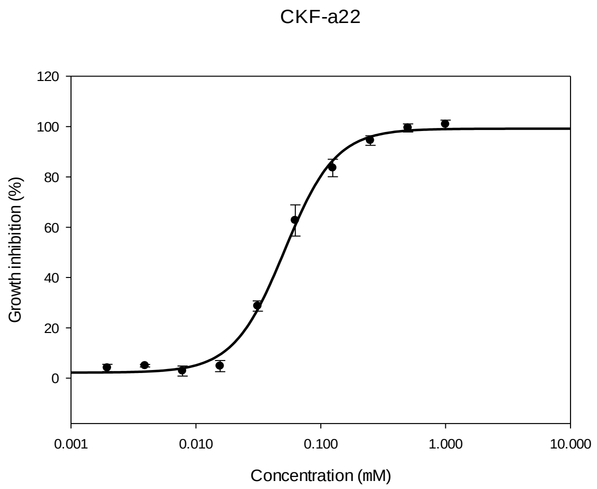
CKF-a22
Growth inhibition (%)
120
100
80
60
40
20
0
0.001
0.010
0.100
1.000
10.000
Concentration (mM)

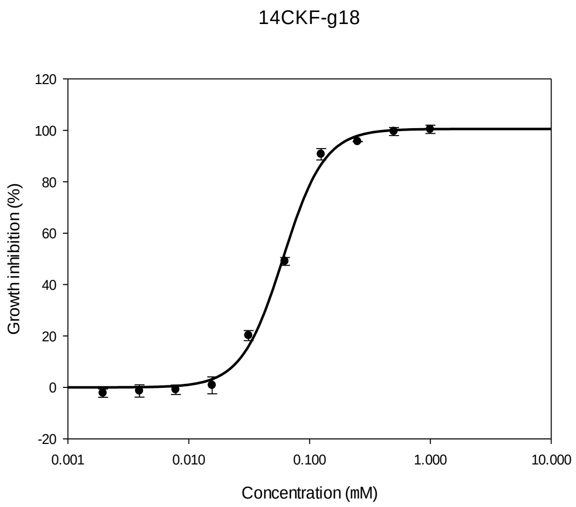
14CKF-g18
Growth inhibition (%)
120
100
80
60
40
20
0
-20
0.001
0.010
0.100
1.000
10.000
Concentration (mM)

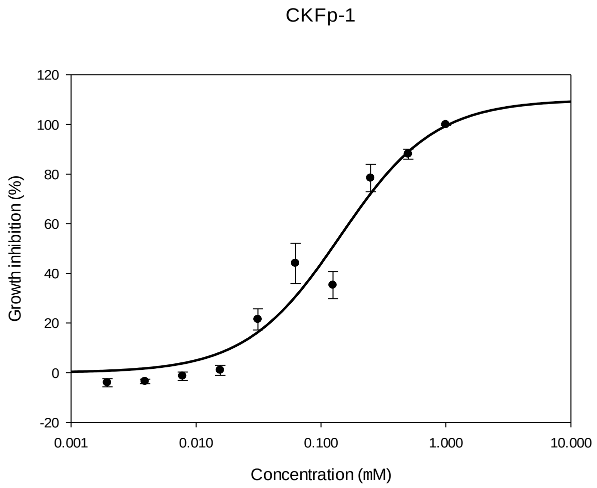
CKFp-1
Growth inhibition (%)
120
100
80
60
40
20
0
-20
0.001
0.010
0.100
1.000
10.000
Concentration (mM)

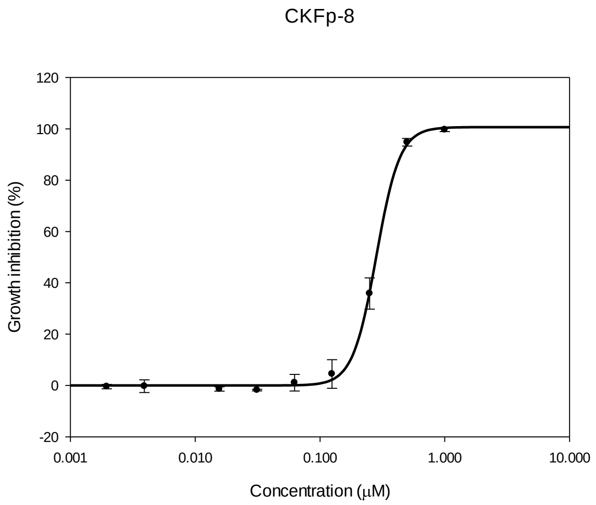
CKFp-8
Growth inhibition (%)
120
100
80
60
40
20
0
-20
0.001
0.010
0.100
1.000
10.000
Concentration (μM)

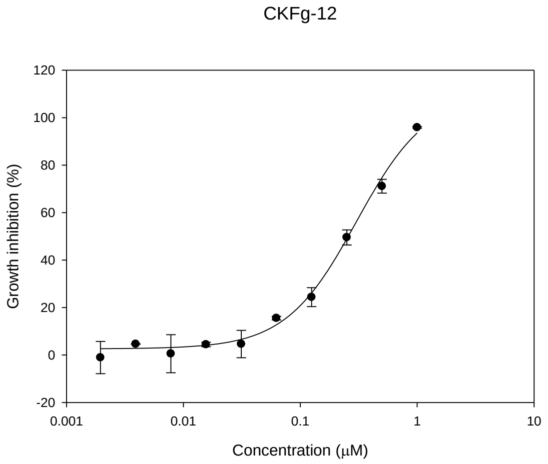
CKFg-12
Growth inhibition (%)
120
100
80
60
40
20
0
-20
0.001
0.01
0.1
1
10
Concentration (μM)

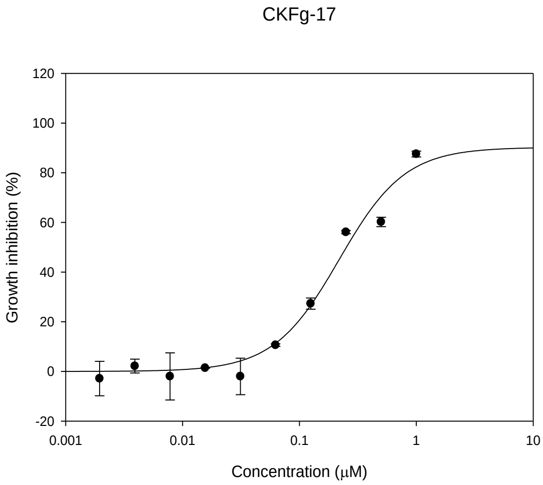
CKFg-17
Growth inhibition (%)
120
100
80
60
40
20
0
-20
0.001
0.01
0.1
1
10
Concentration (μM)

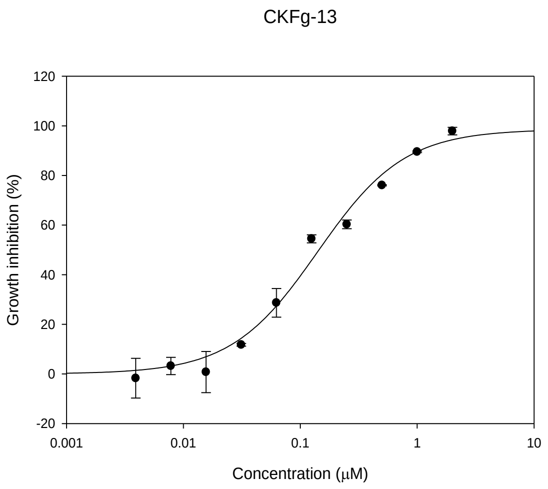
CKFg-13
Growth inhibition (%)
120
100
80
60
40
20
0
-20
0.001
0.01
0.1
1
10
Concentration (μM)

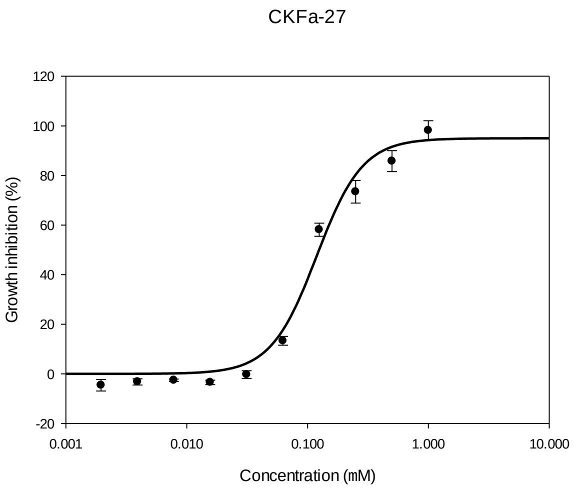
CKFa-27
Growth inhibition (%)
120
100
80
60
40
20
0
-20
0.001
0.010
0.100
1.000
10.000
Concentration (mM)

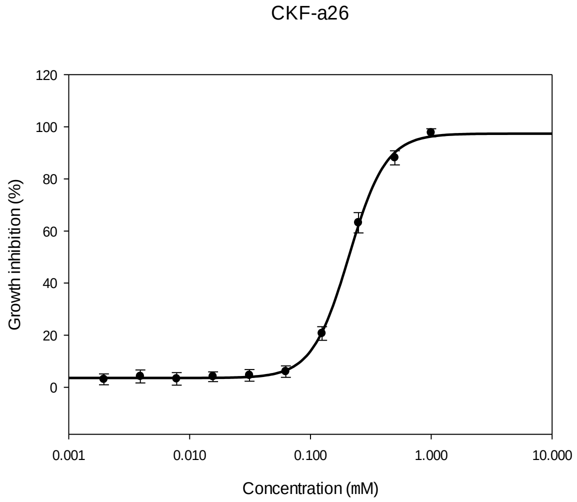
CKF-a26
Growth inhibition (%)
120
100
80
60
40
20
0
0.001
0.010
0.100
1.000
10.000
Concentration (mM)

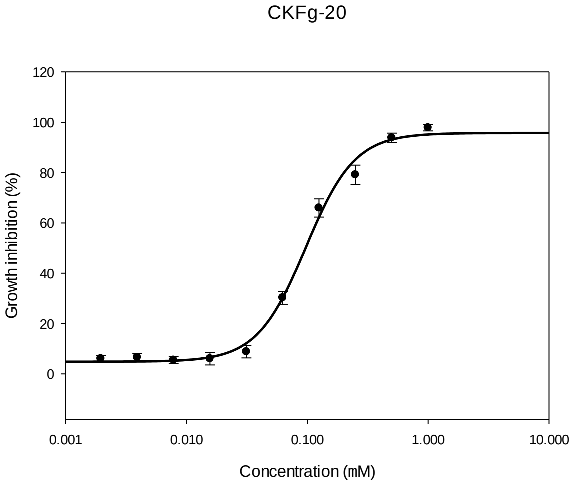
CKFg-20
Growth inhibition (%)
120
100
80
60
40
20
0
0.001
0.010
0.100
1.000
10.000
Concentration (mM)

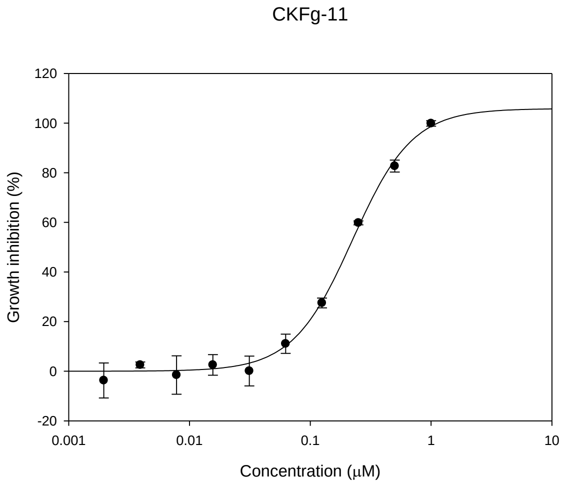
CKFg-11
Growth inhibition (%)
120
100
80
60
40
20
0
-20
0.001
0.01
0.1
1
10
Concentration (μM)

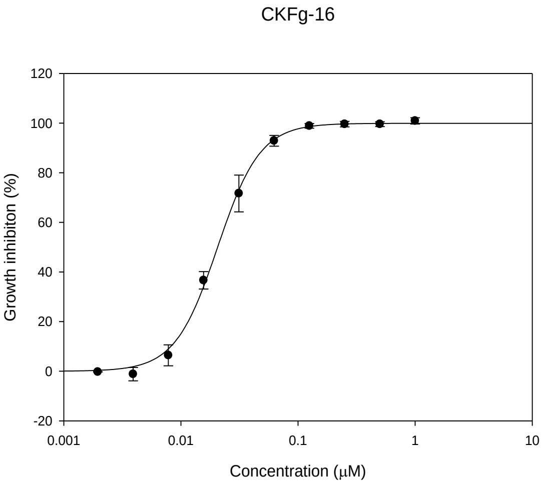
CKFg-16
Growth inhibiton (%)
120
100
80
60
40
20
0
-20
0.001
0.01
0.1
1
10
Concentration (μM)

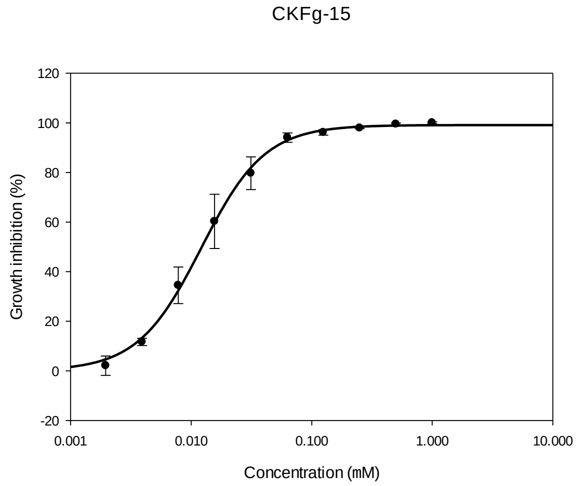
CKFg-15
Growth inhibition (%)
-20
0
20
40
60
80
100
120
0.001
0.010
0.100
1.000
10.000
Concentration (mM)

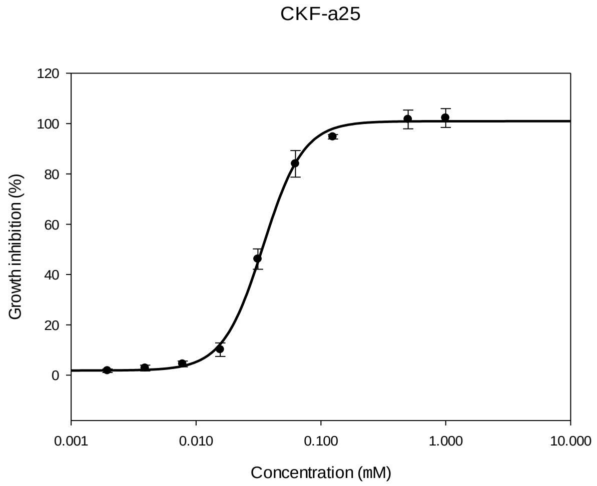
CKF-a25
Growth inhibition (%)
0
20
40
60
80
100
120
0.001
0.010
0.100
1.000
10.000
Concentration (mM)

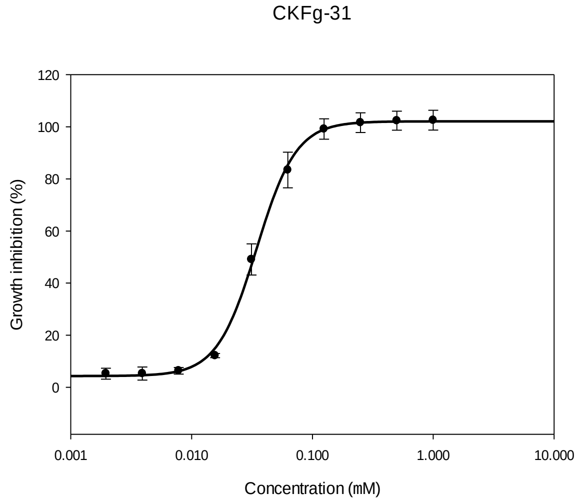
CKFg-31
Growth inhibition (%)
0
20
40
60
80
100
120
0.001
0.010
0.100
1.000
10.000
Concentration (mM)

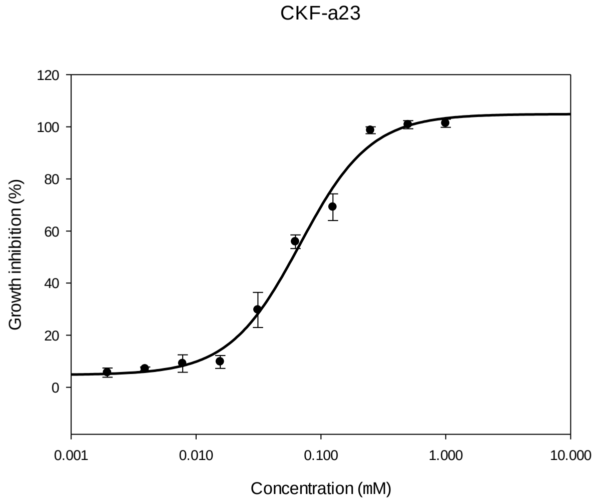
CKF-a23
Growth inhibition (%)
0
20
40
60
80
100
120
0.001
0.010
0.100
1.000
10.000
Concentration (mM)

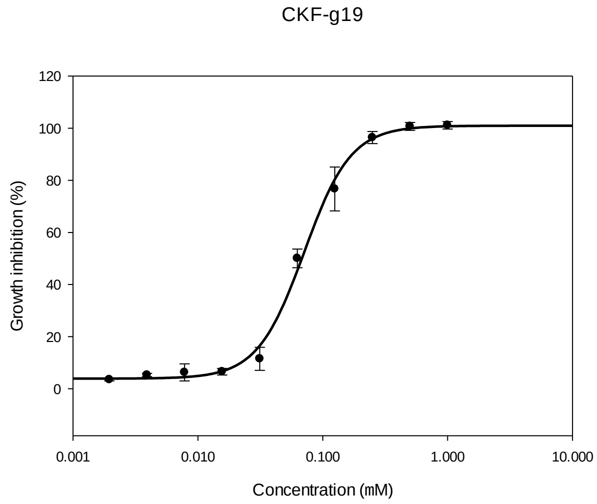
CKF-g19
Growth inhibition (%)
0
20
40
60
80
100
120
0.001
0.010
0.100
1.000
10.000
Concentration (mM)

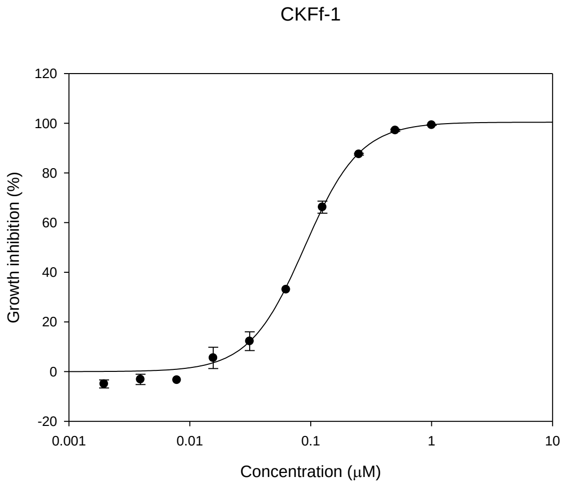
CKFf-1
Growth inhibition (%)
-20
0
20
40
60
80
100
120
0.001
0.01
0.1
1
10
Concentration (μM)

CKFf-7

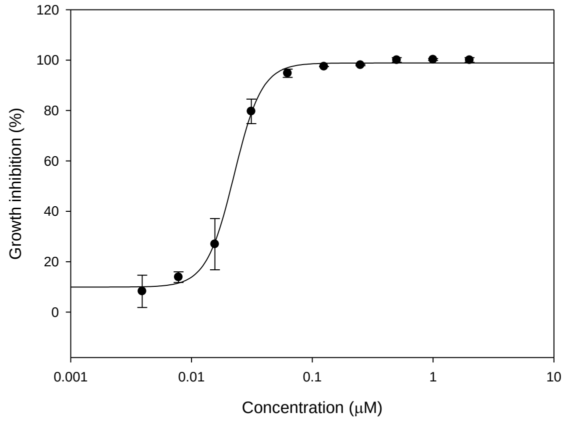

CKFf-3

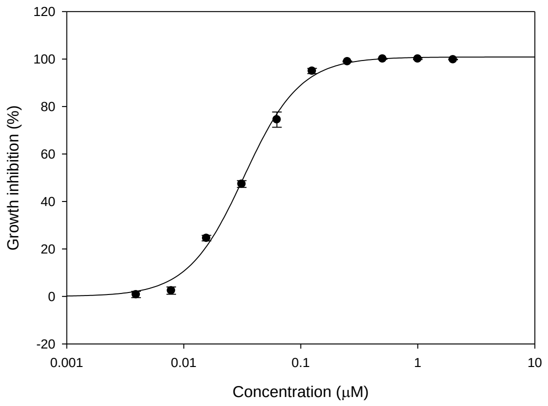

CKFa-33

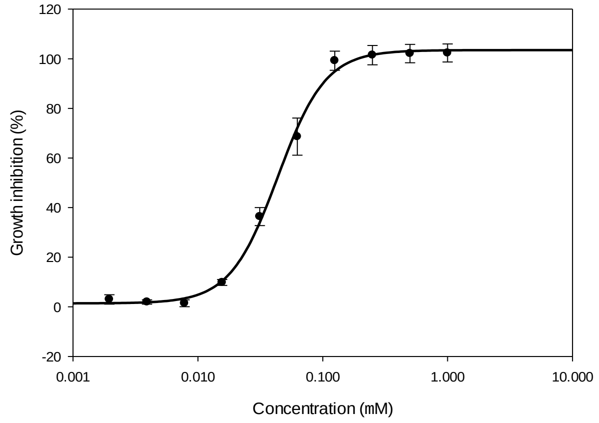

CKFa-29

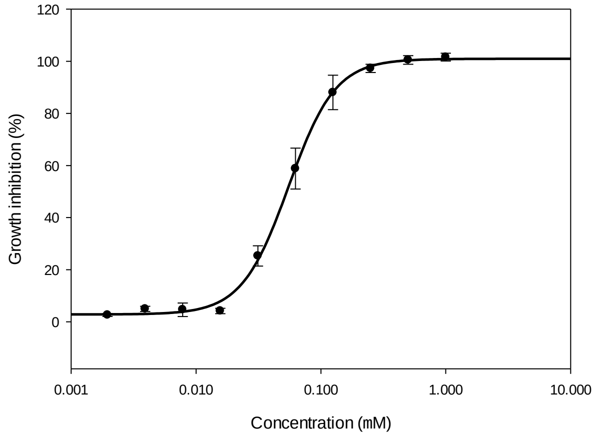

CKFf-35

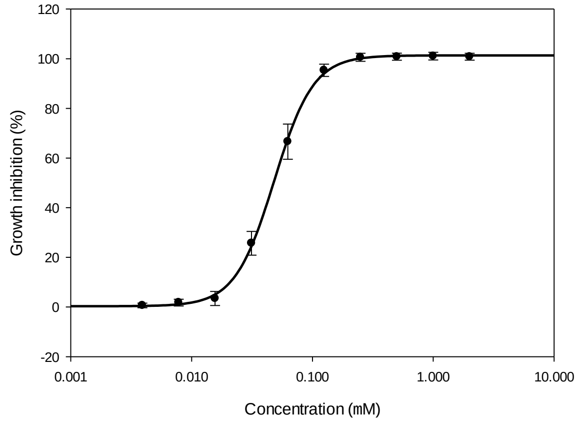

CKFf-2

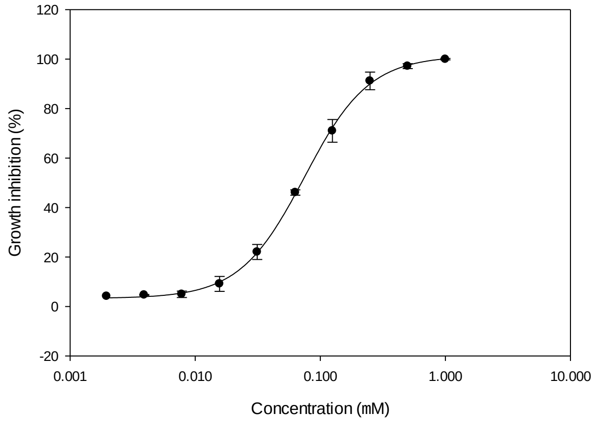

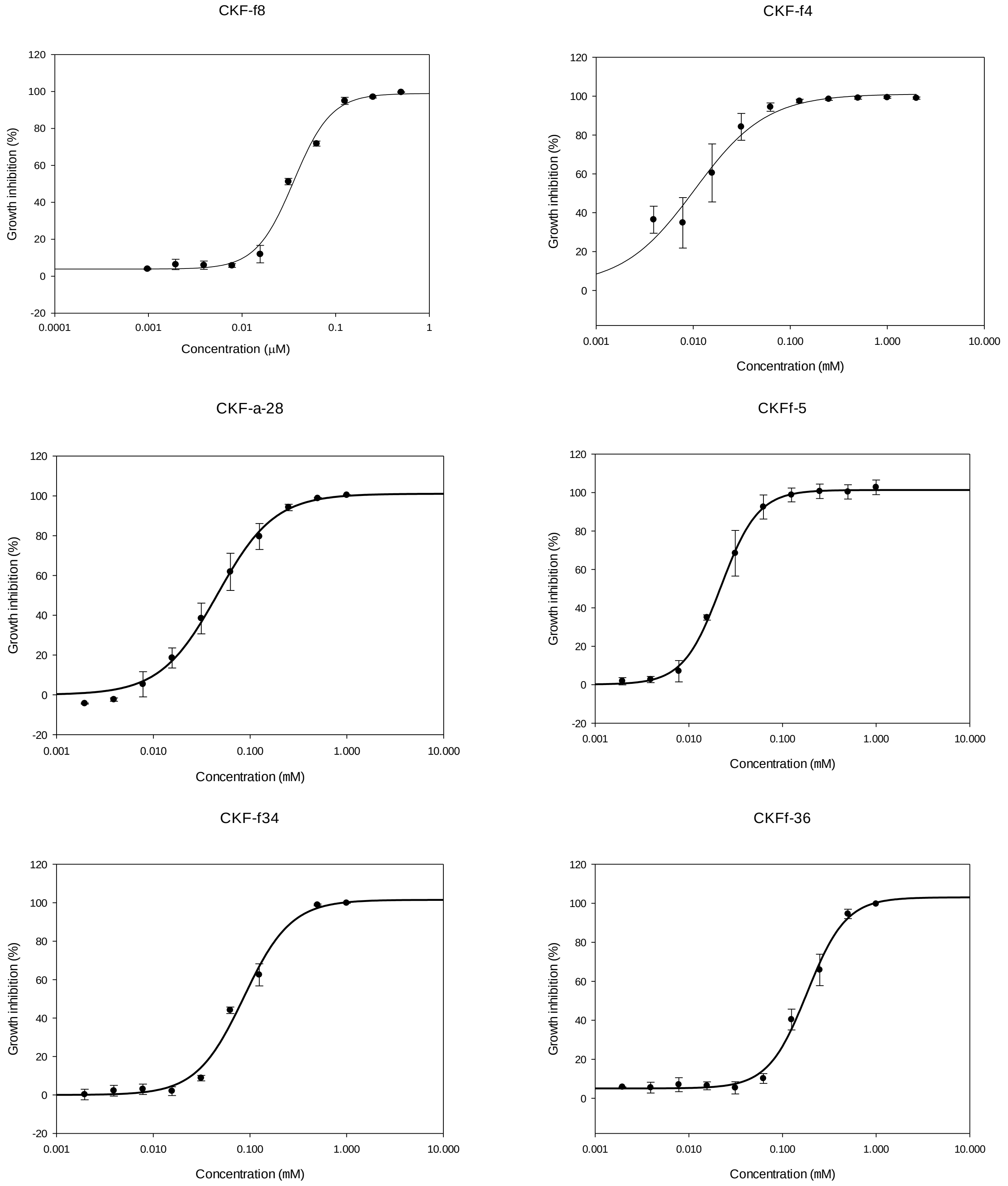

Figure S2. IC50 Curves.

Fa-M2

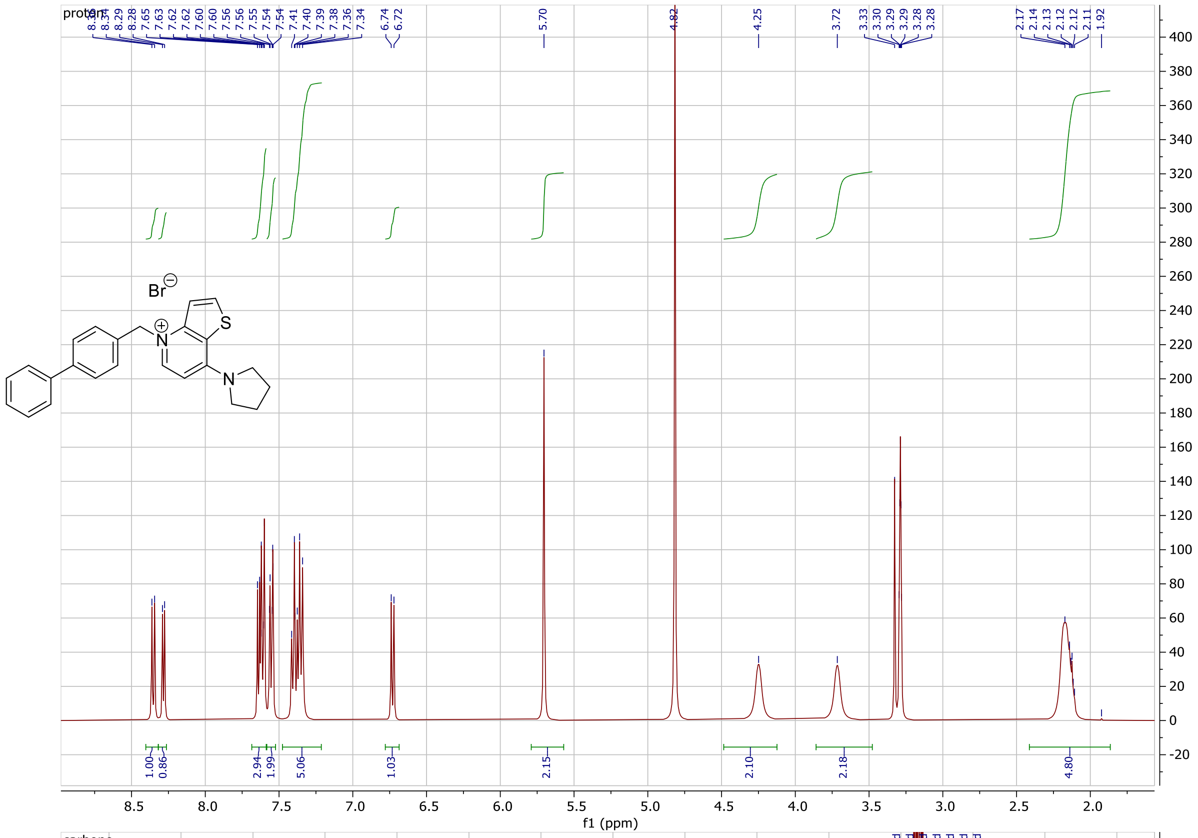
proton
8.37 8.35 8.29 8.28 7.65 7.63 7.62 7.62 7.60 7.60 7.56 7.55 7.54 7.54 7.41 7.40 7.39 7.38 7.36 7.34 6.74 6.72
5.70
4.82
4.25
3.72
3.33 3.30 3.29 3.29 3.28 3.28
2.17 2.14 2.13 2.12 2.12 2.11 1.92
1.00 0.86 2.94 1.99 5.06 1.03 2.15 2.10 2.18 4.80
Br
N
S
N
f1 (ppm)

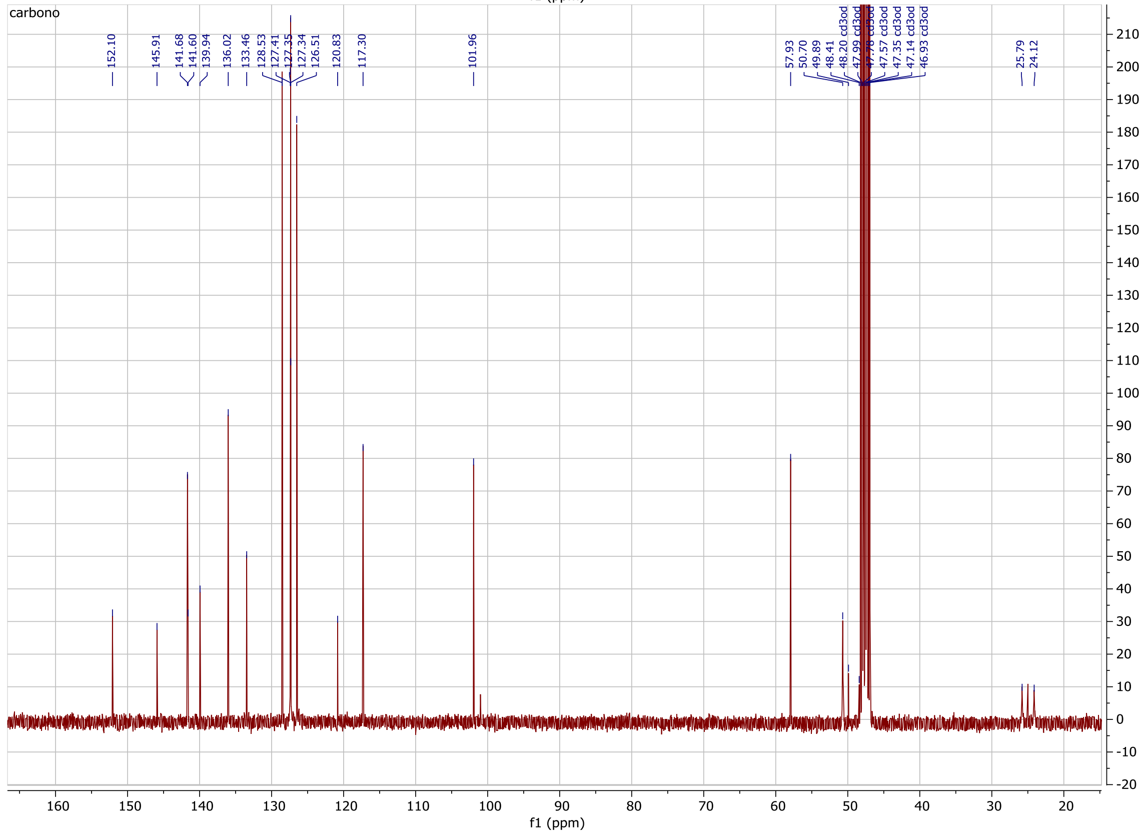
carbono
152.10
145.91
141.68 141.60 139.94
136.02
133.46
128.53 127.41 127.35 127.34 126.51
120.83
117.30
101.96
57.93 50.70 49.89 48.41 48.20 cd3od 47.99 cd3od 47.78 cd3od 47.57 cd3od 47.35 cd3od 47.14 cd3od 46.93 cd3od
25.79 24.12
f1 (ppm)

Fa-M1

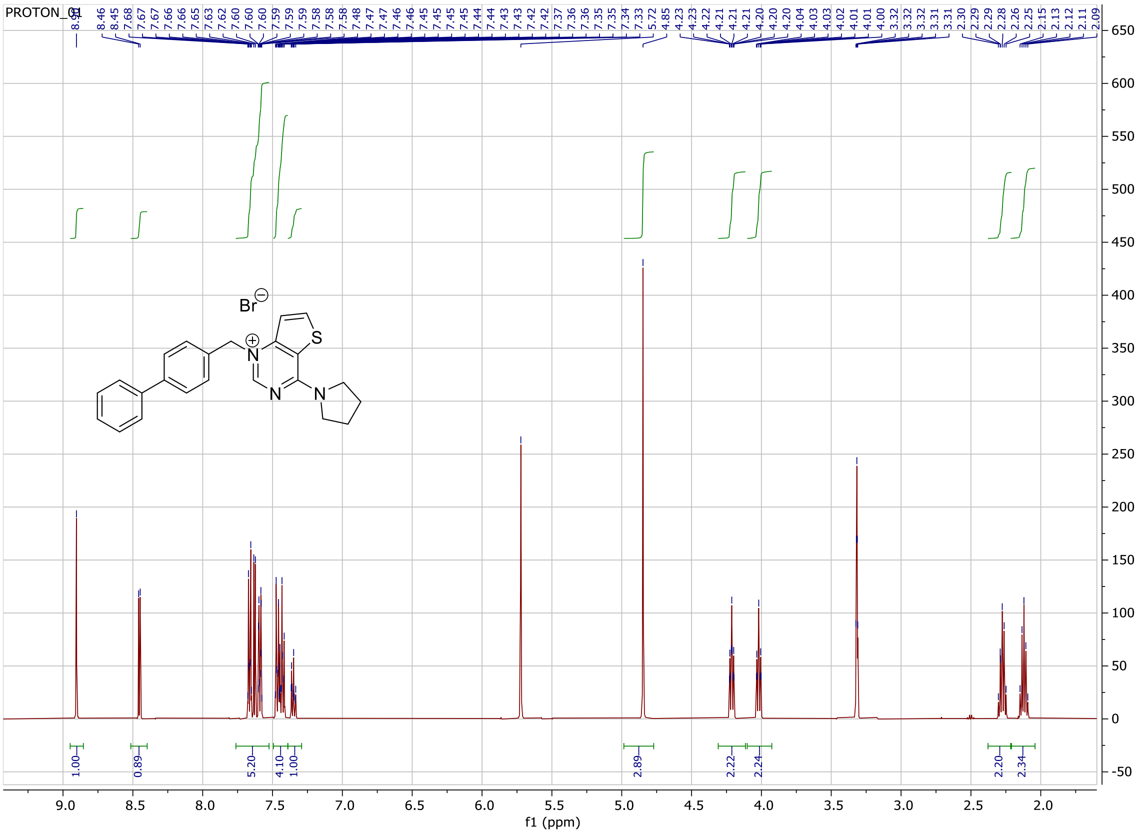

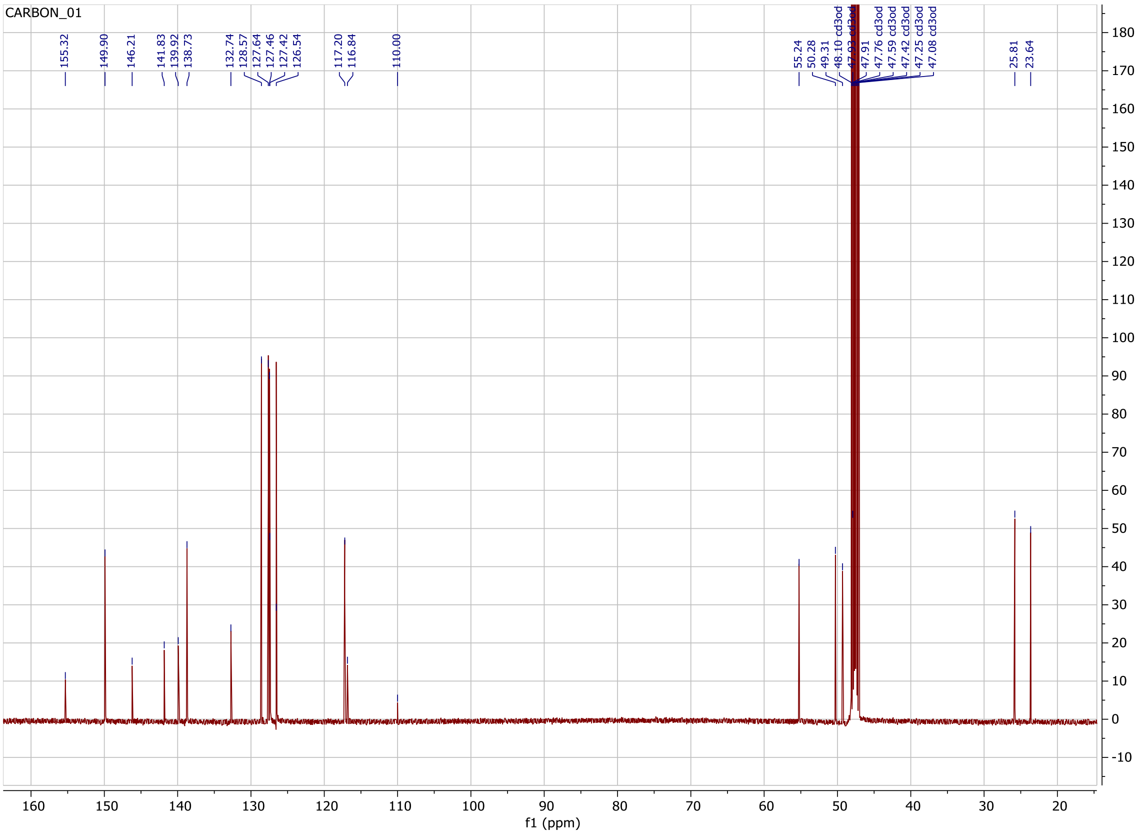

FMa-3

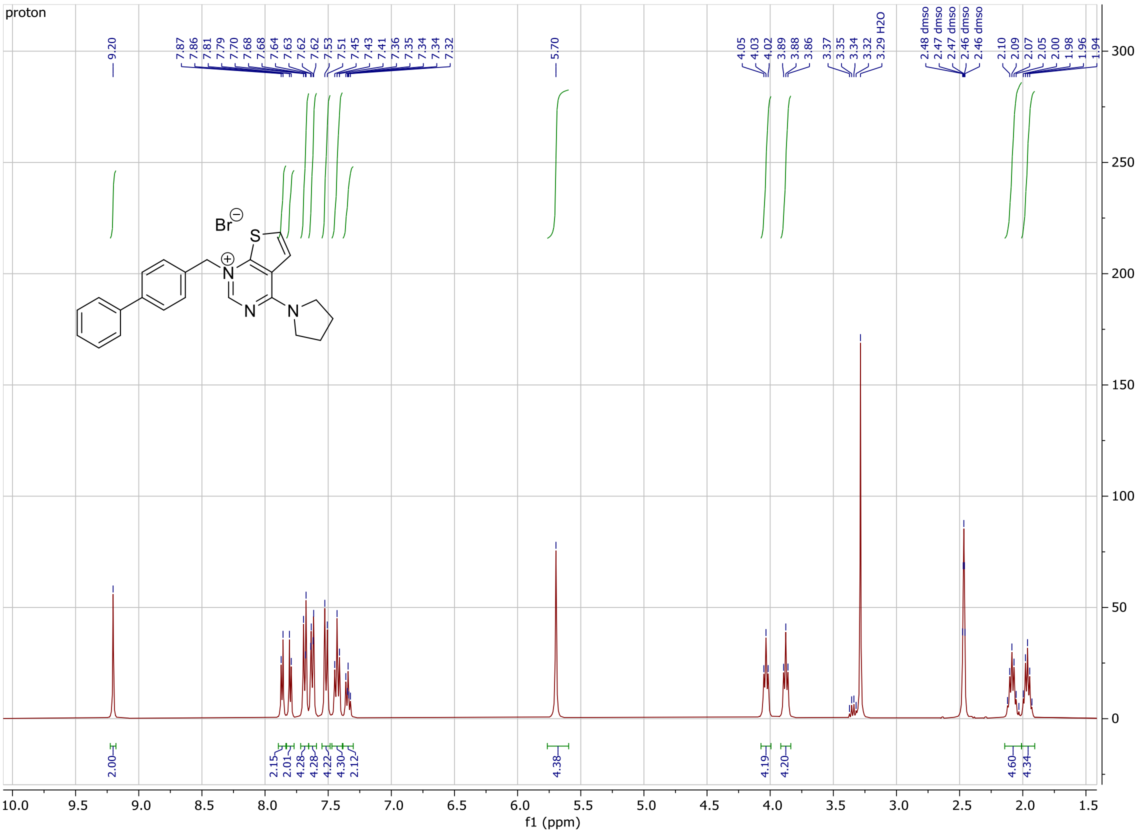
proton
9.20
7.87
7.86
7.81
7.79
7.70
7.68
7.64
7.63
7.62
7.53
7.51
7.45
7.43
7.41
7.36
7.35
7.34
7.34
7.32
5.70
4.05
4.03
4.02
3.89
3.88
3.86
3.37
3.35
3.34
3.32
3.29 H2O
2.48 dmso
2.47 dmso
2.47 dmso
2.46 dmso
2.46 dmso
2.10
2.09
2.07
2.05
2.00
1.98
1.96
1.94
Br
S
N
N
N
2.00
2.15
2.01
4.28
4.28
4.22
4.30
2.12
4.38
4.19
4.20
4.60
4.34
f1 (ppm)

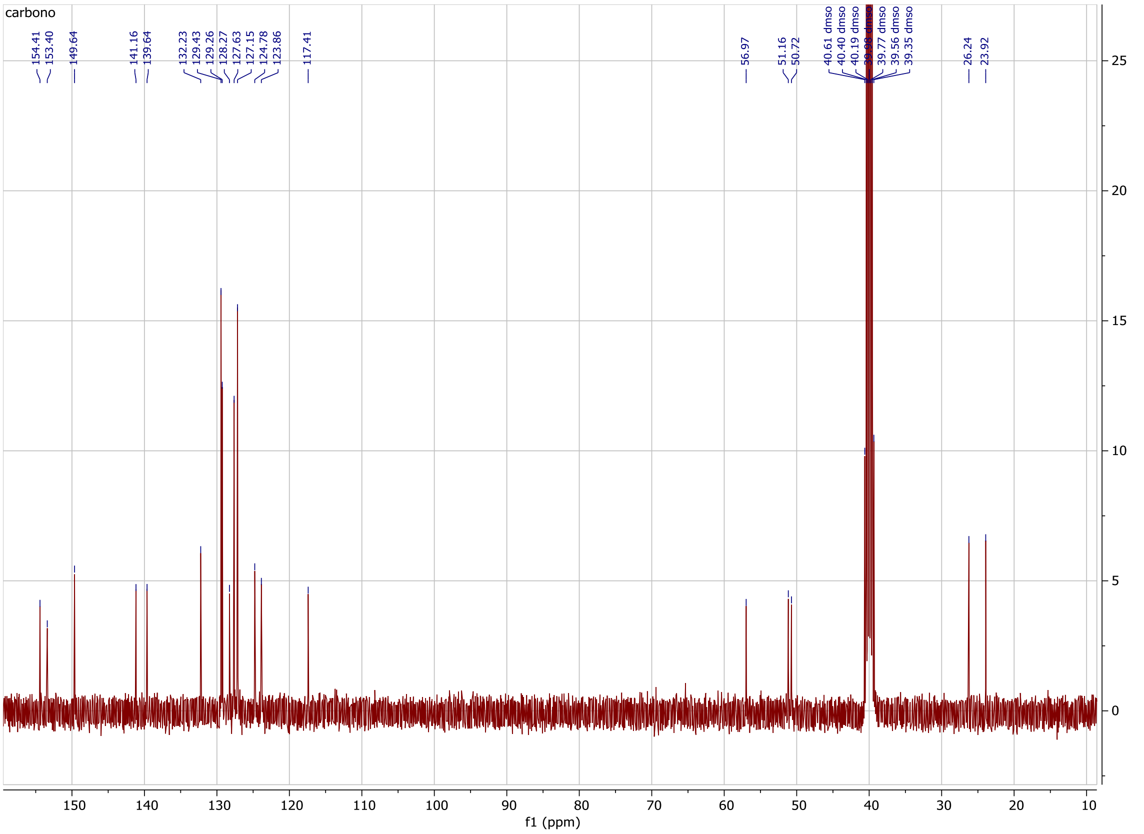
carbono
154.41
153.40
149.64
141.16
139.64
132.23
129.43
129.26
128.27
127.63
127.15
124.78
123.86
117.41
56.97
51.16
50.72
40.61 dmso
40.40 dmso
40.19 dmso
39.77 dmso
39.56 dmso
39.35 dmso
26.24
23.92
f1 (ppm)

Fg-9

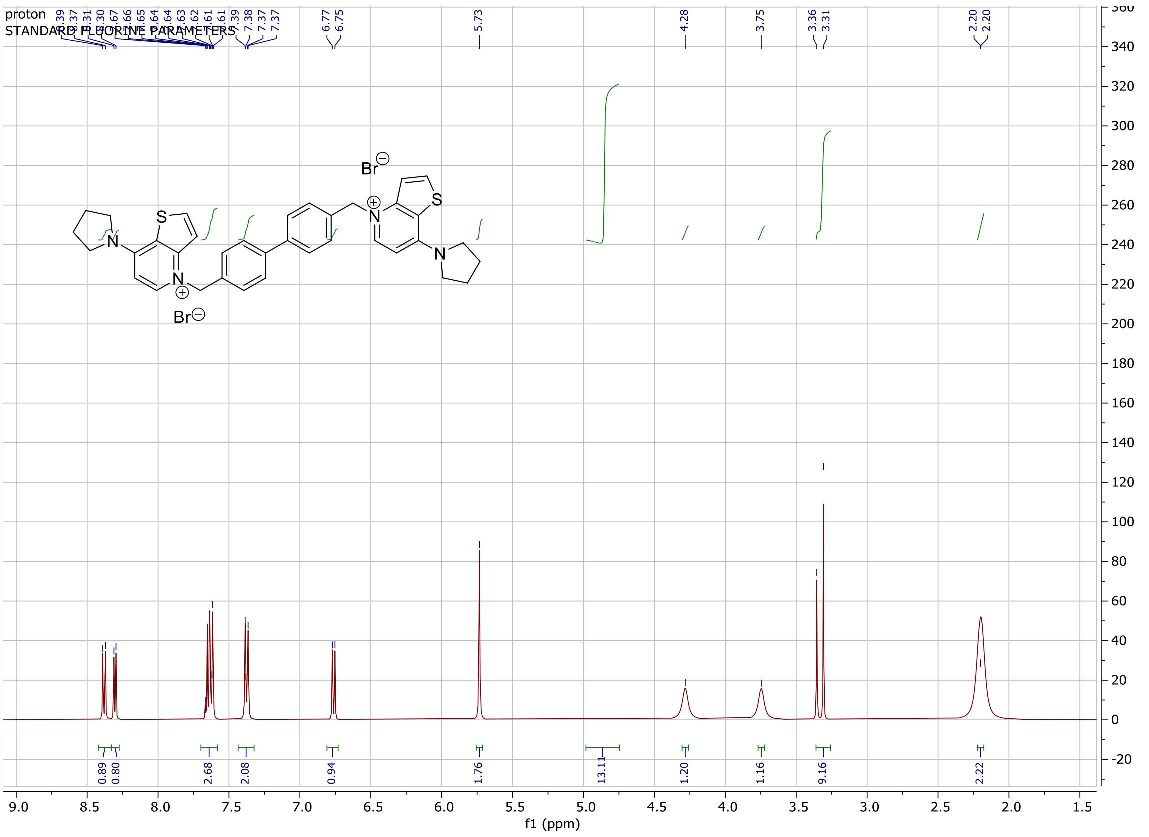
proton
STANDARD FLUORINE PARAMETERS
6.77
6.75
5.73
4.28
3.75
3.36
3.31
2.20
2.20
Br
S
N
N
Br
0.89
0.80
2.68
2.08
0.94
1.76
13.11
1.20
1.16
9.16
2.22
f1 (ppm)

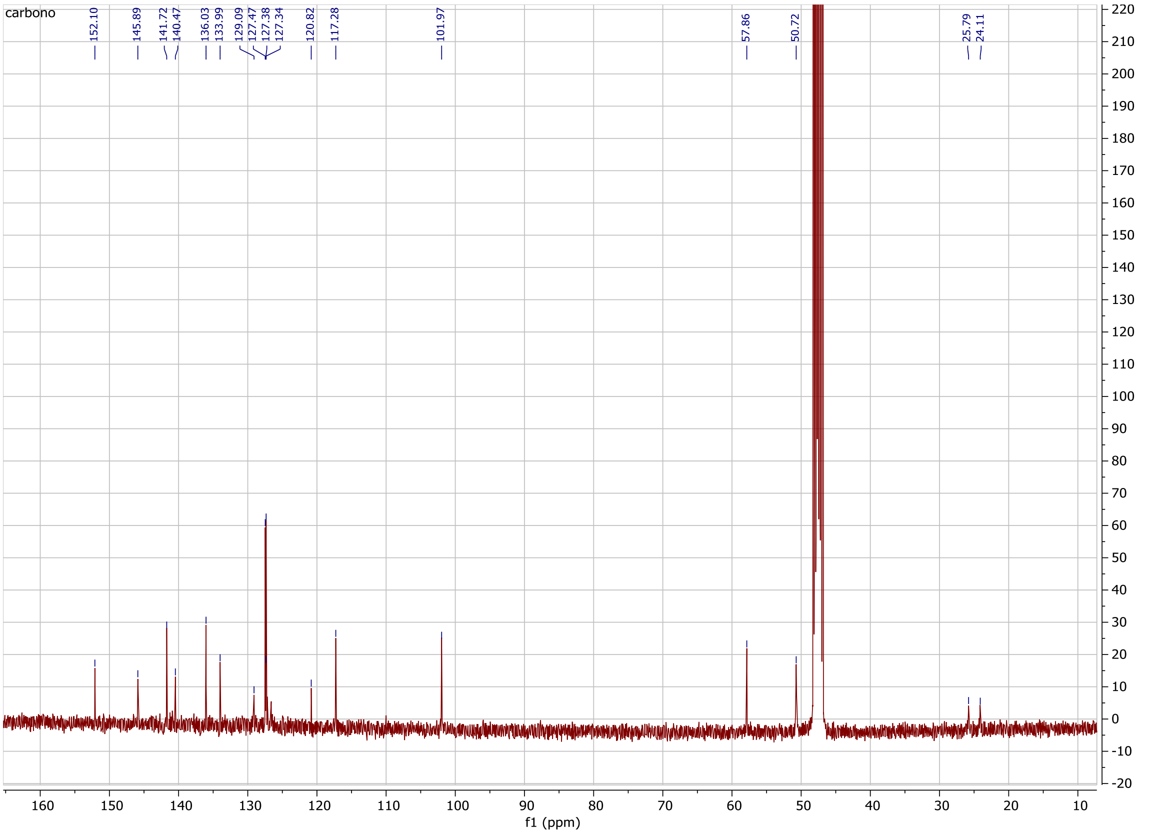
carbono
152.10
145.89
141.72
140.47
136.03
133.99
129.09
127.47
127.38
127.34
120.82
117.28
101.97
57.86
50.72
25.79
24.11
f1 (ppm)

Fa-21

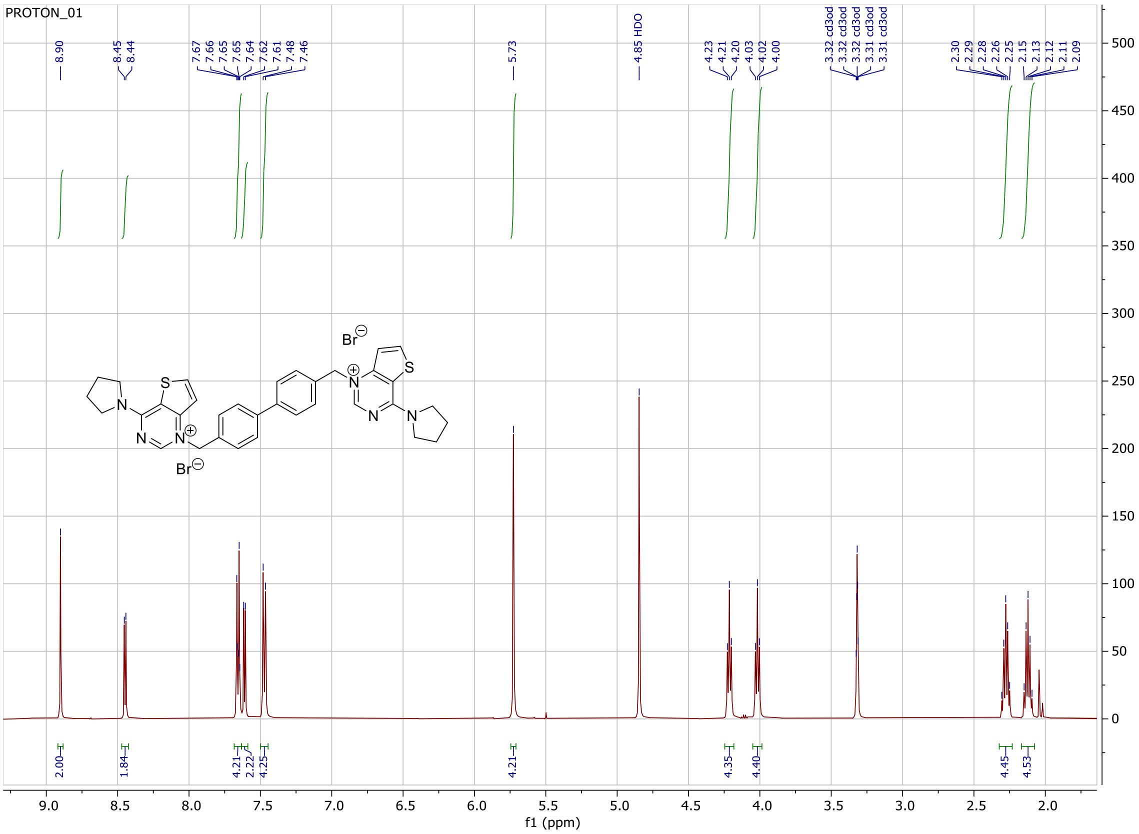
PROTON_01
8.90
8.45
8.44
7.67
7.66
7.65
7.65
7.64
7.62
7.61
7.48
7.46
5.73
4.85 HDO
4.23
4.21
4.20
4.03
4.02
4.00
3.32 cd3od
3.32 cd3od
3.32 cd3od
3.31 cd3od
3.31 cd3od
2.30
2.29
2.28
2.26
2.25
2.15
2.13
2.12
2.11
2.09
Br⊖
Br⊖
S
S
N
N
N
N⊕
N⊕
N
2.00
1.84
4.21
2.22
4.25
4.21
4.35
4.40
4.45
4.53
f1 (ppm)

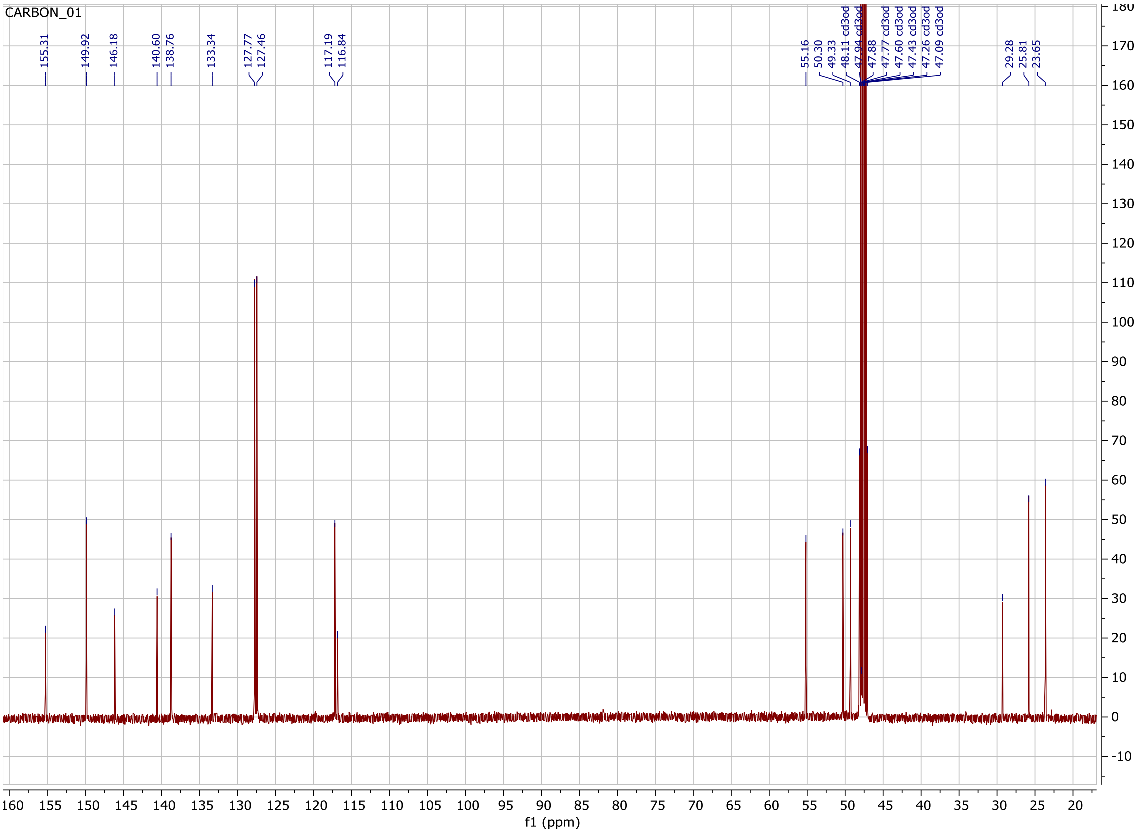
CARBON_01
155.31
149.92
146.18
140.60
138.76
133.34
127.77
127.46
117.19
116.84
55.16
50.30
49.33
48.11 cd3od
47.94 cd3od
47.88
47.77 cd3od
47.60 cd3od
47.43 cd3od
47.26 cd3od
47.09 cd3od
29.28
25.81
23.65
f1 (ppm)

Fg-14

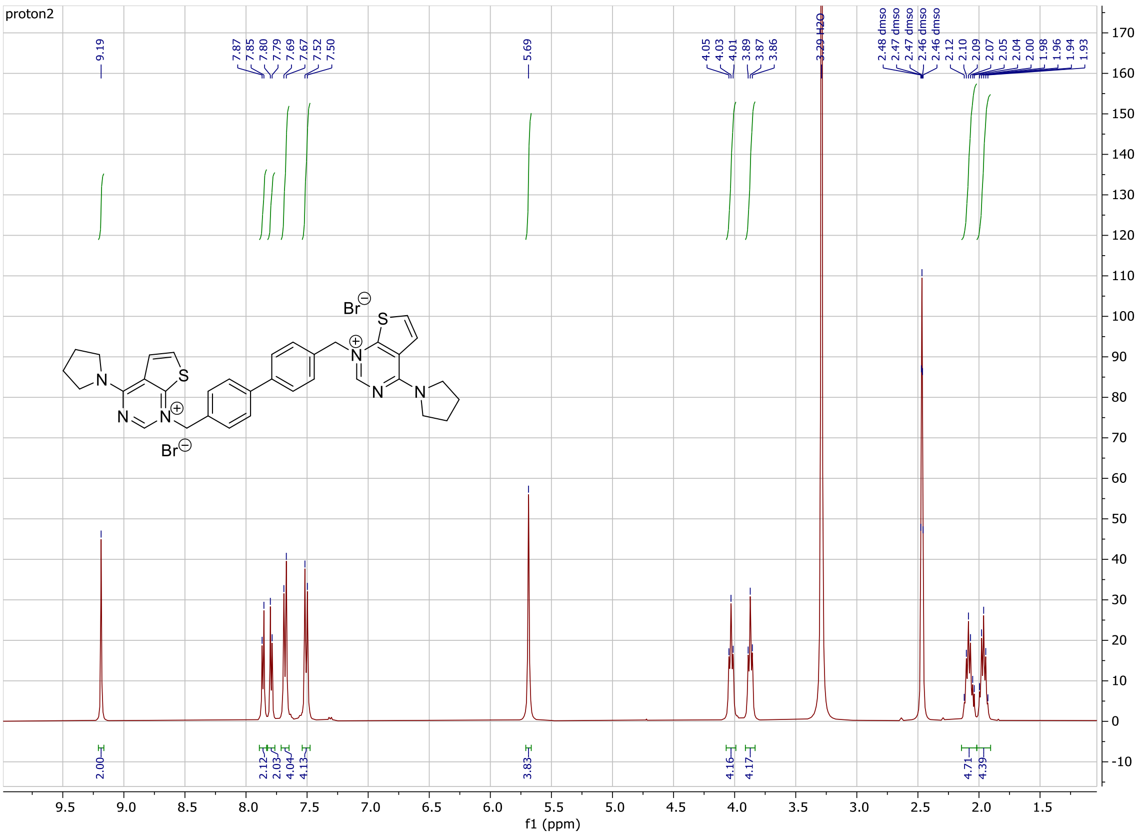

proton2
9.19
7.87
7.85
7.80
7.79
7.69
7.67
7.52
7.50
5.69
4.05
4.03
4.01
3.89
3.87
3.86
3.29 H2O
2.48 dmso
2.47 dmso
2.47 dmso
2.46 dmso
2.46 dmso
2.12
2.10
2.09
2.07
2.05
2.04
2.00
1.98
1.96
1.94
1.93
2.00
2.12
2.03
4.04
4.13
3.83
4.16
4.17
4.71
4.39
Br
S
N
N
N
f1 (ppm)

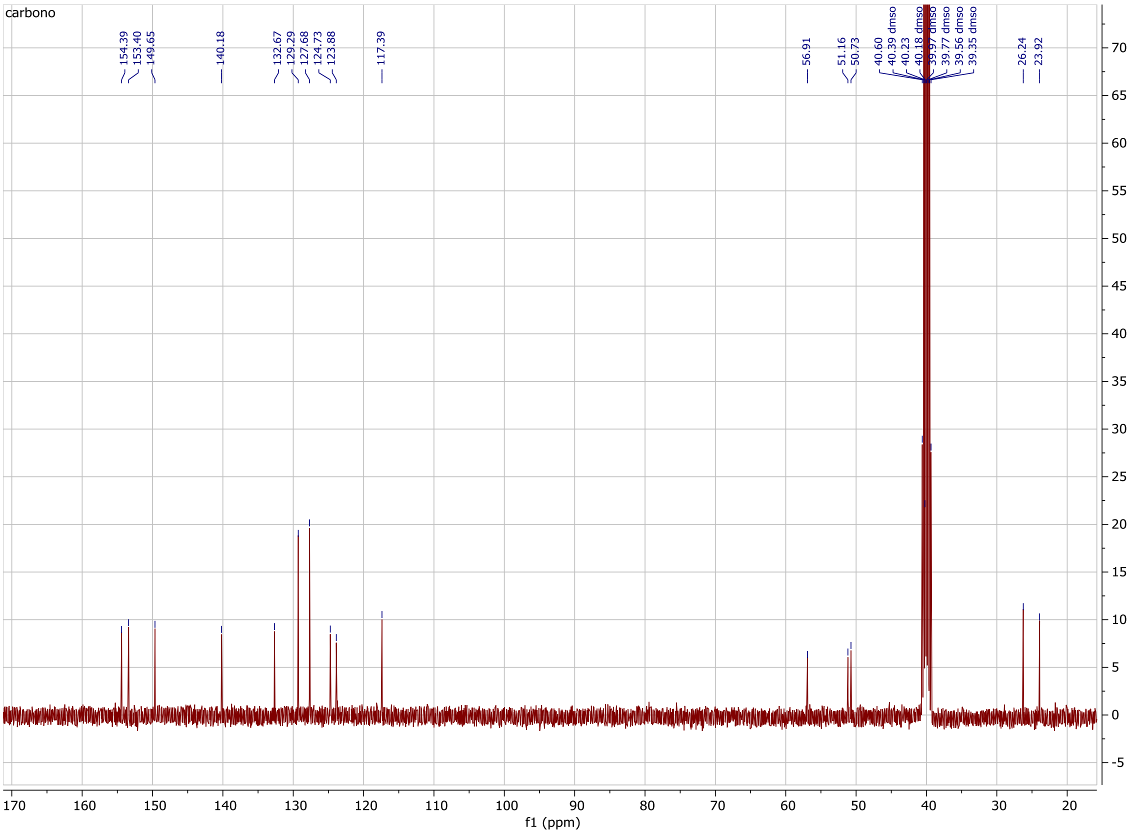

carbono
154.39
153.40
149.65
140.18
132.67
129.29
127.68
124.73
123.88
117.39
56.91
51.16
50.73
40.60
40.39 dmso
40.23
40.18 dmso
39.97 dmso
39.77 dmso
39.56 dmso
39.35 dmso
26.24
23.92
f1 (ppm)

Fa-24

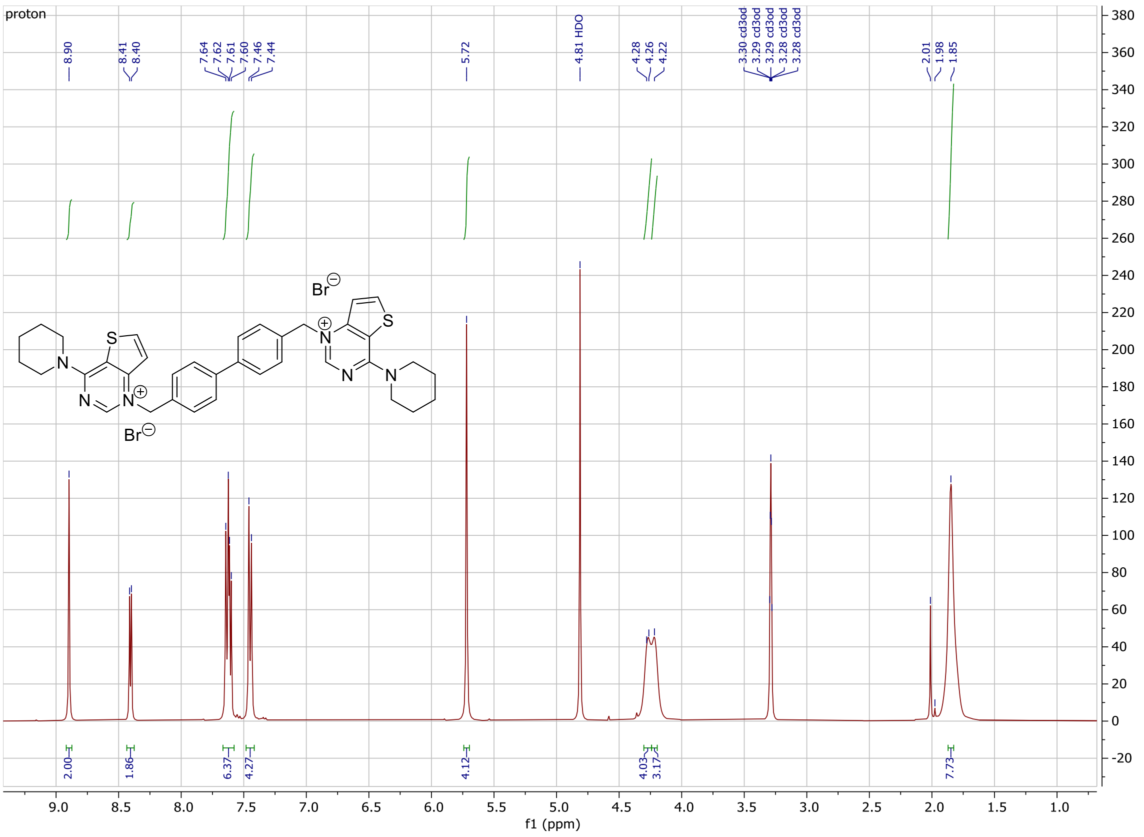
proton
8.90
8.41
8.40
7.64
7.62
7.61
7.60
7.46
7.44
5.72
4.81 HDO
4.28
4.26
4.22
3.30 cd3od
3.29 cd3od
3.29 cd3od
3.28 cd3od
3.28 cd3od
2.01
1.98
1.85
Br⊖
Br⊖
S
S
N
N
N
N
N
N
2.00
1.86
6.37
4.27
4.12
4.03
3.17
7.73
f1 (ppm)

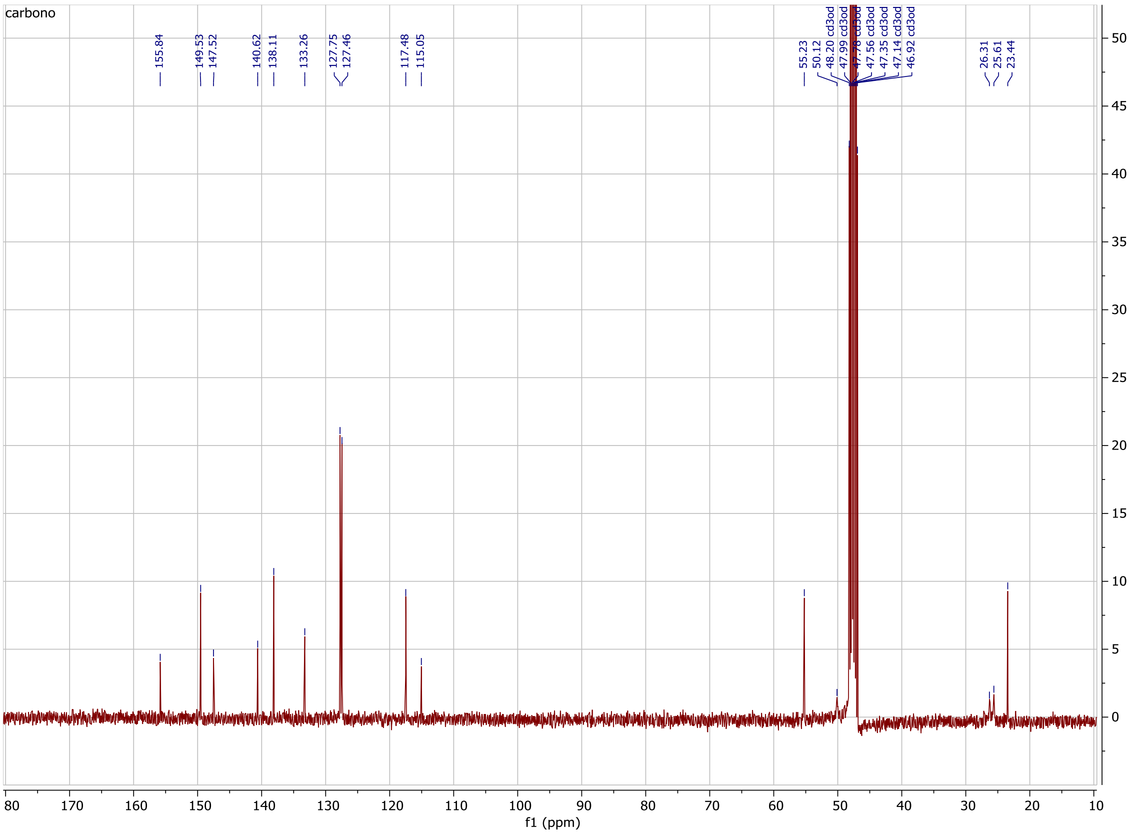
carbono
155.84
149.53
147.52
140.62
138.11
133.26
127.75
127.46
117.48
115.05
55.23
50.12
48.20 cd3od
47.99 cd3od
47.78 cd3od
47.56 cd3od
47.35 cd3od
47.14 cd3od
46.92 cd3od
26.31
25.61
23.44
f1 (ppm)

Fg-30

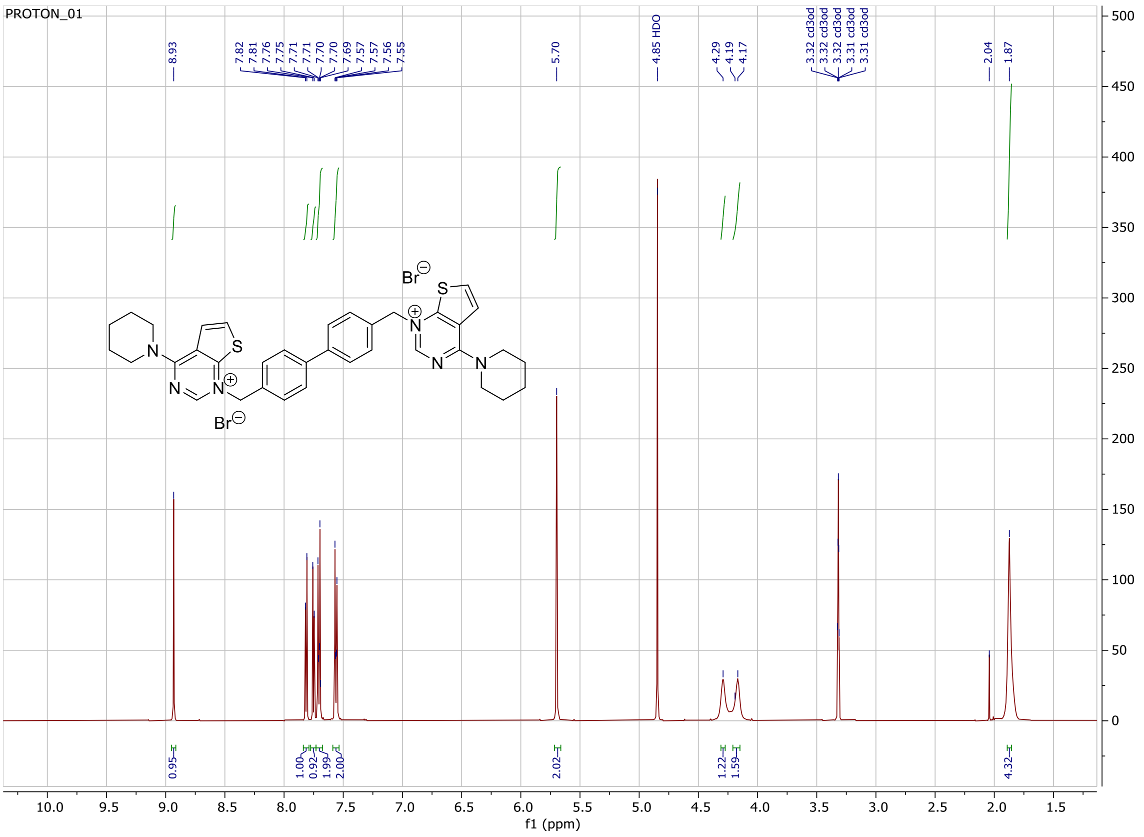
PROTON_01
8.93
7.82
7.81
7.76
7.75
7.71
7.71
7.70
7.70
7.69
7.57
7.57
7.56
7.55
5.70
4.85 HDO
4.29
4.19
4.17
3.32 cd3od
3.32 cd3od
3.32 cd3od
3.31 cd3od
3.31 cd3od
2.04
1.87
0.95
1.00
0.92
1.99
2.00
2.02
1.22
1.59
4.32
Br
S
N
N
N
f1 (ppm)

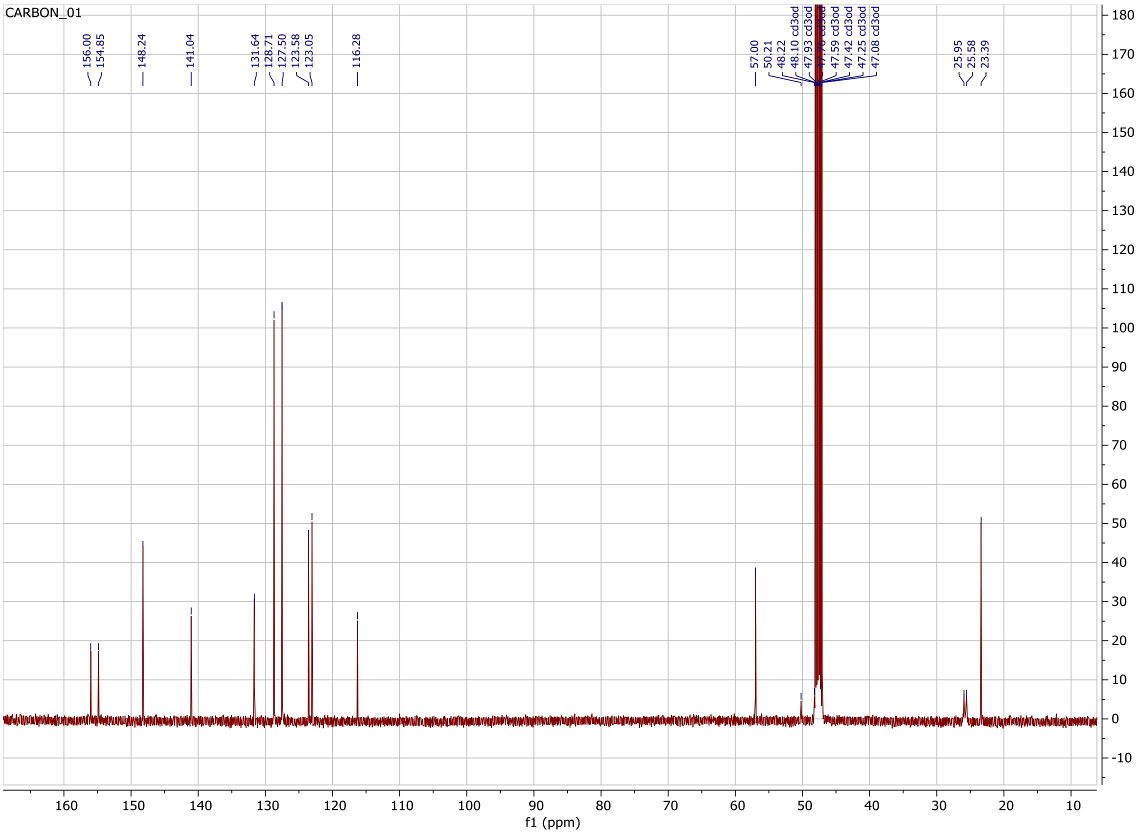
CARBON_01
156.00
154.85
148.24
141.04
131.64
128.71
127.50
123.58
123.05
116.28
57.00
50.21
48.22
48.10 cd3od
47.93 cd3od
47.59 cd3od
47.42 cd3od
47.25 cd3od
47.08 cd3od
25.95
25.58
23.39
f1 (ppm)

Fa-22

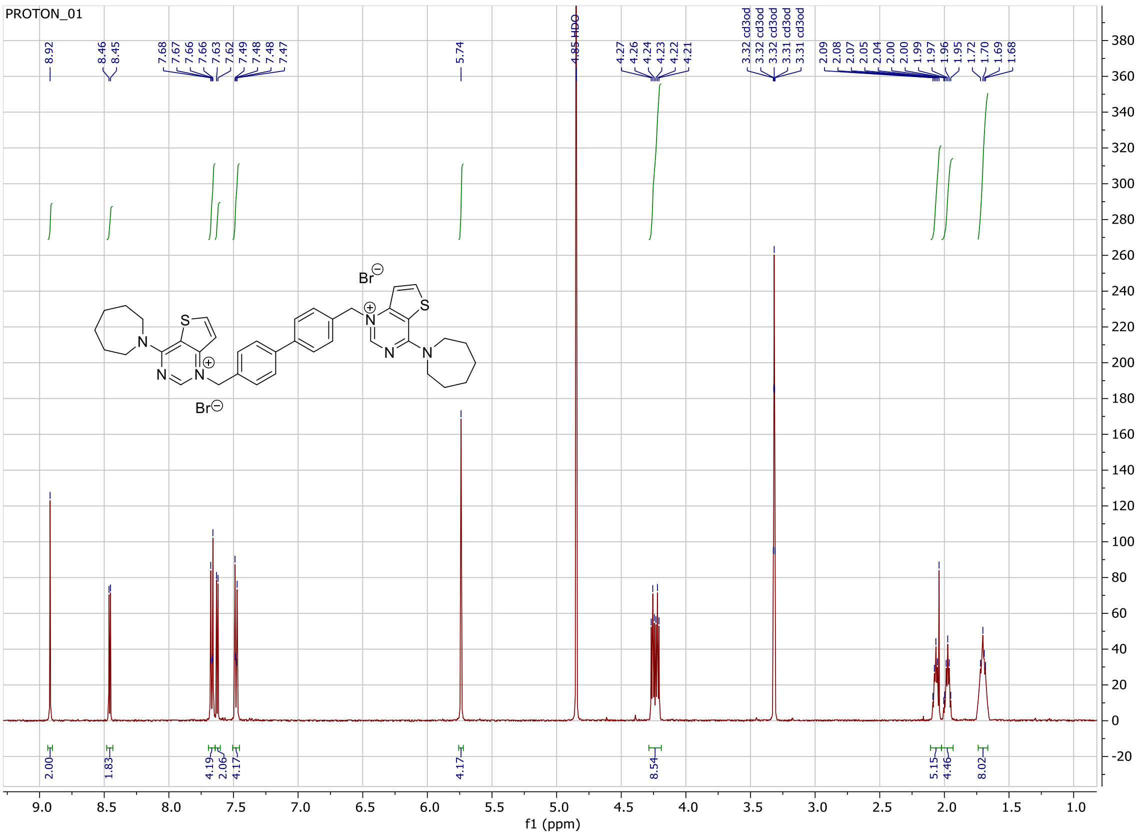
PROTON_01
8.92
8.46
8.45
7.68
7.67
7.66
7.66
7.63
7.62
7.49
7.48
7.48
7.47
5.74
4.85 HDO
4.27
4.26
4.24
4.23
4.22
4.21
3.32 cd3od
3.32 cd3od
3.32 cd3od
3.31 cd3od
3.31 cd3od
2.09
2.08
2.07
2.05
2.04
2.00
2.00
1.99
1.97
1.96
1.95
1.72
1.70
1.69
1.68
2.00
1.83
4.19
2.06
4.17
4.17
8.54
5.15
4.46
8.02
Br⊖
S
N
N
N
N
S
N
N
Br⊖
f1 (ppm)

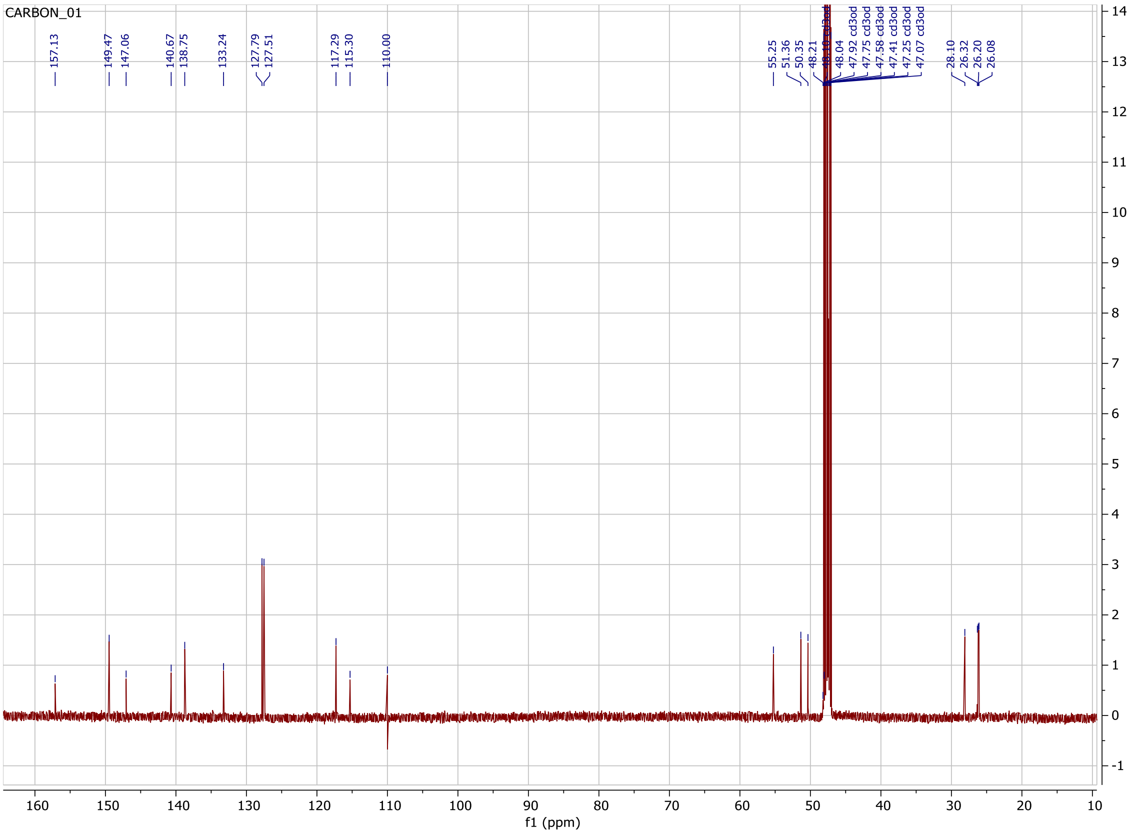
CARBON_01
157.13
149.47
147.06
140.67
138.75
133.24
127.79
127.51
117.29
115.30
110.00
55.25
51.36
50.35
48.21
48.04
47.92 cd3od
47.75 cd3od
47.58 cd3od
47.41 cd3od
47.25 cd3od
47.07 cd3od
28.10
26.32
26.20
26.08
f1 (ppm)

Fg-18

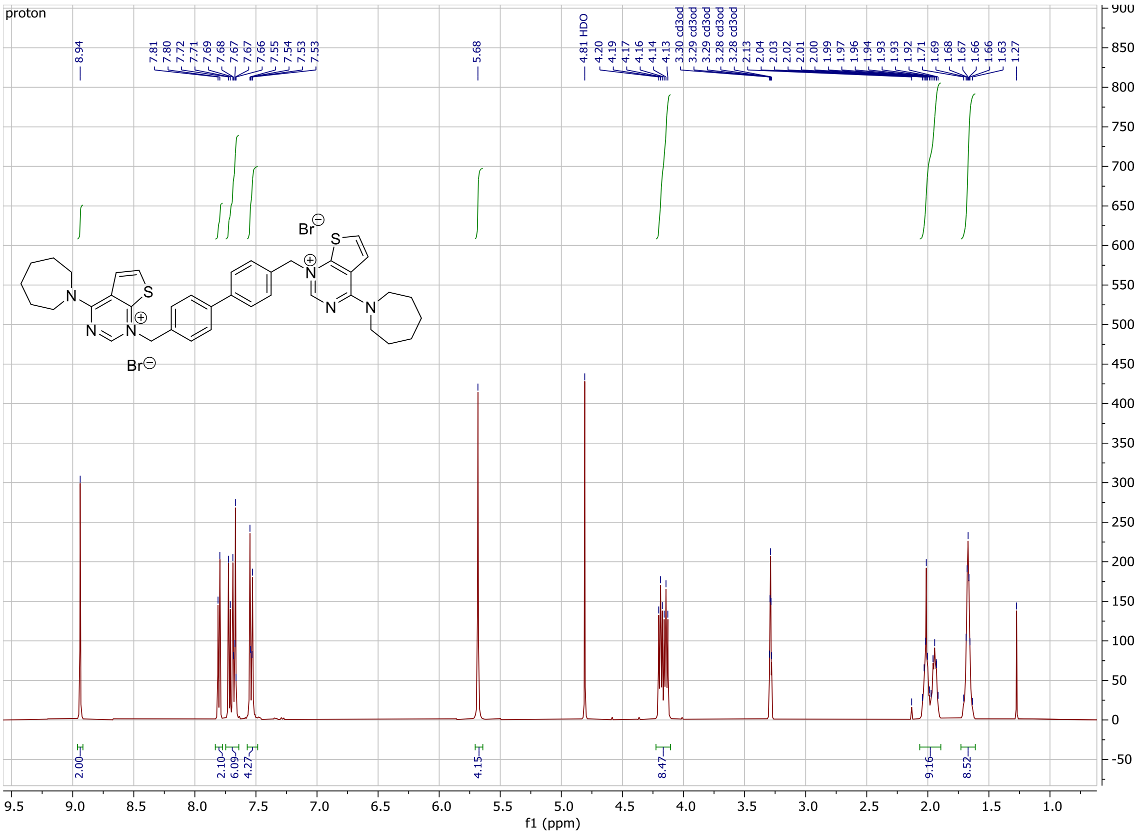

proton
8.94
7.81 7.80 7.72 7.71 7.69 7.68 7.67 7.67 7.66 7.55 7.54 7.53 7.53
5.68
4.81 HDO
4.20 4.19 4.17 4.16 4.14 4.13
3.30 cd3od 3.29 cd3od 3.29 cd3od 3.28 cd3od 3.28 cd3od
2.13 2.04 2.03 2.02 2.01 2.00 1.99 1.97 1.96 1.94 1.93 1.93 1.92 1.71 1.69 1.68 1.67 1.66 1.66 1.63 1.27
2.00
2.10
6.09
4.27
4.15
8.47
9.16
8.52
Br
S
N
N
N
f1 (ppm)

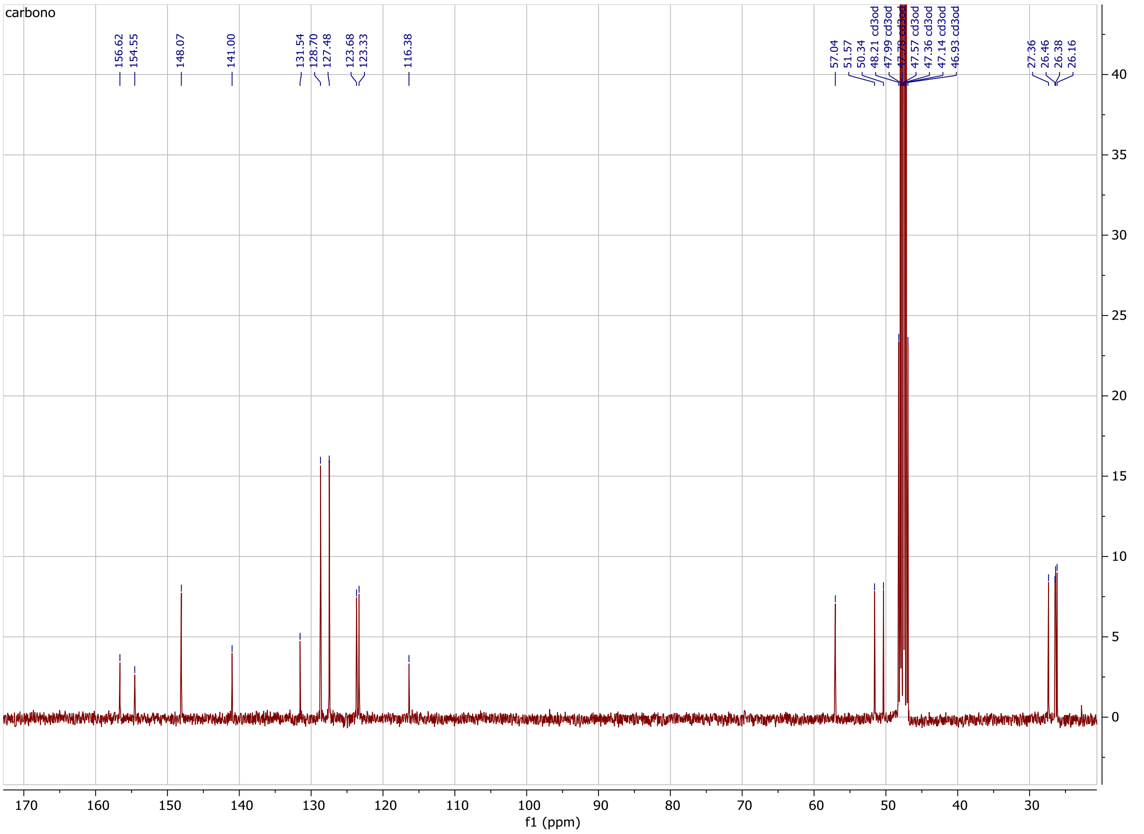

carbono
156.62 154.55
148.07
141.00
131.54 128.70 127.48 123.68 123.33
116.38
57.04 51.57 50.34 48.21 cd3od 47.99 cd3od 47.78 cd3od 47.57 cd3od 47.36 cd3od 47.14 cd3od 46.93 cd3od
27.36 26.46 26.38 26.16
f1 (ppm)

Fp-1

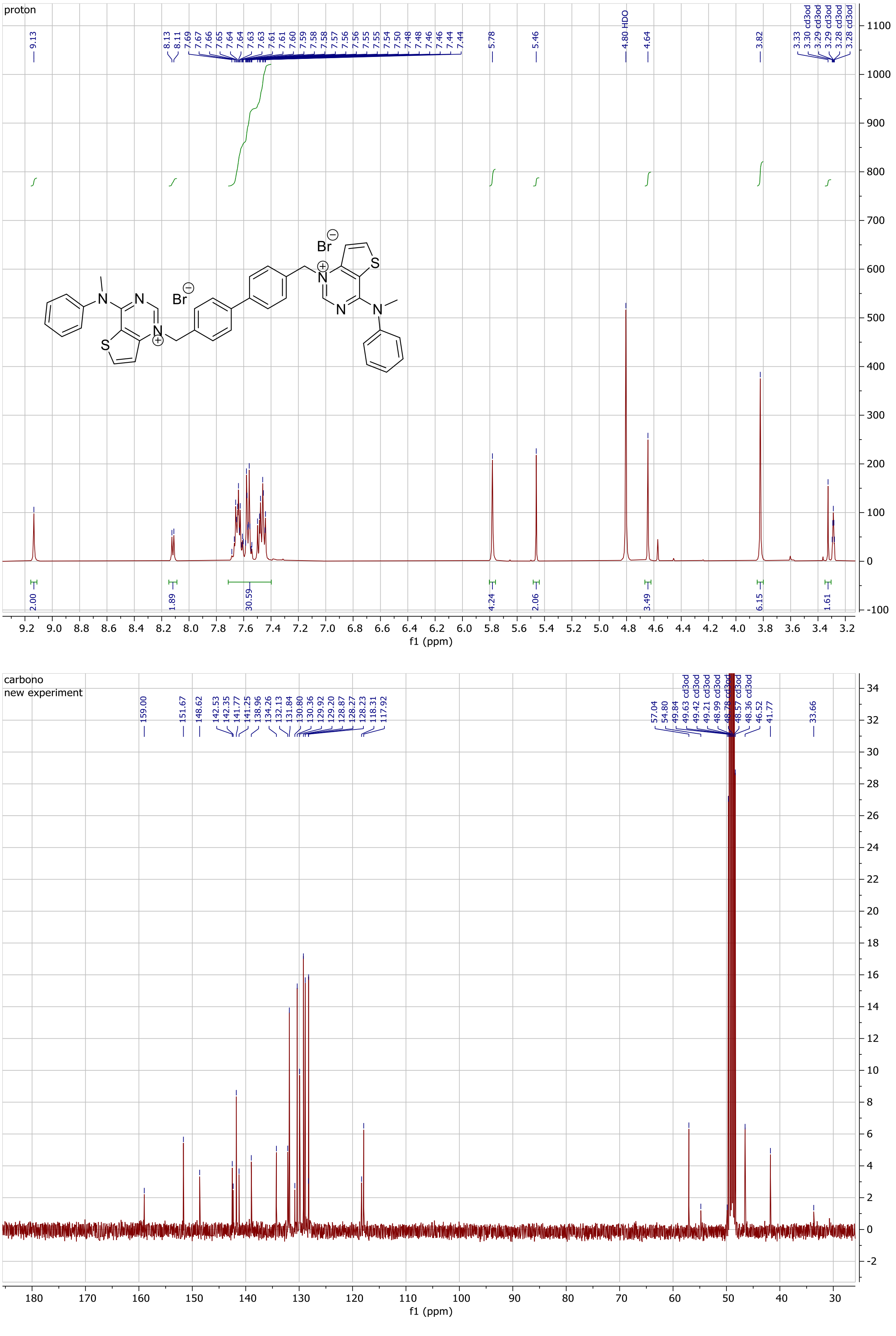

Fp-8

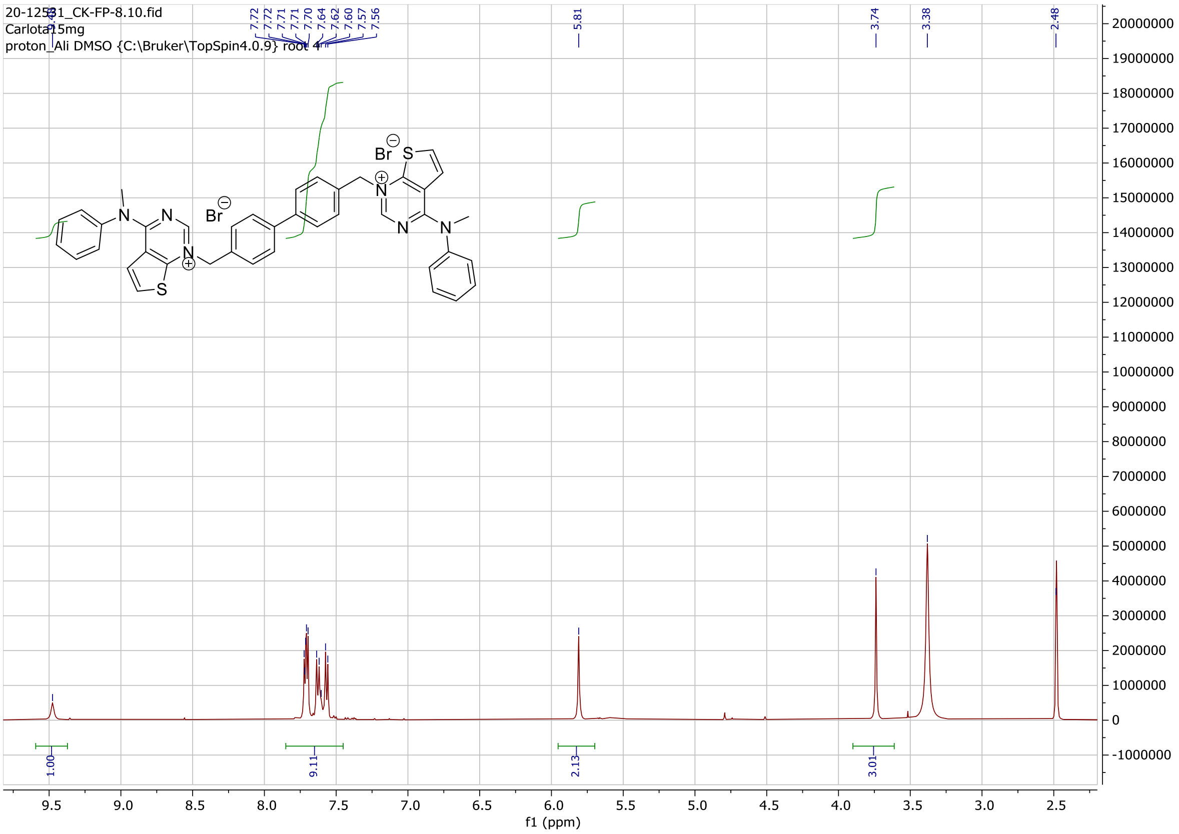

20-12531_CK-FP-8.10.fid
Carlota 15mg
proton_Ali DMSO {C:\Bruker\TopSpin4.0.9} root 4
7.72
7.72
7.71
7.71
7.70
7.64
7.62
7.60
7.57
7.56
5.81
3.74
3.38
2.48
1.00
9.11
2.13
3.01
f1 (ppm)

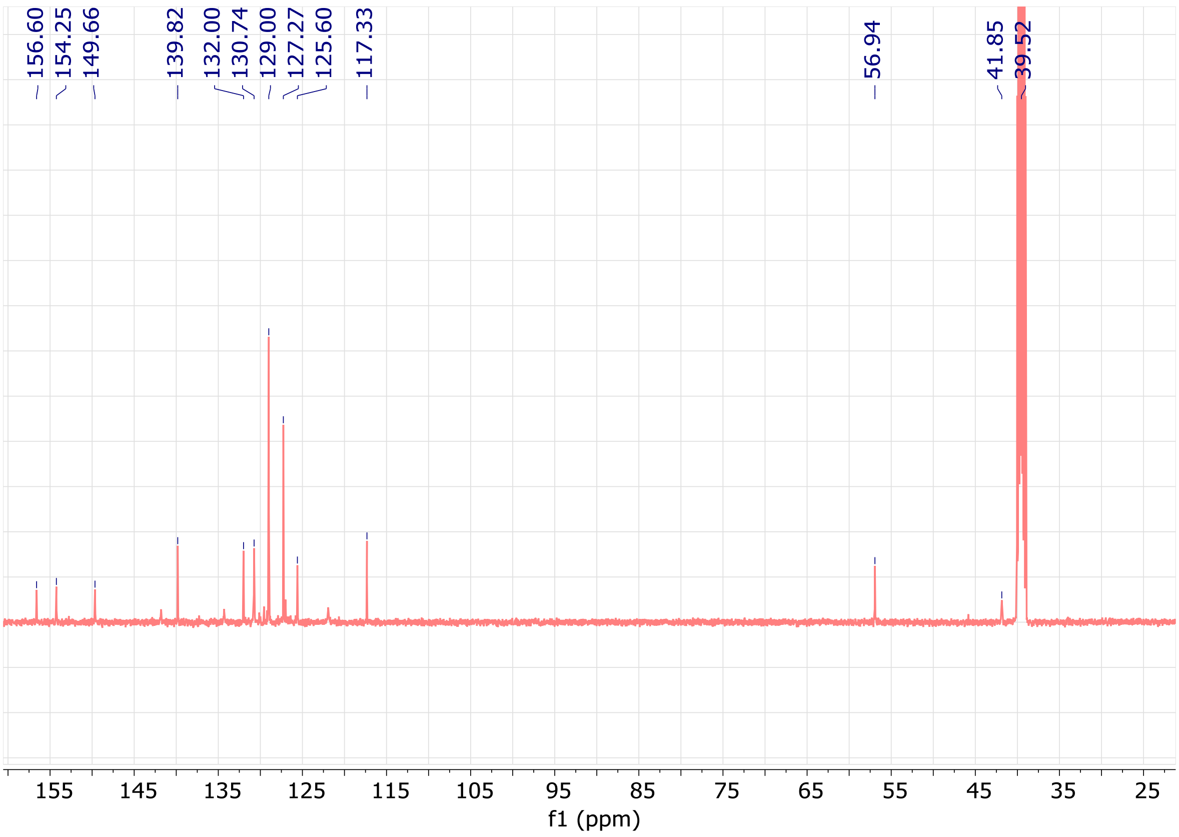

156.60
154.25
149.66
139.82
132.00
130.74
129.00
127.27
125.60
117.33
56.94
41.85
39.52
f1 (ppm)

Fg-12

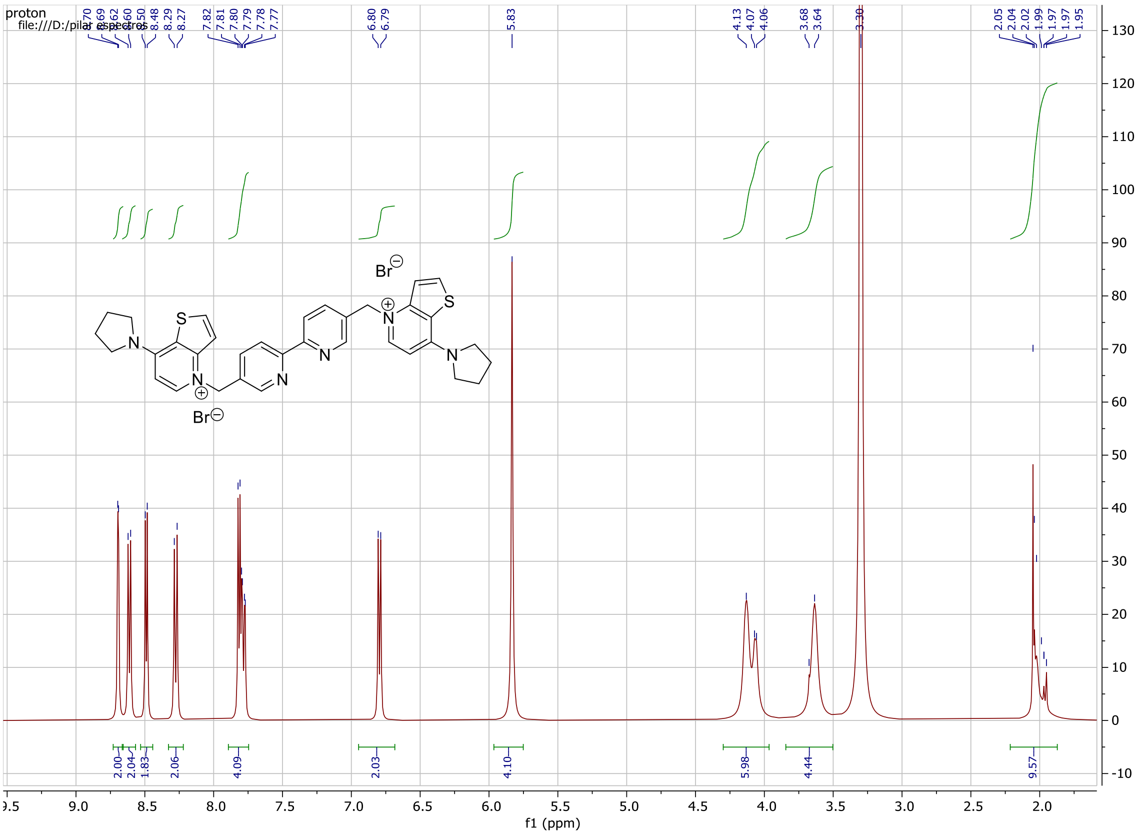
proton
file:///D:/pilar espectros
8.70 8.69 8.62 8.60 8.50 8.48 8.29 8.27 7.82 7.81 7.80 7.79 7.78 7.77 6.80 6.79 5.83 4.13 4.07 4.06 3.68 3.64 3.30 2.05 2.04 2.02 1.99 1.97 1.97 1.95
Br⊖
S
N
N⊕
N
N
2.00 2.04 1.83 2.06 4.09 2.03 4.10 5.98 4.44 9.57
f1 (ppm)

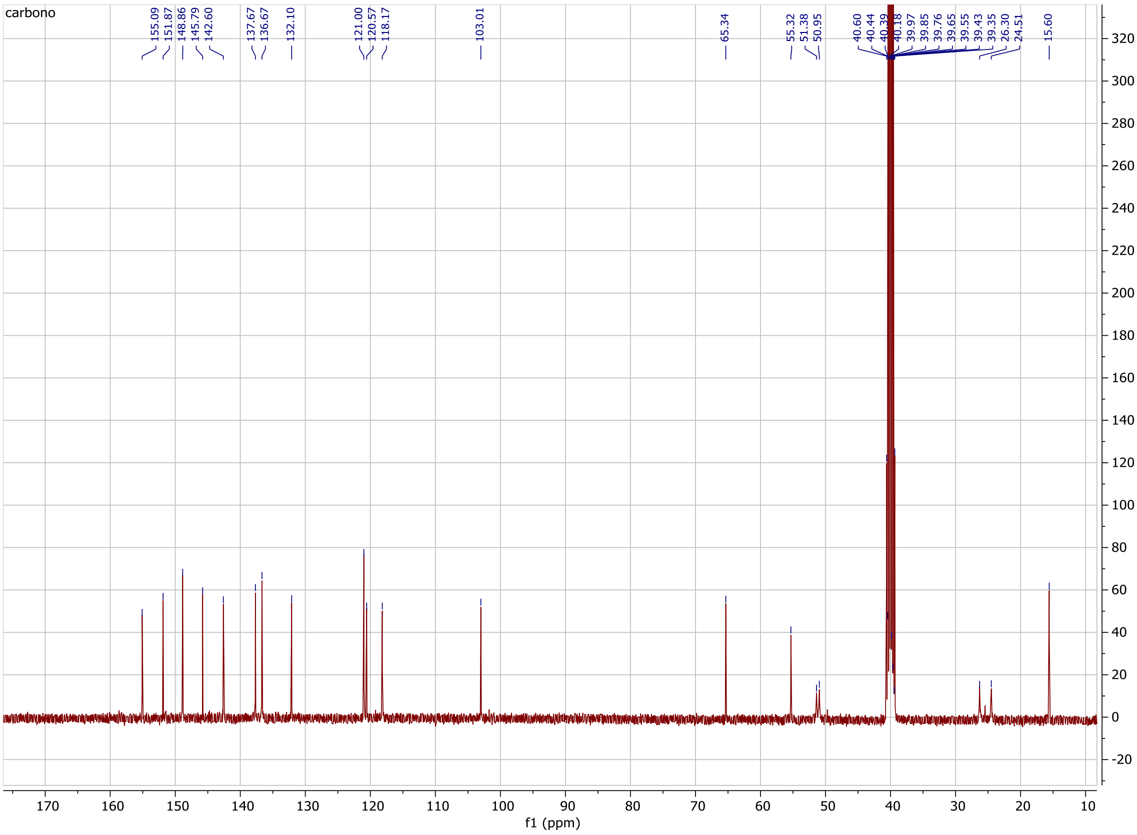
carbono
155.09 151.87 148.86 145.79 142.60 137.67 136.67 132.10 121.00 120.57 118.17 103.01 65.34 55.32 51.38 50.95 40.60 40.44 40.39 40.18 39.97 39.85 39.76 39.65 39.55 39.43 39.35 26.30 24.51 15.60
f1 (ppm)

Fg-17

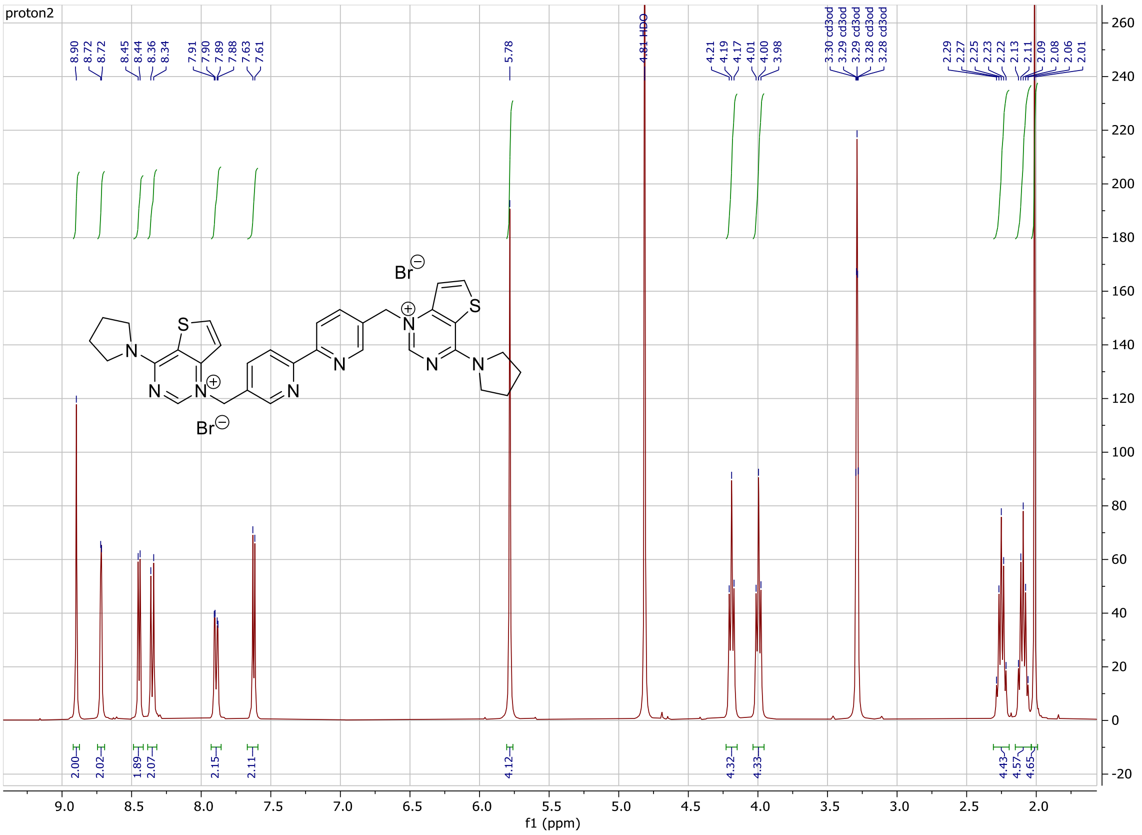
proton2
8.90
8.72
8.72
8.45
8.44
8.36
8.34
7.91
7.90
7.89
7.88
7.63
7.61
5.78
4.81 HDO
4.21
4.19
4.17
4.01
4.00
3.98
3.30 cd3od
3.29 cd3od
3.29 cd3od
3.28 cd3od
3.28 cd3od
2.29
2.27
2.25
2.23
2.22
2.13
2.11
2.09
2.08
2.06
2.01
Br
S
N
N
N
N
N
N
N
S
N
N
Br
2.00
2.02
1.89
2.07
2.15
2.11
4.12
4.32
4.33
4.43
4.57
4.65
f1 (ppm)

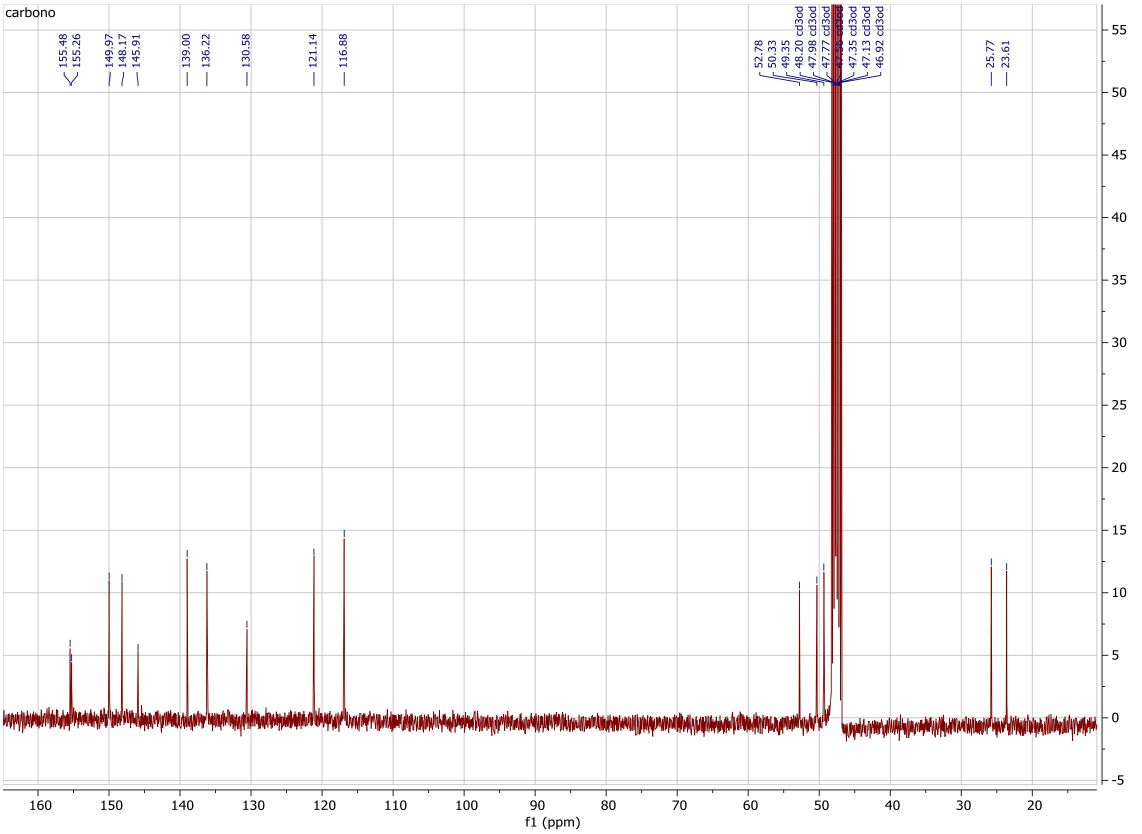
carbono
155.48
155.26
149.97
148.17
145.91
139.00
136.22
130.58
121.14
116.88
52.78
50.33
49.35
48.20 cd3od
47.98 cd3od
47.77 cd3od
47.35 cd3od
47.13 cd3od
46.92 cd3od
25.77
23.61
f1 (ppm)

Fg-13

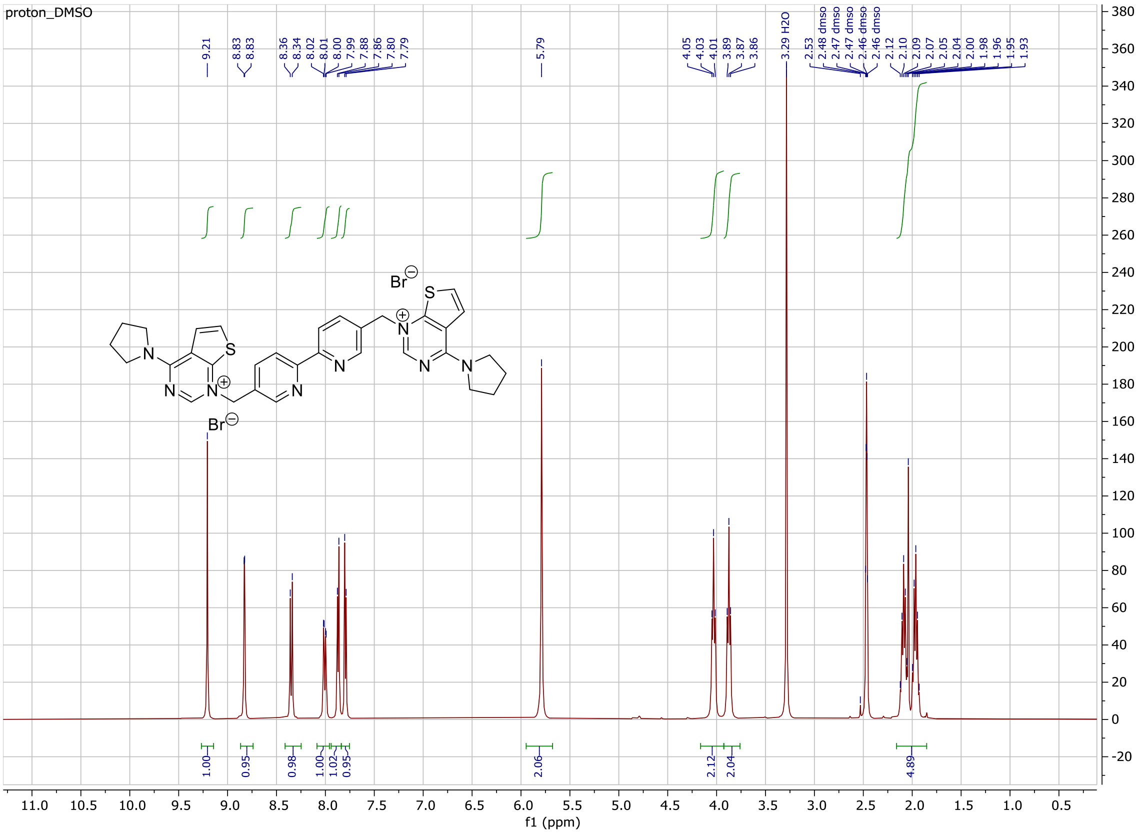
proton_DMSO
9.21
8.83
8.83
8.36
8.34
8.02
8.01
8.00
7.99
7.88
7.86
7.80
7.79
5.79
4.05
4.03
4.01
3.89
3.87
3.86
3.29 H2O
2.53
2.48 dmso
2.47 dmso
2.47 dmso
2.46 dmso
2.46 dmso
2.12
2.10
2.09
2.07
2.05
2.04
2.00
1.98
1.96
1.95
1.93
Br
S
N
N
N
N
N
S
N
N
N
N
Br
1.00
0.95
0.98
1.00
1.02
0.95
2.06
2.12
2.04
4.89
f1 (ppm)

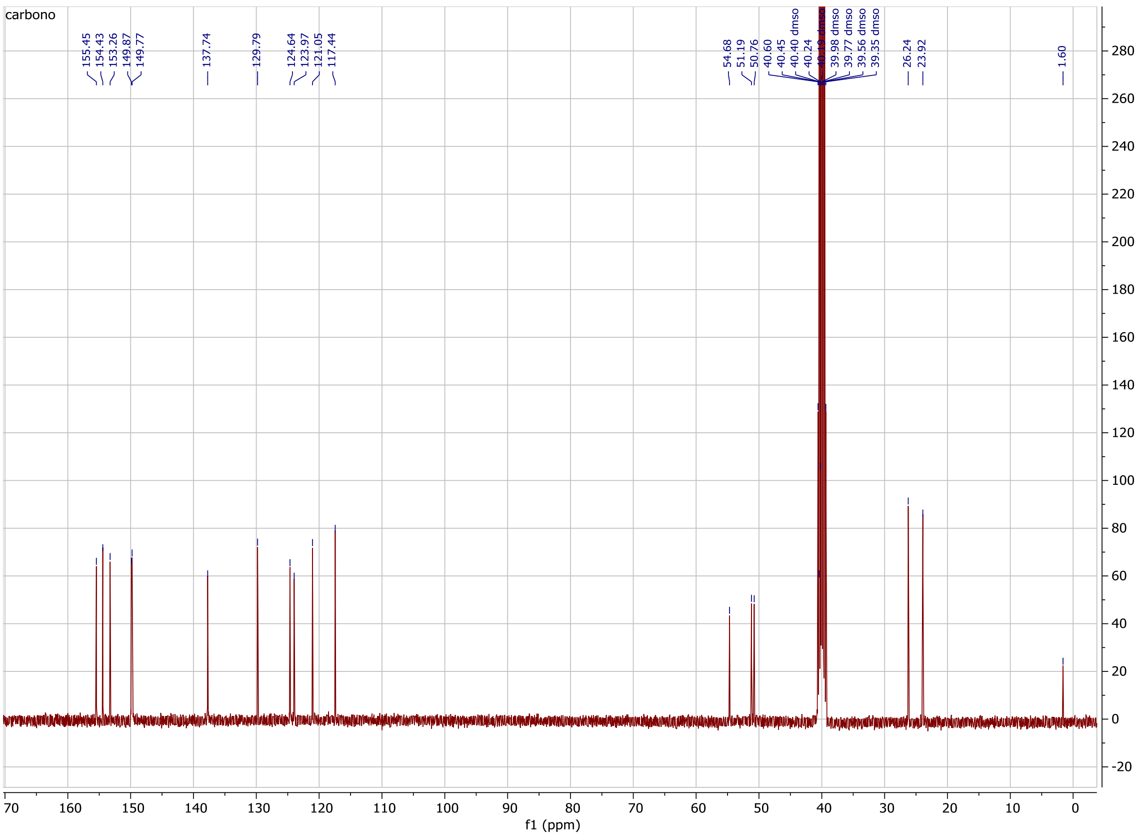
carbono
155.45
154.43
153.26
149.87
149.77
137.74
129.79
124.64
123.97
121.05
117.44
54.68
51.19
50.76
40.60
40.45
40.40 dmso
40.24
39.98 dmso
39.77 dmso
39.56 dmso
39.35 dmso
26.24
23.92
1.60
f1 (ppm)

Fa-27

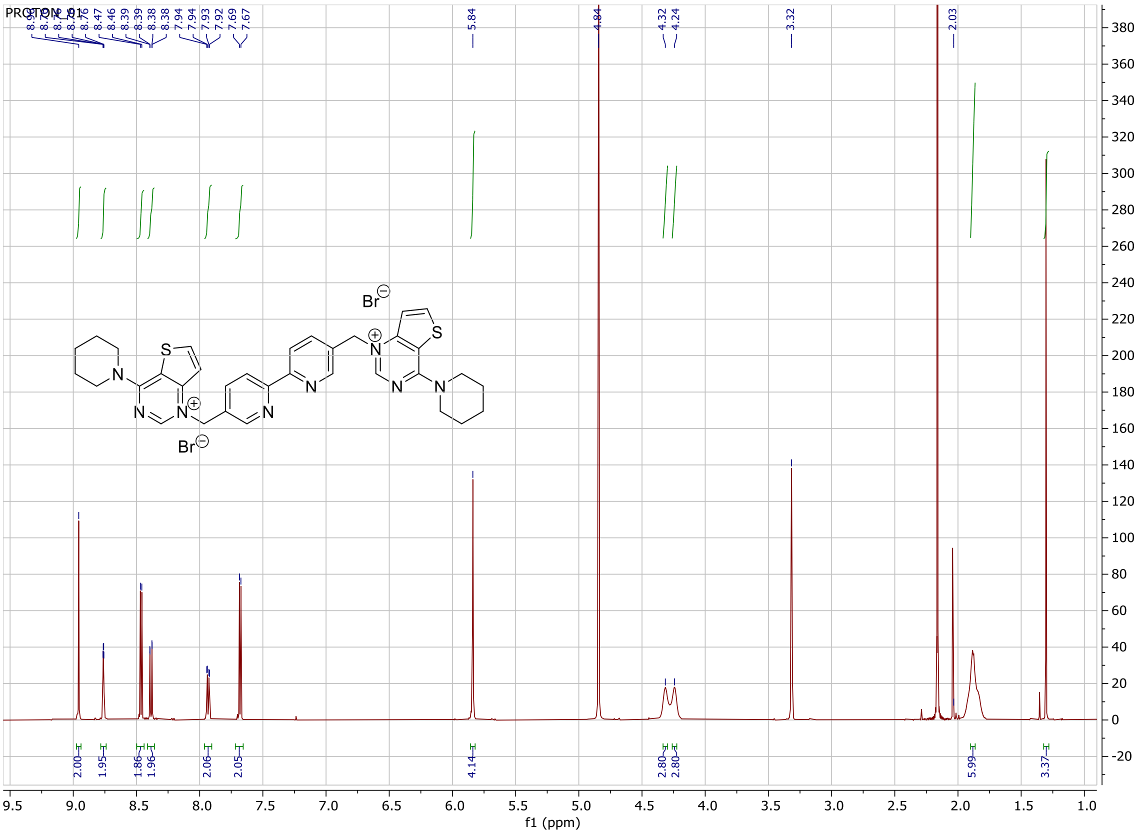
PROTON_01
8.47 8.46 8.39 8.39 8.38 8.38 7.94 7.94 7.93 7.92 7.69 7.67
5.84
4.84
4.32 4.24
3.32
2.03
Br⊖
S
N
N
N⊕
Br⊖
N
N
N⊕
S
N
N
N
2.00 1.95 1.86 1.96 2.06 2.05 4.14 2.80 2.80 5.99 3.37
f1 (ppm)

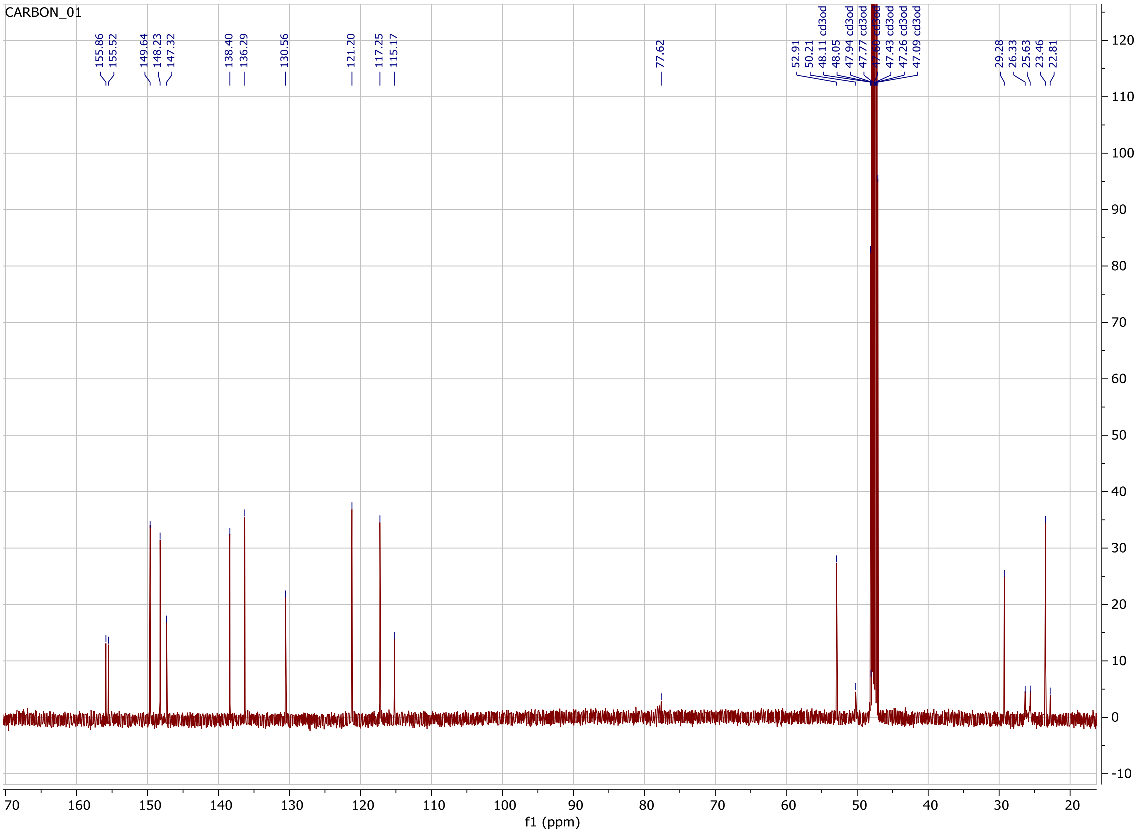
CARBON_01
155.86 155.52 149.64 148.23 147.32 138.40 136.29 130.56 121.20 117.25 115.17 77.62
52.91 50.21 48.11 cd3od 48.05 47.94 cd3od 47.77 cd3od 47.43 cd3od 47.26 cd3od 47.09 cd3od
29.28 26.33 25.63 23.46 22.81
f1 (ppm)

Fg-32

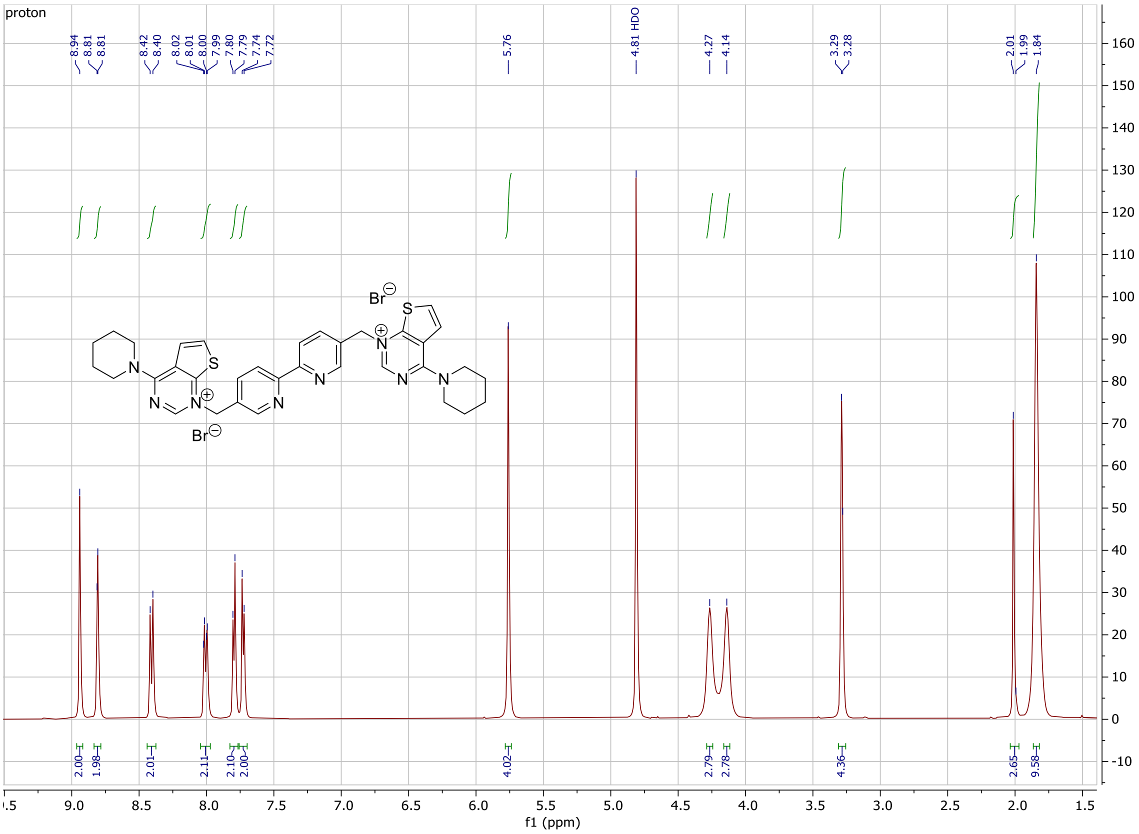
proton
8.94
8.81
8.81
8.42
8.40
8.02
8.01
8.00
7.99
7.80
7.79
7.74
7.72
5.76
4.81 HDO
4.27
4.14
3.29
3.28
2.01
1.99
1.84
2.00
1.98
2.01
2.11
2.10
2.00
4.02
2.79
2.78
4.36
2.65
9.58
Br
S
N
N
N
N
N
N
S
N
N
Br
f1 (ppm)

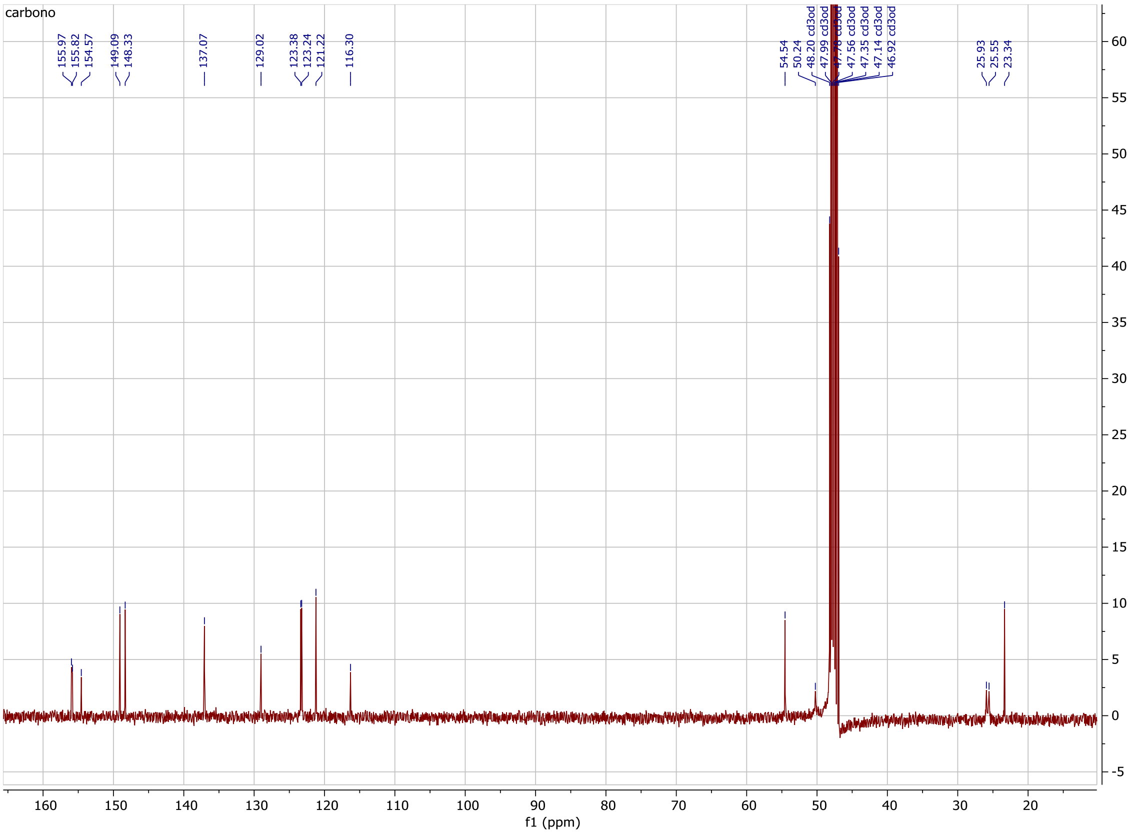
carbono
155.97
155.82
154.57
149.09
148.33
137.07
129.02
123.38
123.24
121.22
116.30
54.54
50.24
48.20 cd3od
47.99 cd3od
47.78 cd3od
47.56 cd3od
47.35 cd3od
47.14 cd3od
46.92 cd3od
25.93
25.55
23.34
f1 (ppm)

Fa-26

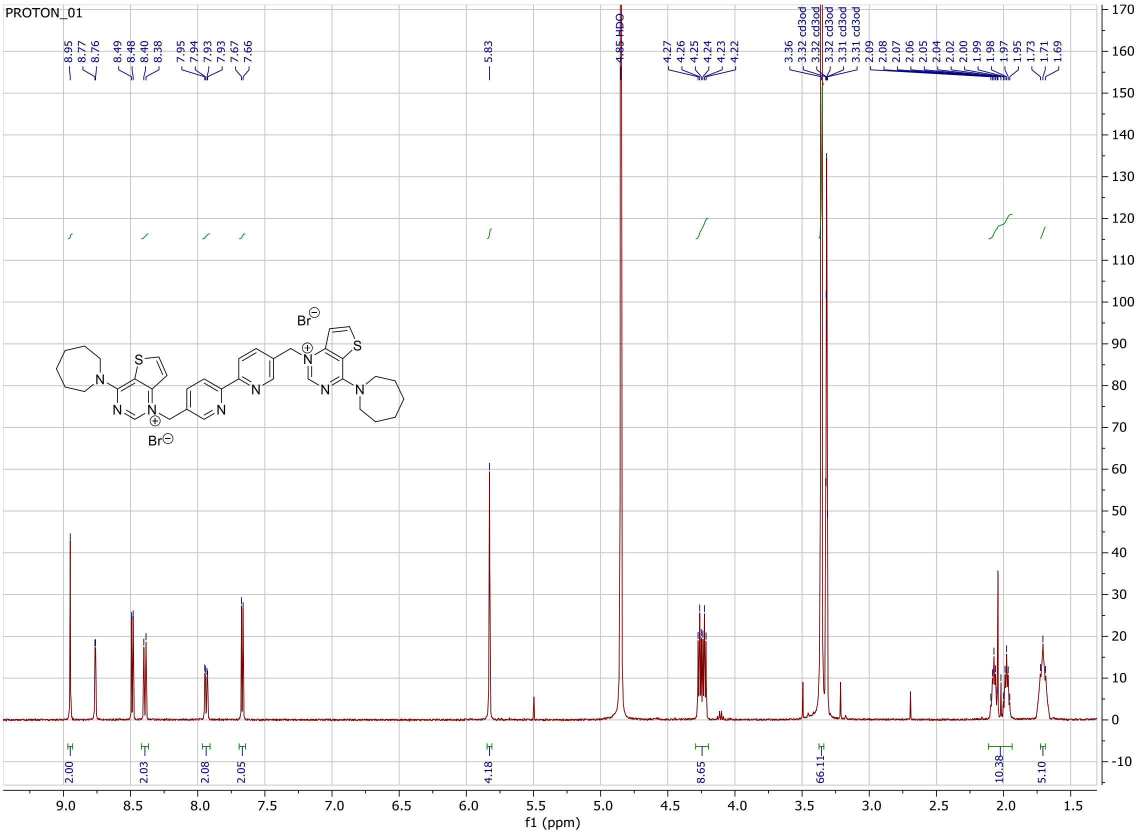
PROTON_01
8.95
8.77
8.76
8.49
8.48
8.40
8.38
7.95
7.94
7.93
7.93
7.67
7.66
5.83
4.85 HDO
4.27
4.26
4.25
4.24
4.23
4.22
3.36
3.32 cd3od
3.32 cd3od
3.32 cd3od
3.31 cd3od
3.31 cd3od
2.09
2.08
2.07
2.06
2.05
2.04
2.02
2.00
1.99
1.98
1.97
1.95
1.73
1.71
1.69
Br⊖
S
N
N
N
⊕
Br⊖
2.00
2.03
2.08
2.05
4.18
8.65
66.11
10.38
5.10
f1 (ppm)

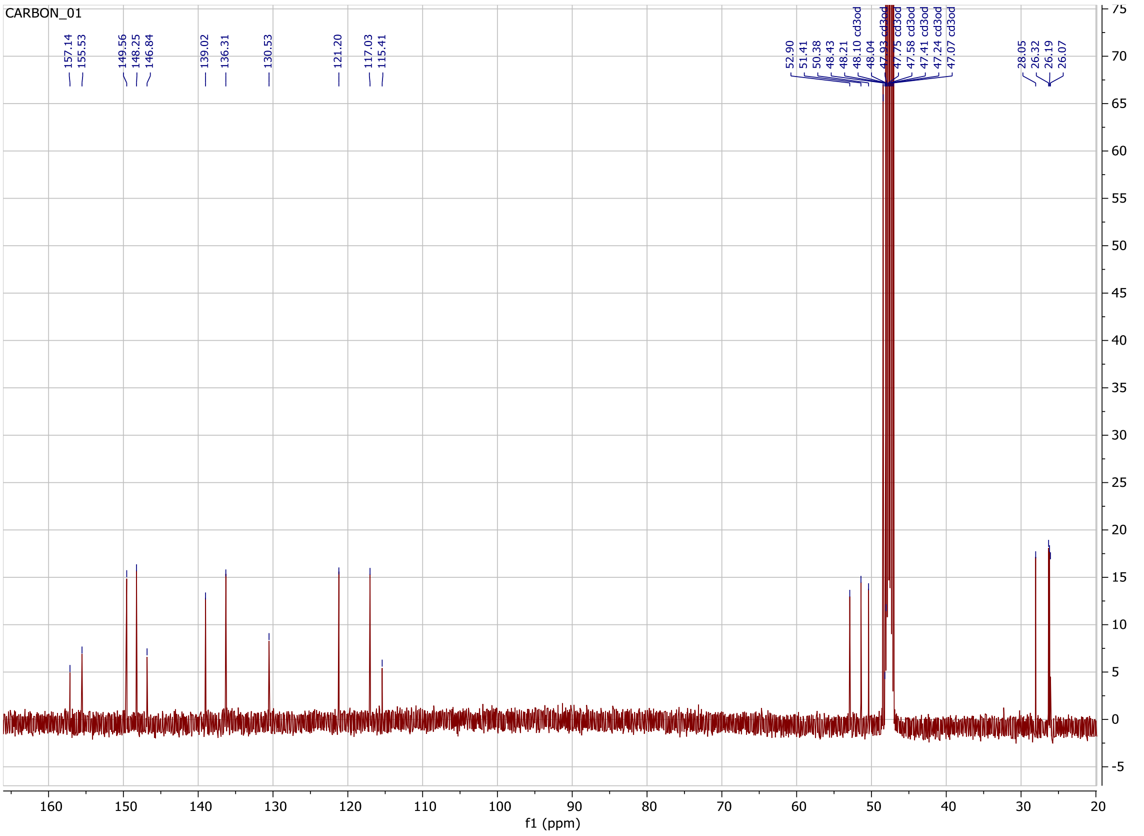
CARBON_01
157.14
155.53
149.56
148.25
146.84
139.02
136.31
130.53
121.20
117.03
115.41
52.90
51.41
50.38
48.43
48.21
48.10 cd3od
48.04
47.93 cd3od
47.75 cd3od
47.58 cd3od
47.41 cd3od
47.24 cd3od
47.07 cd3od
28.05
26.32
26.19
26.07
f1 (ppm)

Fg-20

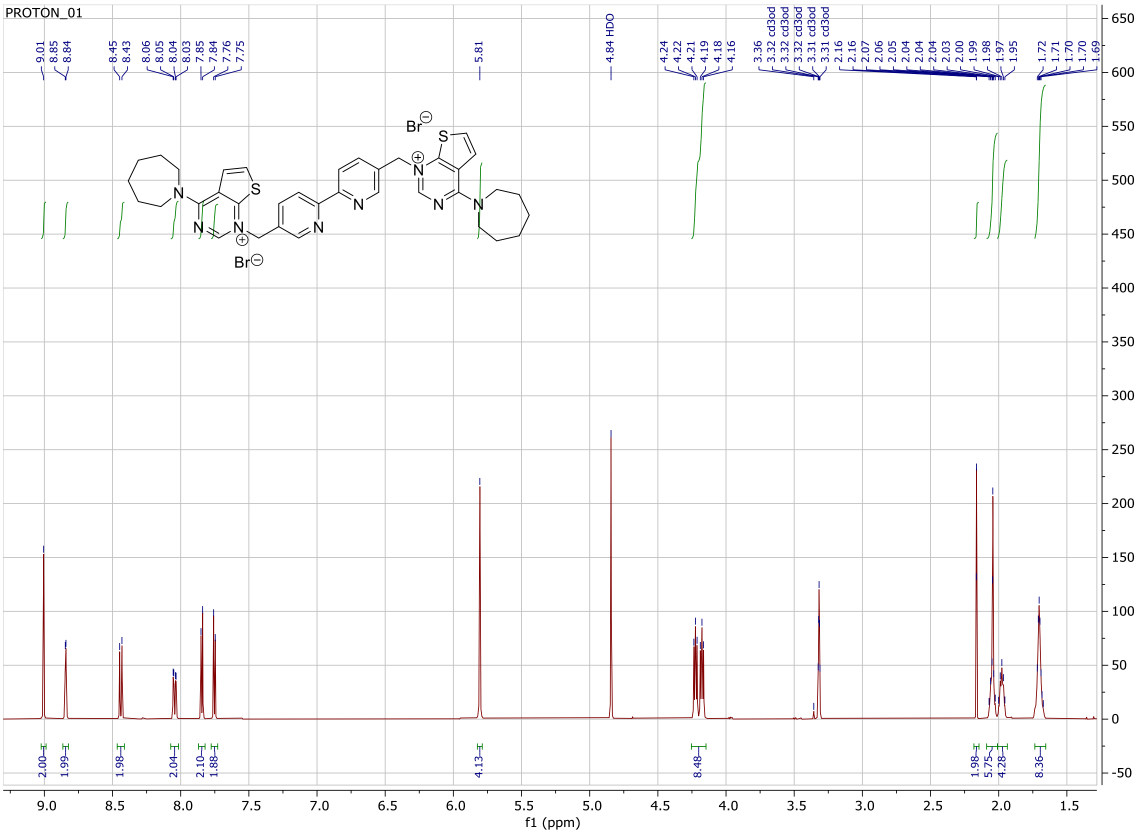
PROTON_01
9.01
8.85
8.84
8.45
8.43
8.06
8.05
8.04
8.03
7.85
7.84
7.76
7.75
5.81
4.84 HDO
4.24
4.22
4.21
4.19
4.18
4.16
3.36
3.32 cd3od
3.32 cd3od
3.32 cd3od
3.31 cd3od
3.31 cd3od
2.16
2.16
2.07
2.06
2.05
2.04
2.04
2.03
2.00
1.99
1.98
1.97
1.95
1.72
1.71
1.70
1.70
1.69
Br⊖
S
N
N
N
N
N
S
N
N
N
Br⊖
2.00
1.99
1.98
2.04
2.10
1.88
4.13
8.48
1.98
5.75
4.28
8.36
f1 (ppm)

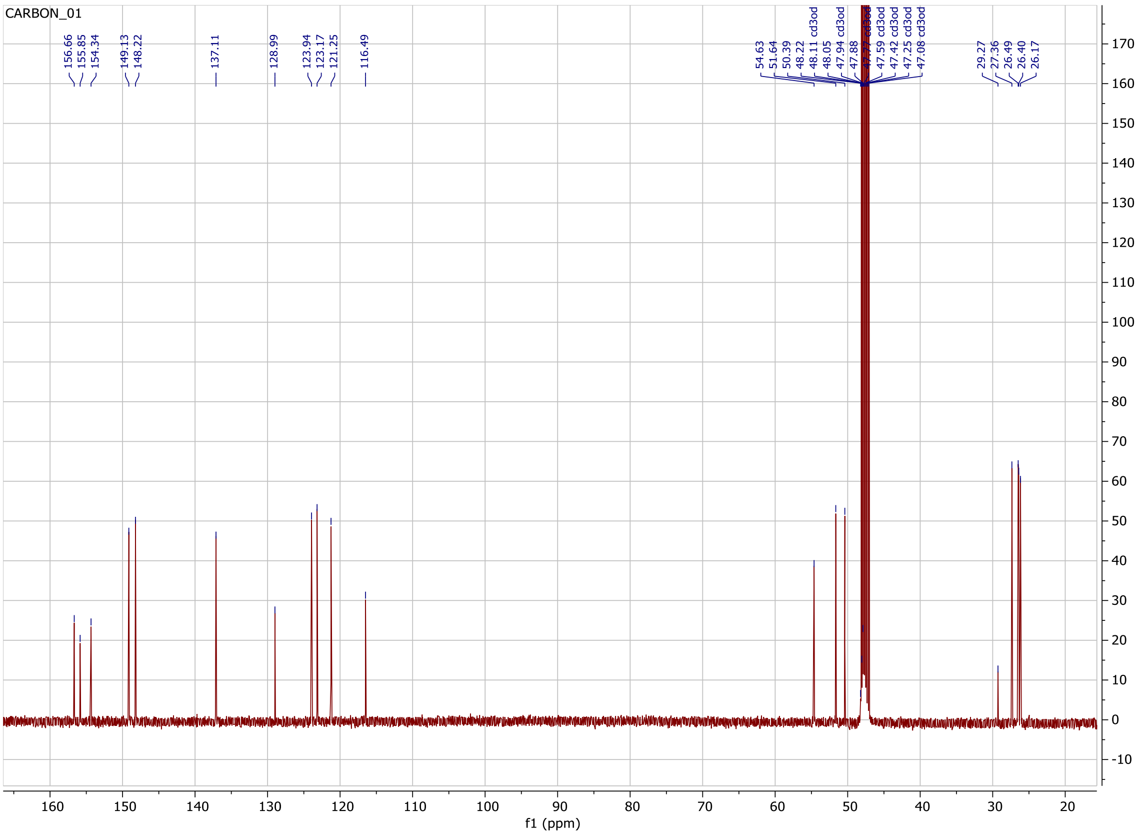
CARBON_01
156.66
155.85
154.34
149.13
148.22
137.11
128.99
123.94
123.17
121.25
116.49
54.63
51.64
50.39
48.22
48.11 cd3od
48.05
47.94 cd3od
47.88
47.59 cd3od
47.42 cd3od
47.25 cd3od
47.08 cd3od
29.27
27.36
26.49
26.40
26.17
f1 (ppm)

Fg-11

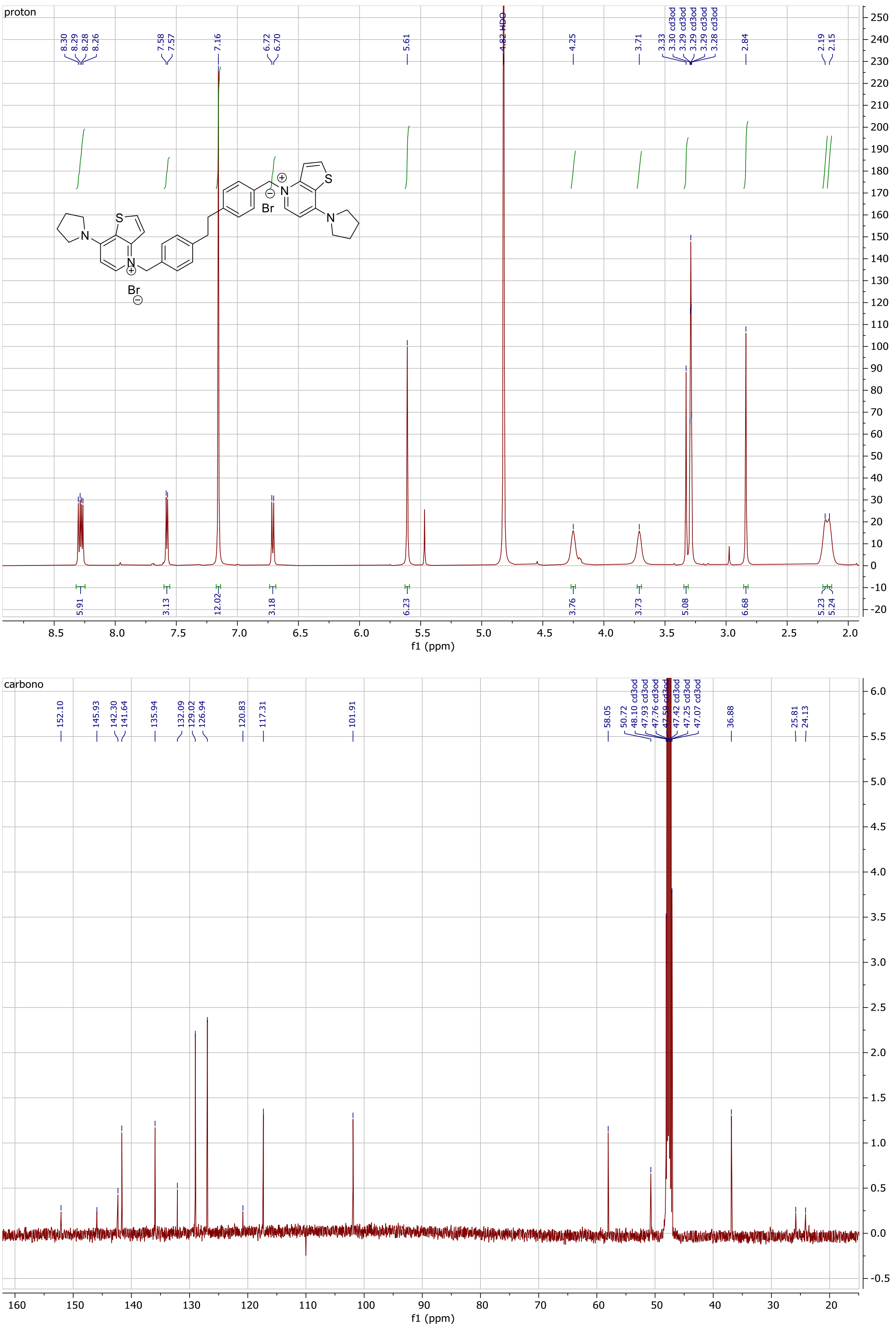
proton
8.30
8.29
8.28
8.26
7.58
7.57
7.16
6.72
6.70
5.61
4.82 HDO
4.25
3.71
3.33
3.30 cd3od
3.29 cd3od
3.29 cd3od
3.29 cd3od
3.28 cd3od
2.84
2.19
2.15
5.91
3.13
12.02
3.18
6.23
3.76
3.73
5.08
6.68
5.23
5.24
f1 (ppm)
carbono
152.10
145.93
142.30
141.64
135.94
132.09
129.02
126.94
120.83
117.31
101.91
58.05
50.72
48.10 cd3od
47.93 cd3od
47.76 cd3od
47.59 cd3od
47.42 cd3od
47.25 cd3od
47.07 cd3od
36.88
25.81
24.13
f1 (ppm)

Fg-16

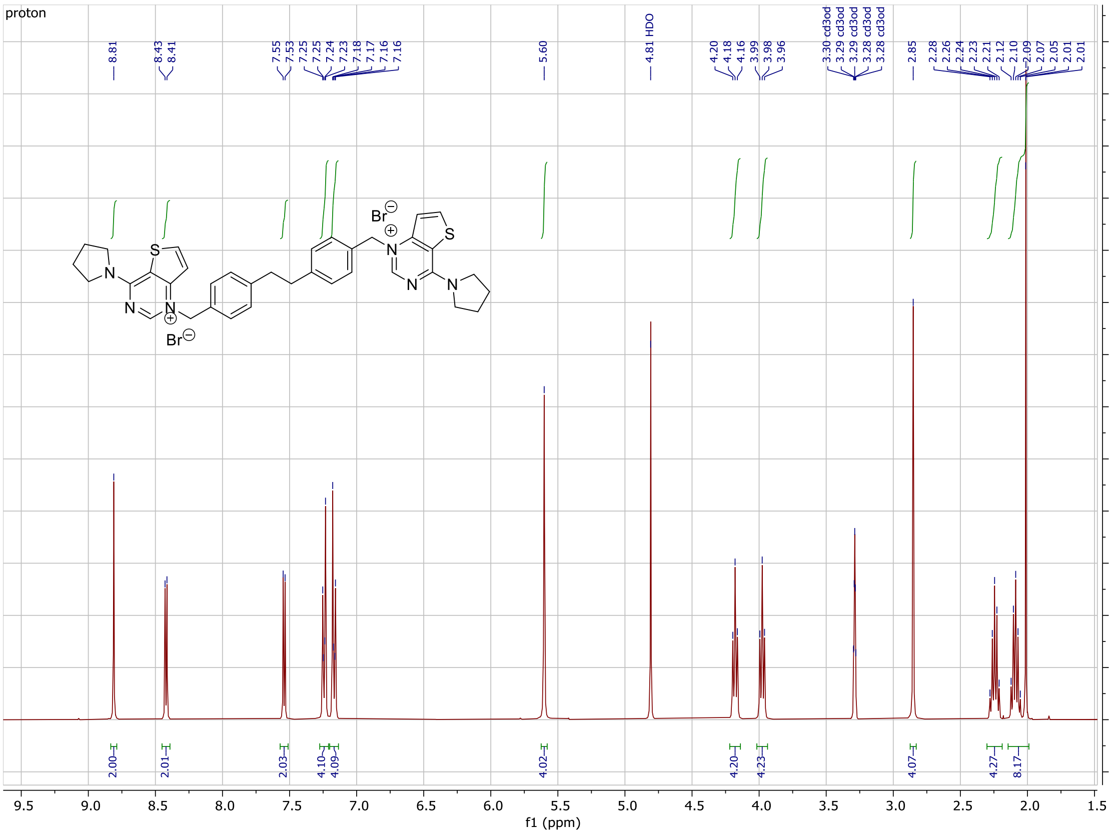
proton
8.81
8.43
8.41
7.55
7.53
7.25
7.25
7.24
7.23
7.18
7.17
7.16
7.16
5.60
4.81 HDO
4.20
4.18
4.16
3.99
3.98
3.96
3.30 cd3od
3.29 cd3od
3.29 cd3od
3.28 cd3od
3.28 cd3od
2.85
2.28
2.26
2.24
2.23
2.21
2.12
2.10
2.09
2.07
2.05
2.01
2.01
2.00
2.01
2.03
4.10
4.09
4.02
4.20
4.23
4.07
4.27
8.17
f1 (ppm)

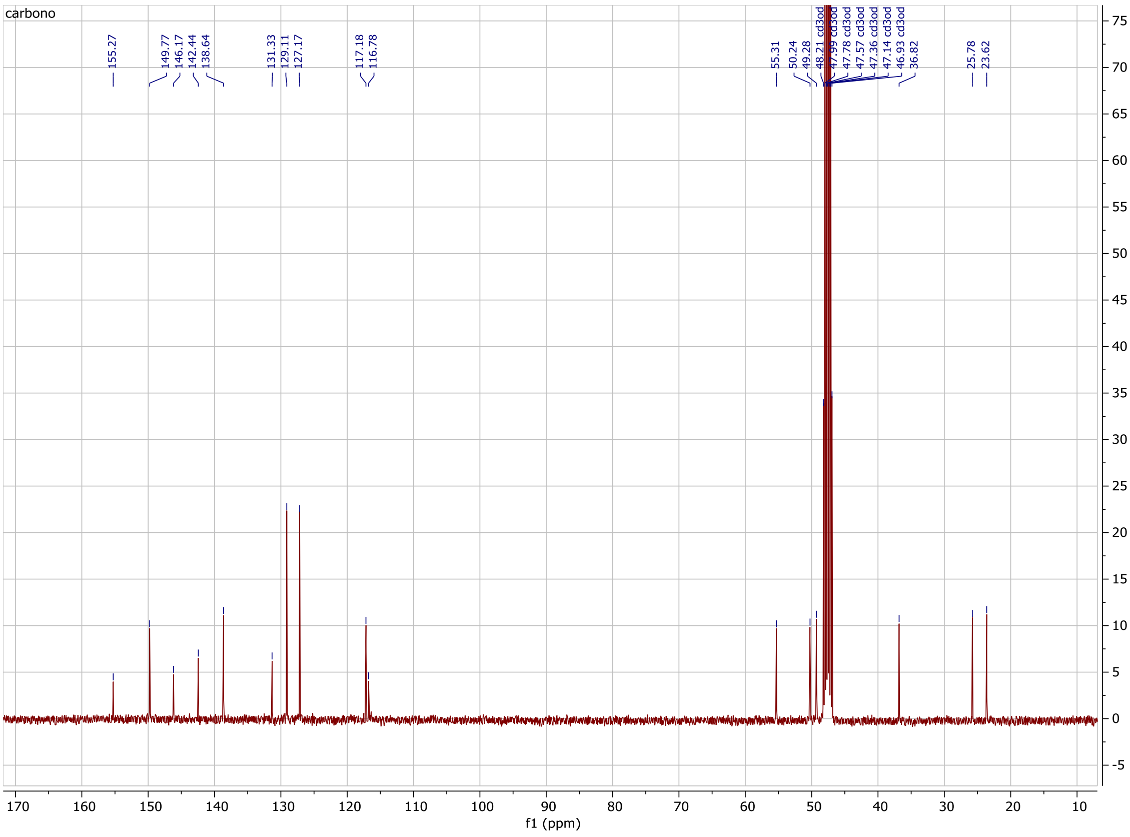
carbono
155.27
149.77
146.17
142.44
138.64
131.33
129.11
127.17
117.18
116.78
55.31
50.24
49.28
48.21 cd3od
47.99 cd3od
47.78 cd3od
47.57 cd3od
47.36 cd3od
47.14 cd3od
46.93 cd3od
36.82
25.78
23.62
f1 (ppm)

Fg-15

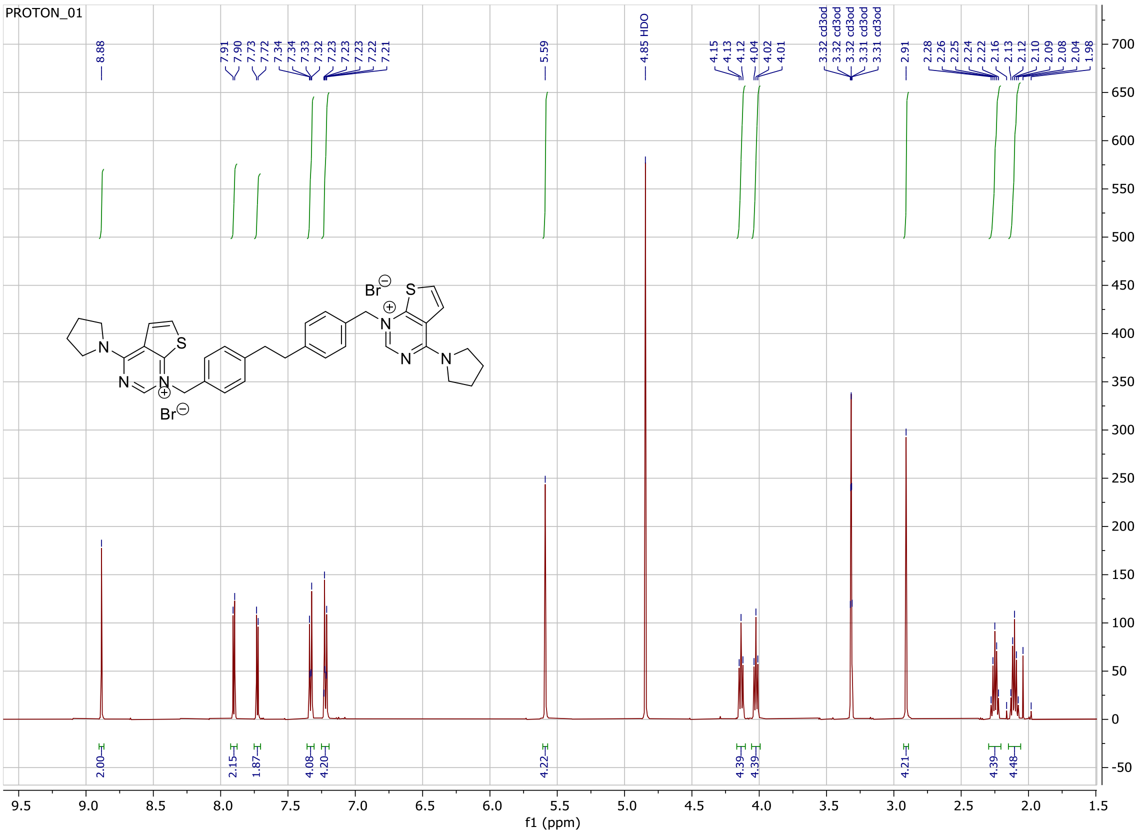
PROTON_01
8.88
7.91
7.90
7.73
7.72
7.34
7.33
7.32
7.23
7.23
7.23
7.22
7.21
5.59
4.85 HDO
4.15
4.13
4.12
4.04
4.02
4.01
3.32 cd3od
3.32 cd3od
3.32 cd3od
3.31 cd3od
3.31 cd3od
2.91
2.28
2.26
2.25
2.24
2.22
2.16
2.13
2.12
2.10
2.09
2.08
2.04
1.98
2.00
2.15
1.87
4.08
4.20
4.22
4.39
4.39
4.21
4.39
4.48
Br
S
N
N
N
N
S
N
N
Br
f1 (ppm)

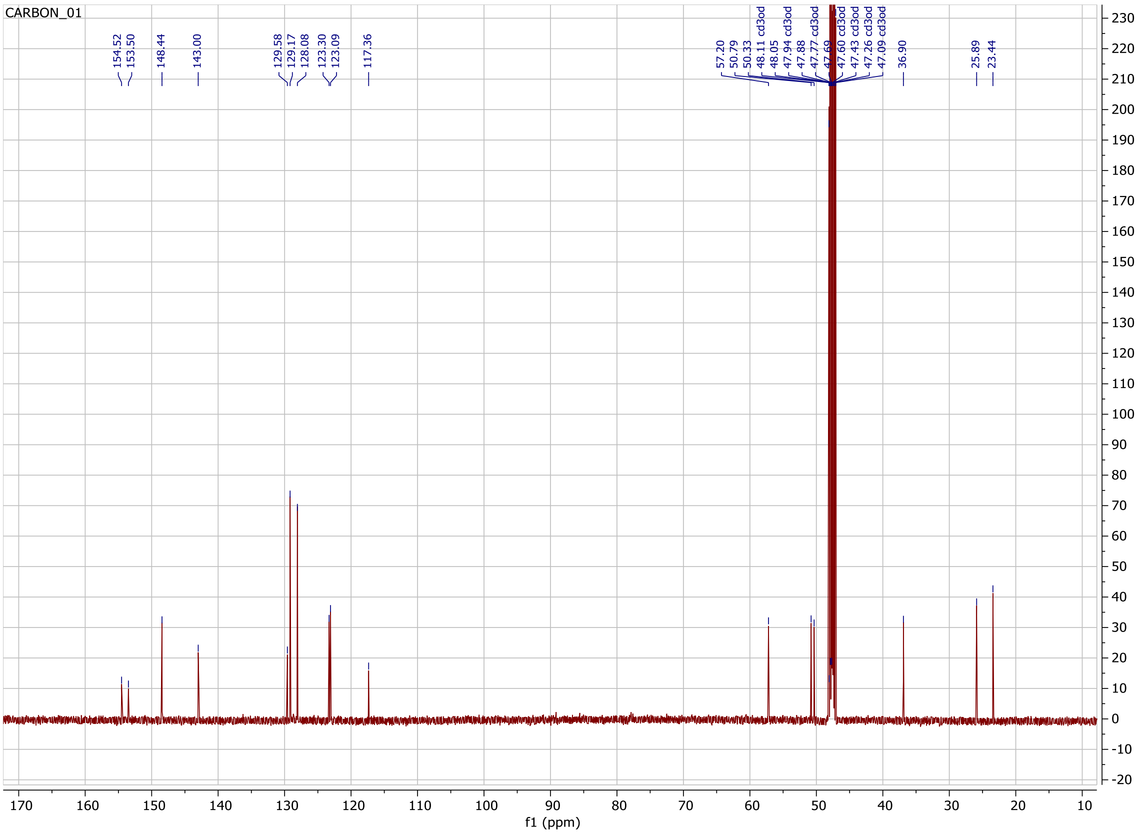
CARBON_01
154.52
153.50
148.44
143.00
129.58
129.17
128.08
123.30
123.09
117.36
57.20
50.79
50.33
48.11 cd3od
48.05
47.94 cd3od
47.88
47.77 cd3od
47.69
47.60 cd3od
47.43 cd3od
47.26 cd3od
47.09 cd3od
36.90
25.89
23.44
f1 (ppm)

Fa-25

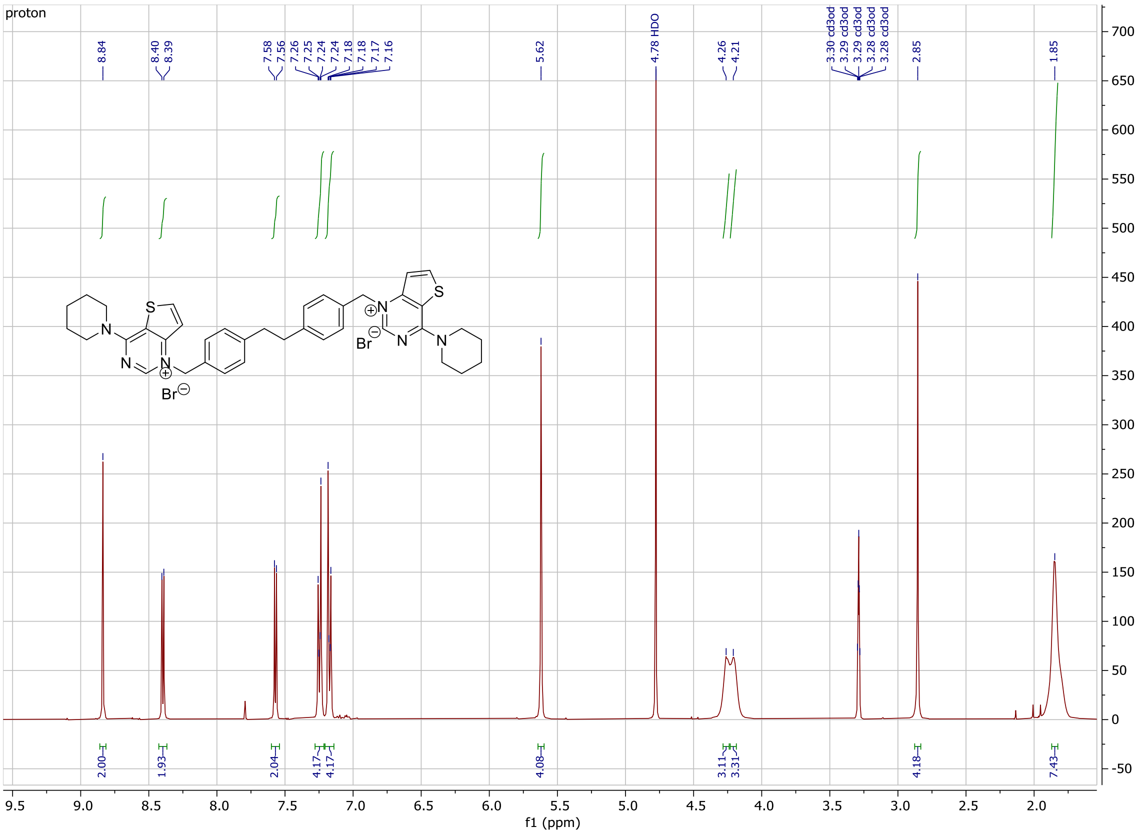
proton
8.84
8.40
8.39
7.58
7.56
7.26
7.25
7.24
7.24
7.18
7.17
7.16
5.62
4.78 HDO
4.26
4.21
3.30 cd3od
3.29 cd3od
3.29 cd3od
3.28 cd3od
3.28 cd3od
2.85
1.85
2.00
1.93
2.04
4.17
4.17
4.08
3.11
3.31
4.18
7.43
f1 (ppm)

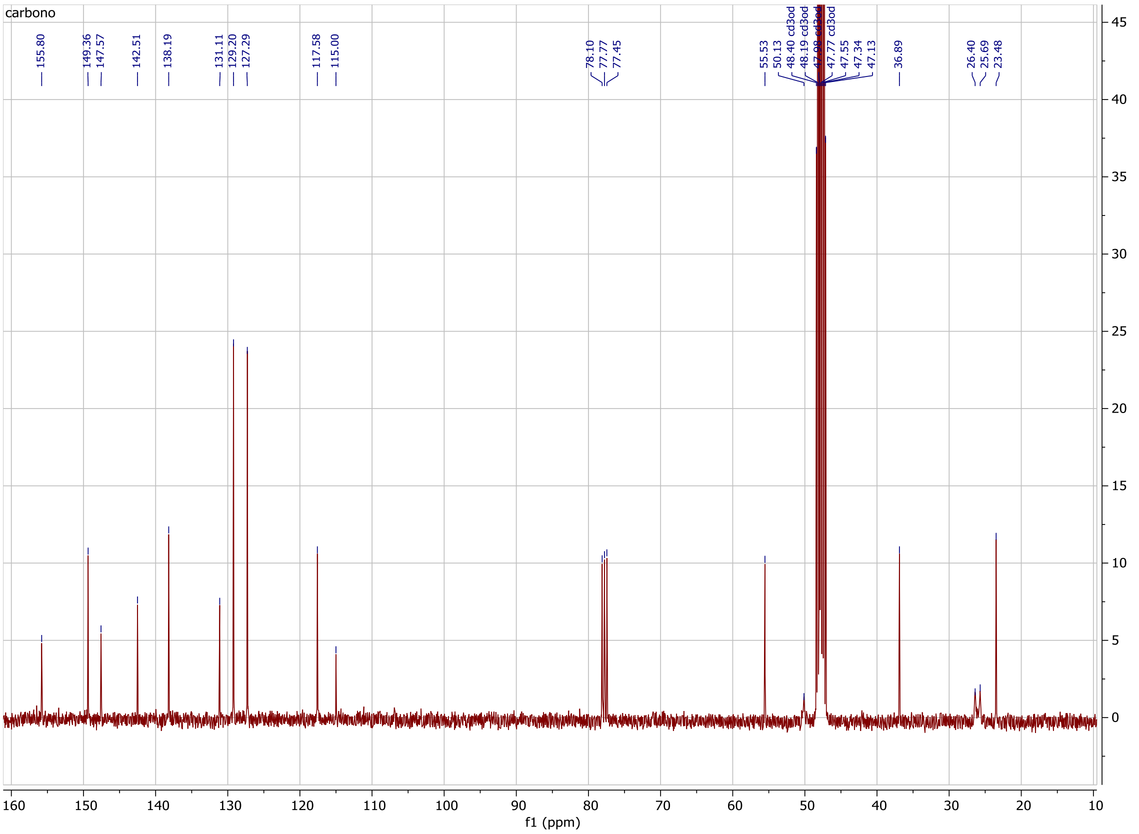
carbono
155.80
149.36
147.57
142.51
138.19
131.11
129.20
127.29
117.58
115.00
78.10
77.77
77.45
55.53
50.13
48.40 cd3od
48.19 cd3od
47.98 cd3od
47.77 cd3od
47.55
47.34
47.13
36.89
26.40
25.69
23.48
f1 (ppm)

Fg-31

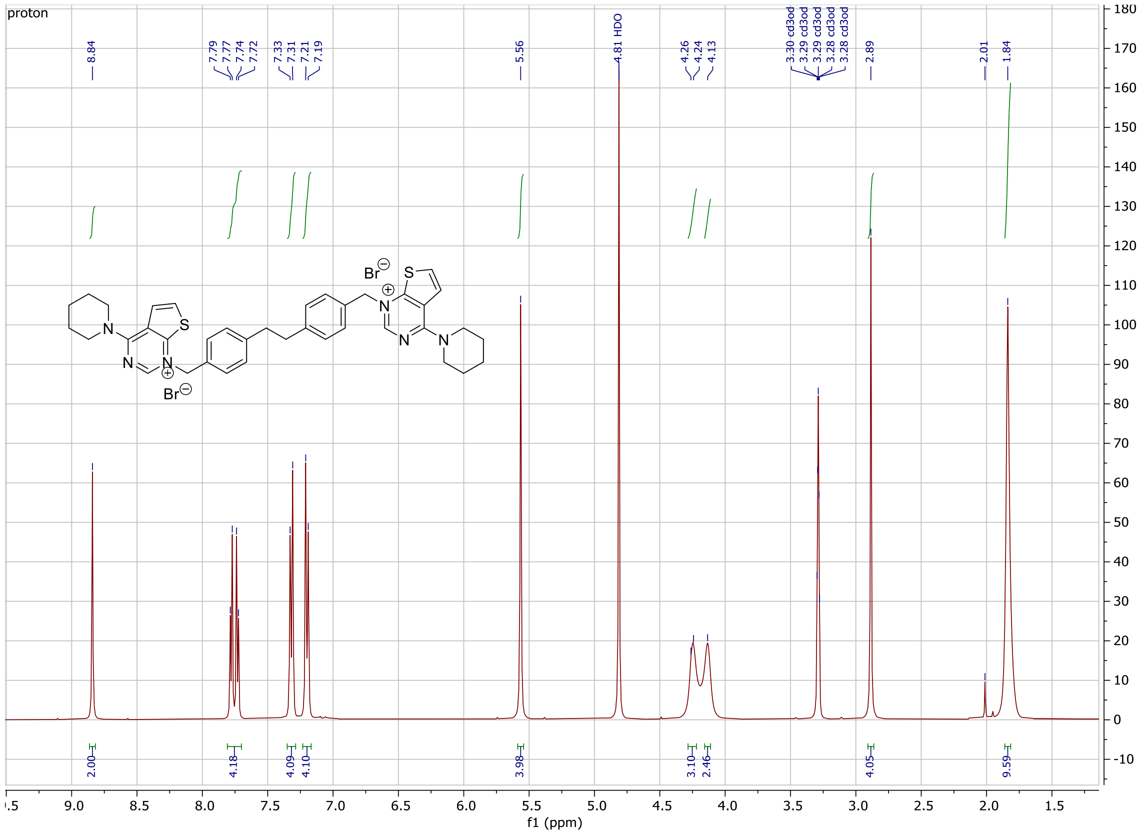
proton
8.84
7.79
7.77
7.74
7.72
7.33
7.31
7.21
7.19
5.56
4.81 HDO
4.26
4.24
4.13
3.30 cd3od
3.29 cd3od
3.29 cd3od
3.28 cd3od
3.28 cd3od
2.89
2.01
1.84
2.00
4.18
4.09
4.10
3.98
3.10
2.46
4.05
9.59
Br
N
S
f1 (ppm)

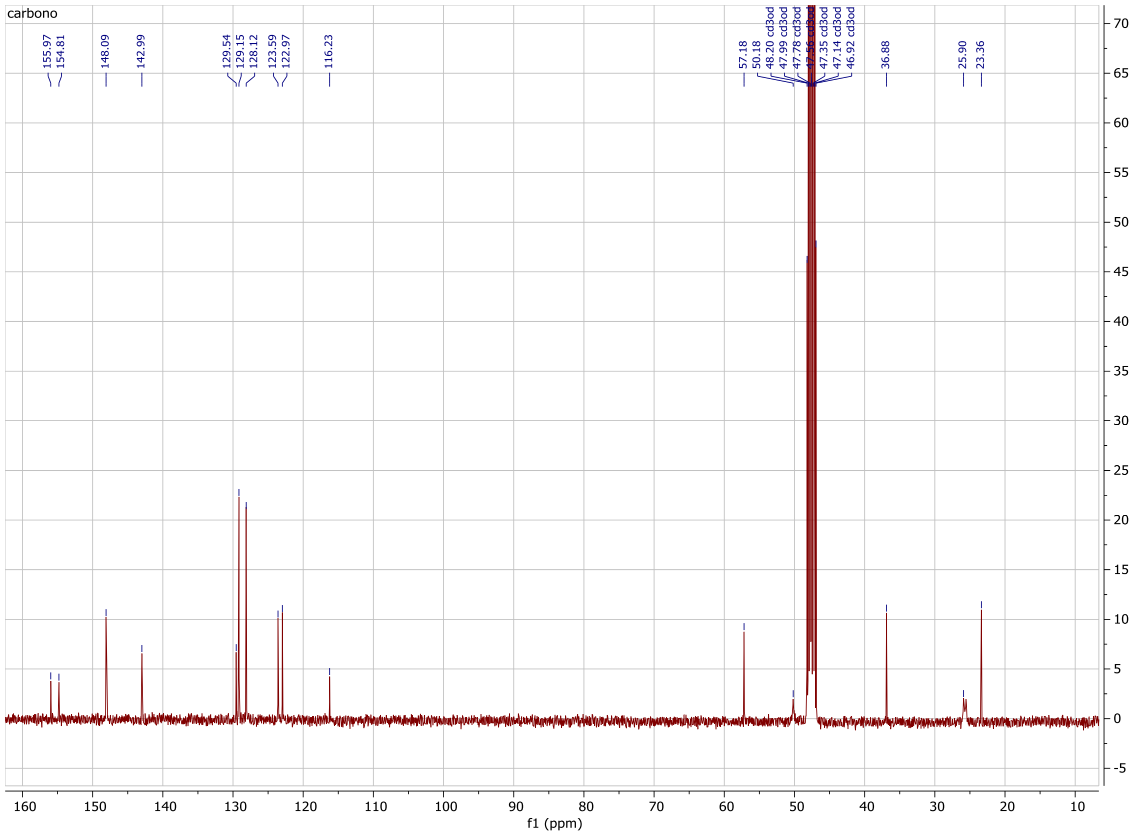
carbono
155.97
154.81
148.09
142.99
129.54
129.15
128.12
123.59
122.97
116.23
57.18
50.18
48.20 cd3od
47.99 cd3od
47.78 cd3od
47.35 cd3od
47.14 cd3od
46.92 cd3od
36.88
25.90
23.36
f1 (ppm)

Fa-23

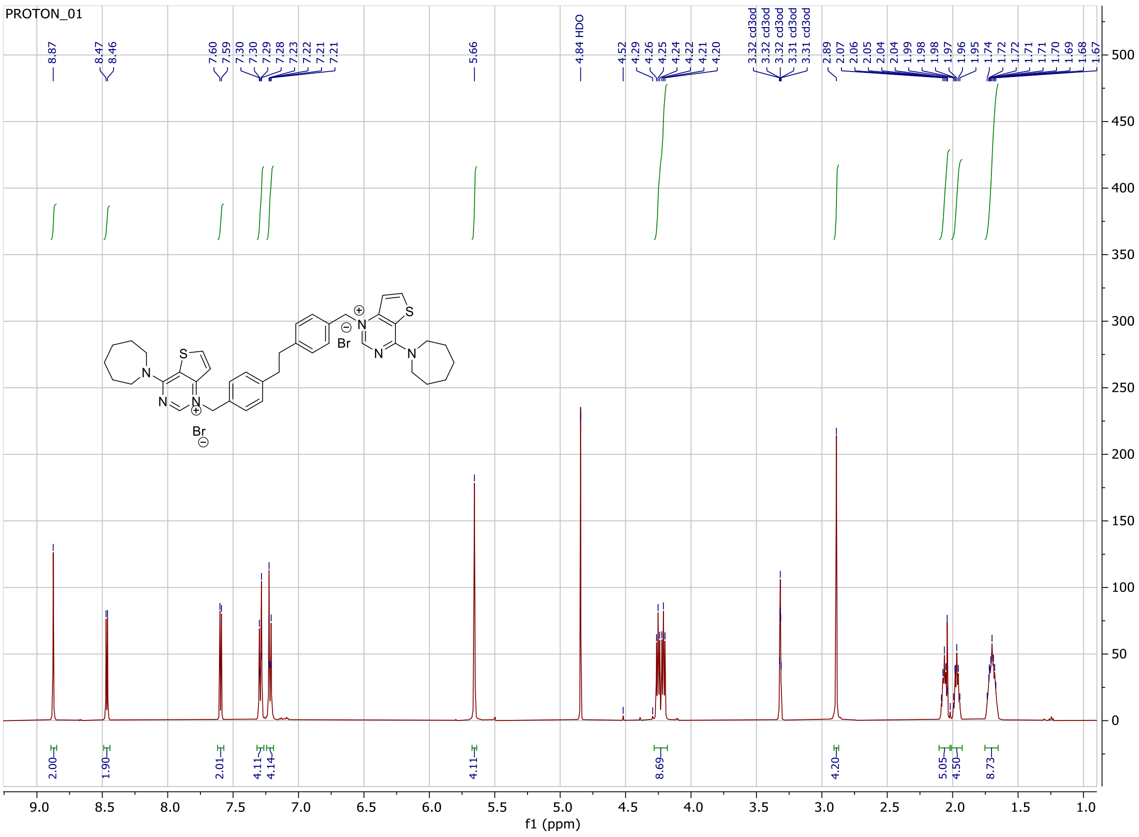
PROTON_01
8.87
8.47
8.46
7.60
7.59
7.30
7.30
7.29
7.28
7.23
7.22
7.21
7.21
5.66
4.84 HDO
4.52
4.29
4.26
4.25
4.24
4.22
4.21
4.20
3.32 cd3od
3.32 cd3od
3.32 cd3od
3.31 cd3od
3.31 cd3od
2.89
2.07
2.06
2.05
2.04
2.04
1.99
1.98
1.98
1.97
1.96
1.95
1.74
1.72
1.72
1.71
1.71
1.70
1.69
1.68
1.67
S
N
N
N
Br
S
N
N
N
Br
2.00
1.90
2.01
4.11
4.14
4.11
8.69
4.20
5.05
4.50
8.73
f1 (ppm)

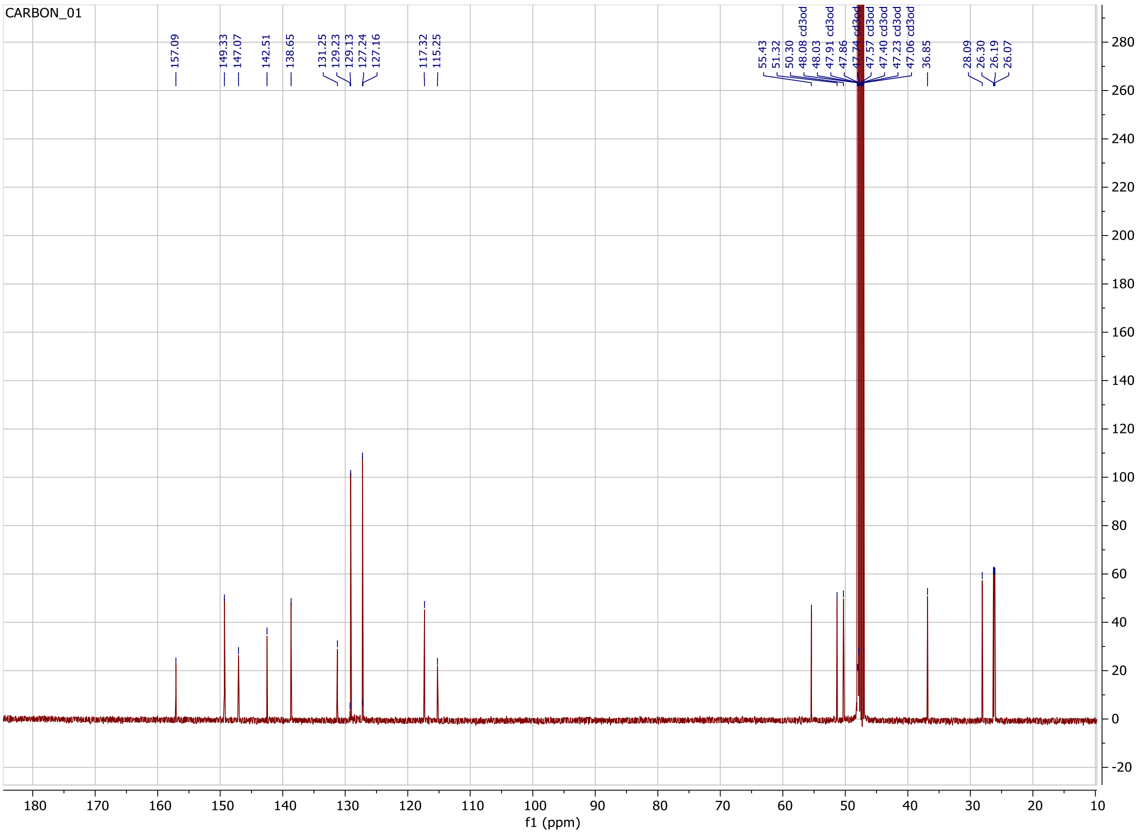
CARBON_01
157.09
149.33
147.07
142.51
138.65
131.25
129.23
129.13
127.24
127.16
117.32
115.25
55.43
51.32
50.30
48.08 cd3od
48.03
47.91 cd3od
47.86
47.74 cd3od
47.57 cd3od
47.40 cd3od
47.23 cd3od
47.06 cd3od
36.85
28.09
26.30
26.19
26.07
f1 (ppm)

Fg-19

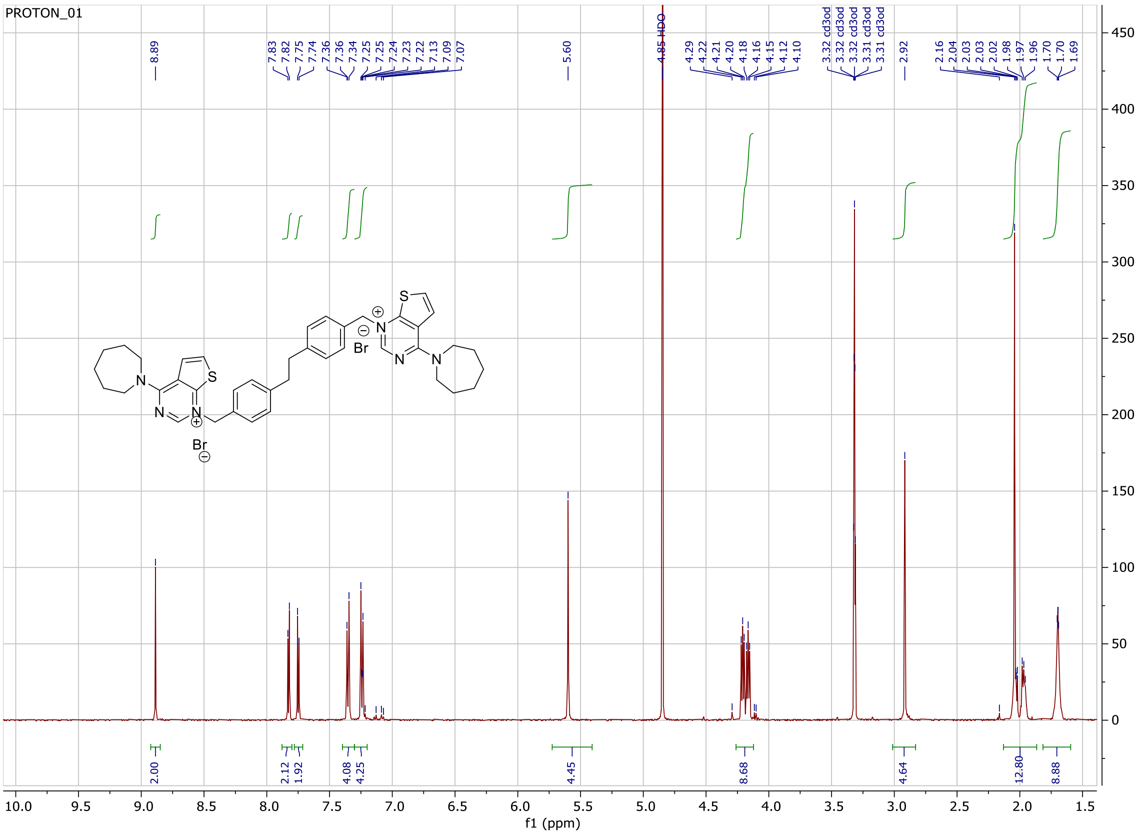
PROTON_01
8.89
7.83 7.82 7.75 7.74 7.36 7.36 7.34 7.25 7.25 7.24 7.23 7.22 7.13 7.09 7.07
5.60
4.85 HDO
4.29 4.22 4.21 4.20 4.18 4.16 4.15 4.12 4.10
3.32 cd3od 3.32 cd3od 3.32 cd3od 3.31 cd3od 3.31 cd3od
2.92
2.16 2.04 2.03 2.03 2.02 1.98 1.97 1.96 1.70 1.70 1.69
2.00 2.12 1.92 4.08 4.25 4.45 8.68 4.64 12.80 8.88
f1 (ppm)
Br
N
S

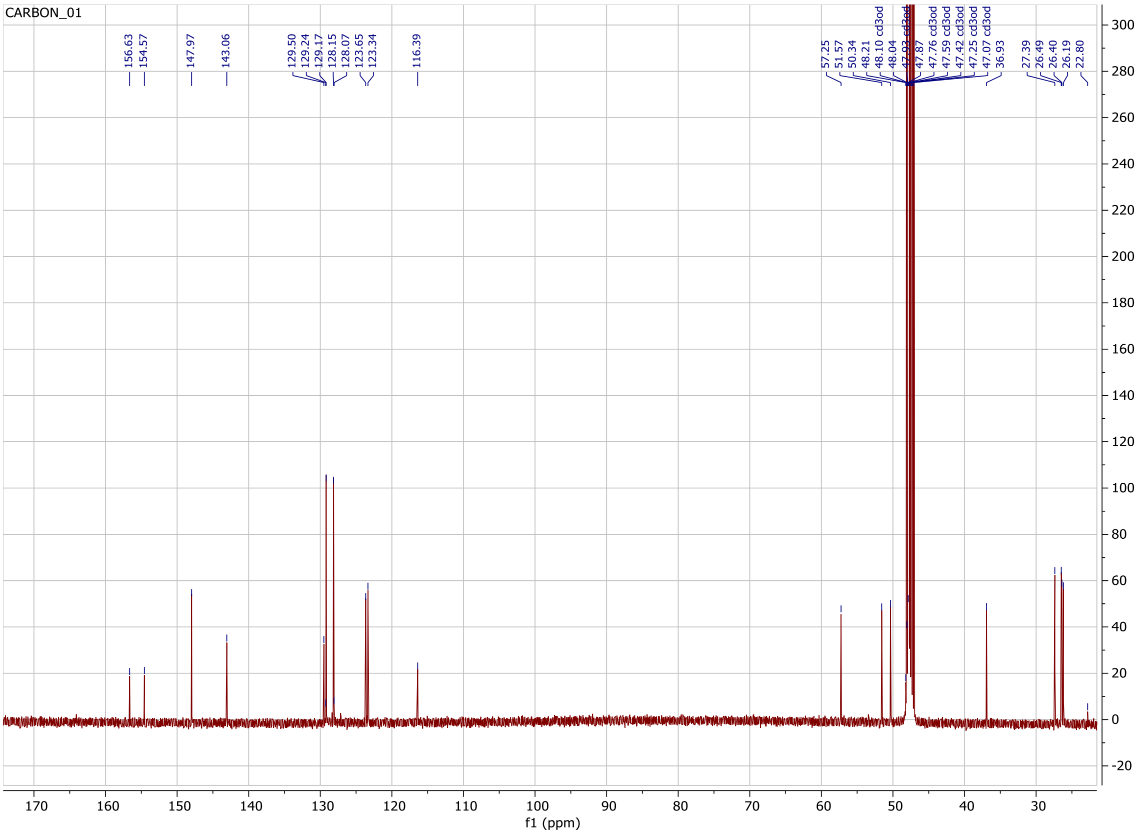
CARBON_01
156.63 154.57 147.97 143.06
129.50 129.24 129.17 128.15 128.07 123.65 123.34 116.39
57.25 51.57 50.34 48.21 48.10 cd3od 48.04 47.93 cd3od 47.87 47.76 cd3od 47.59 cd3od 47.42 cd3od 47.25 cd3od 47.07 cd3od 36.93
27.39 26.49 26.40 26.19 22.80
f1 (ppm)

Ff-1

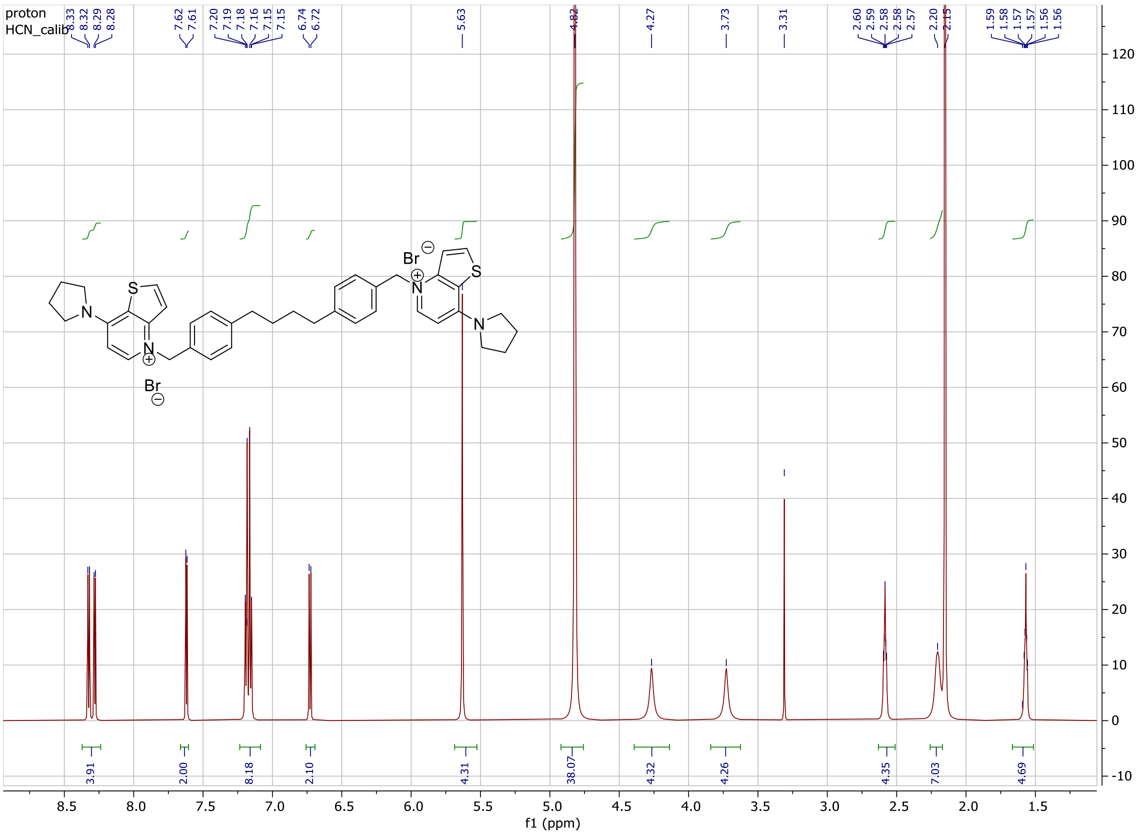

proton
HCN_calib
8.33 8.32 8.29 8.28
7.62 7.61
7.20 7.19 7.18 7.16 7.15 7.15
6.74 6.72
5.63
4.82
4.27
3.73
3.31
2.60 2.59 2.58 2.58 2.57
2.20 2.15
1.59 1.58 1.57 1.57 1.56 1.56
3.91 2.00 8.18 2.10 4.31 38.07 4.32 4.26 4.35 7.03 4.69
S N N Br
f1 (ppm)

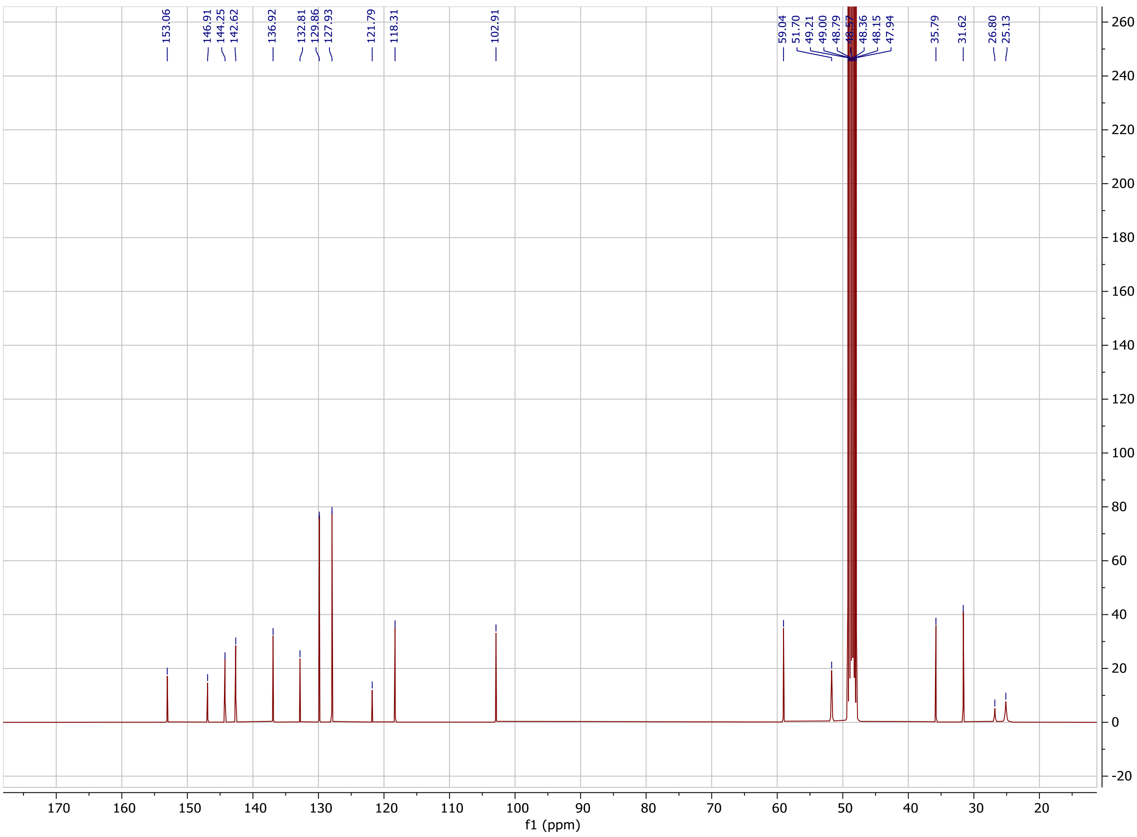

153.06
146.91 144.25 142.62
136.92
132.81 129.86 127.93
121.79 118.31
102.91
59.04 51.70 49.21 49.00 48.79 48.57 48.36 48.15 47.94
35.79 31.62 26.80 25.13
f1 (ppm)

Ff-7

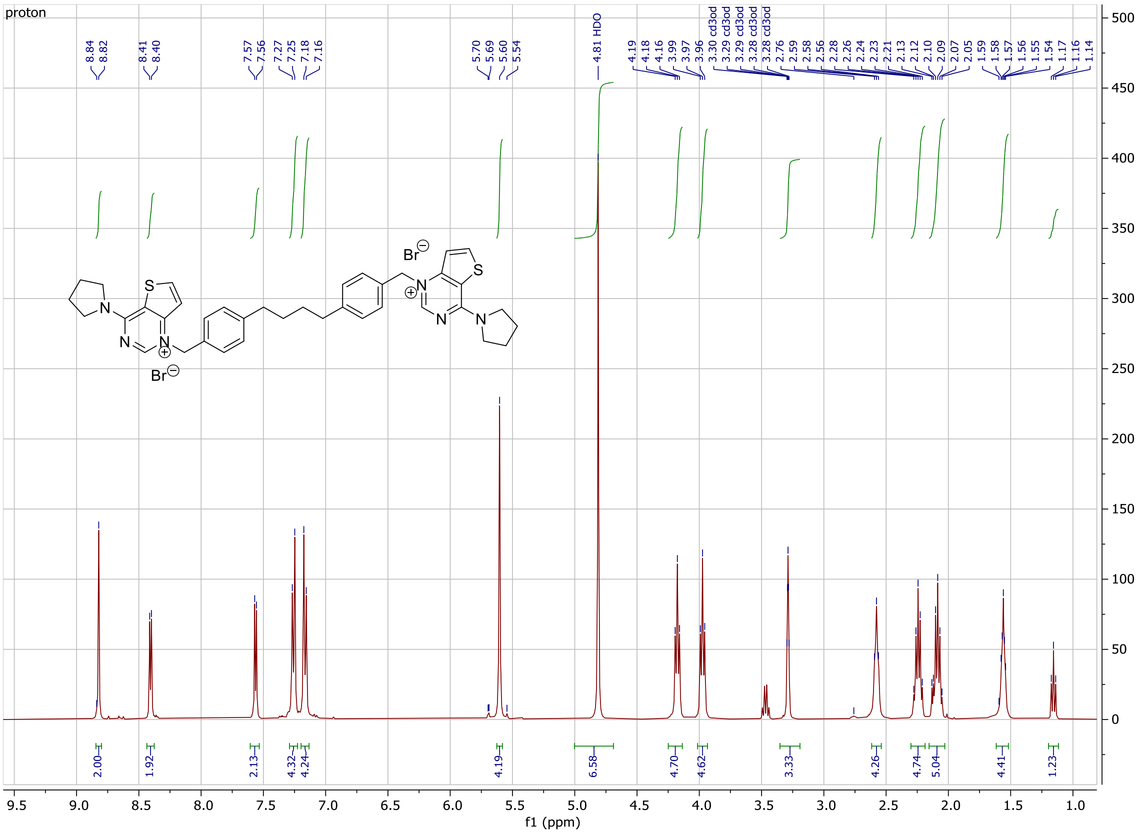

proton
8.84
8.82
8.41
8.40
7.57
7.56
7.27
7.25
7.18
7.16
5.70
5.69
5.60
5.54
4.81 HDO
4.19
4.18
4.16
3.99
3.97
3.96
3.30 cd3od
3.29 cd3od
3.29 cd3od
3.28 cd3od
3.28 cd3od
2.76
2.59
2.58
2.56
2.28
2.26
2.24
2.23
2.21
2.13
2.12
2.10
2.09
2.07
2.05
1.59
1.58
1.57
1.56
1.55
1.54
1.17
1.16
1.14
2.00
1.92
2.13
4.32
4.24
4.19
6.58
4.70
4.62
3.33
4.26
4.74
5.04
4.41
1.23
Br
S
N
N
N
Br
S
N
N
N
f1 (ppm)

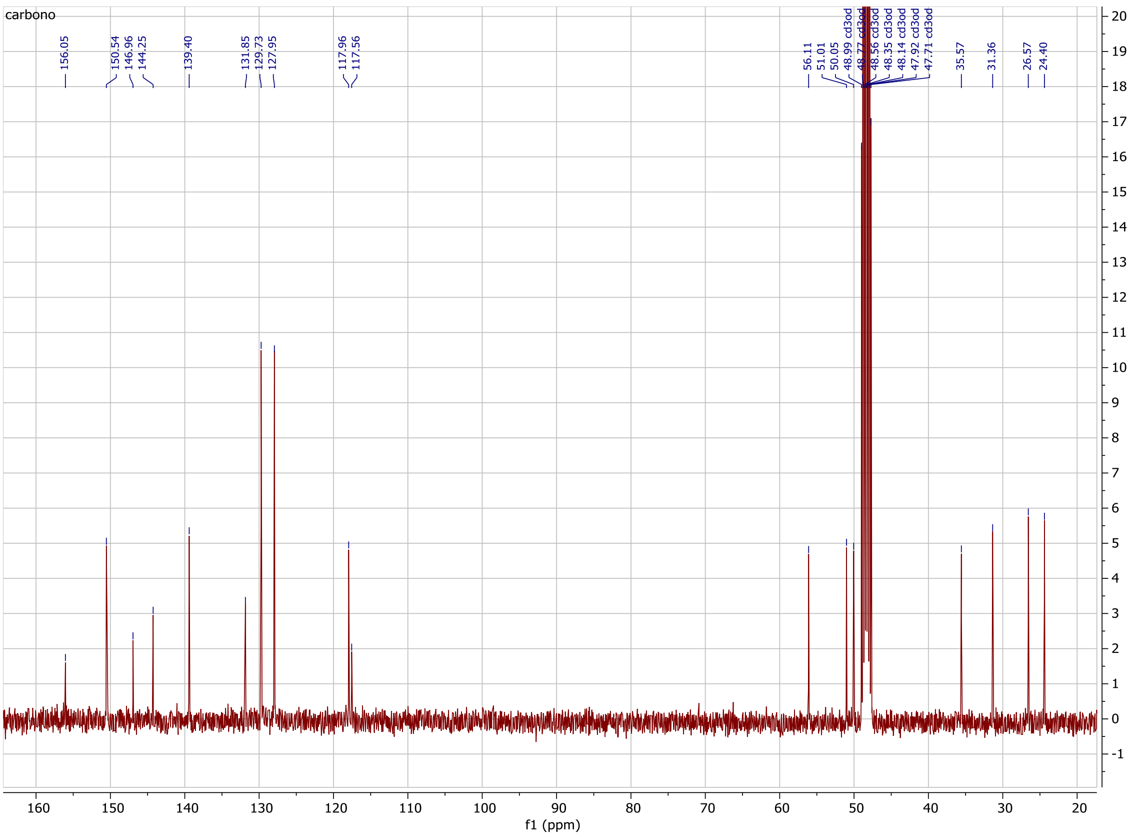

carbono
156.05
150.54
146.96
144.25
139.40
131.85
129.73
127.95
117.96
117.56
56.11
51.01
50.05
48.99 cd3od
48.77 cd3od
48.56 cd3od
48.35 cd3od
48.14 cd3od
47.92 cd3od
47.71 cd3od
35.57
31.36
26.57
24.40
f1 (ppm)

Ff-3

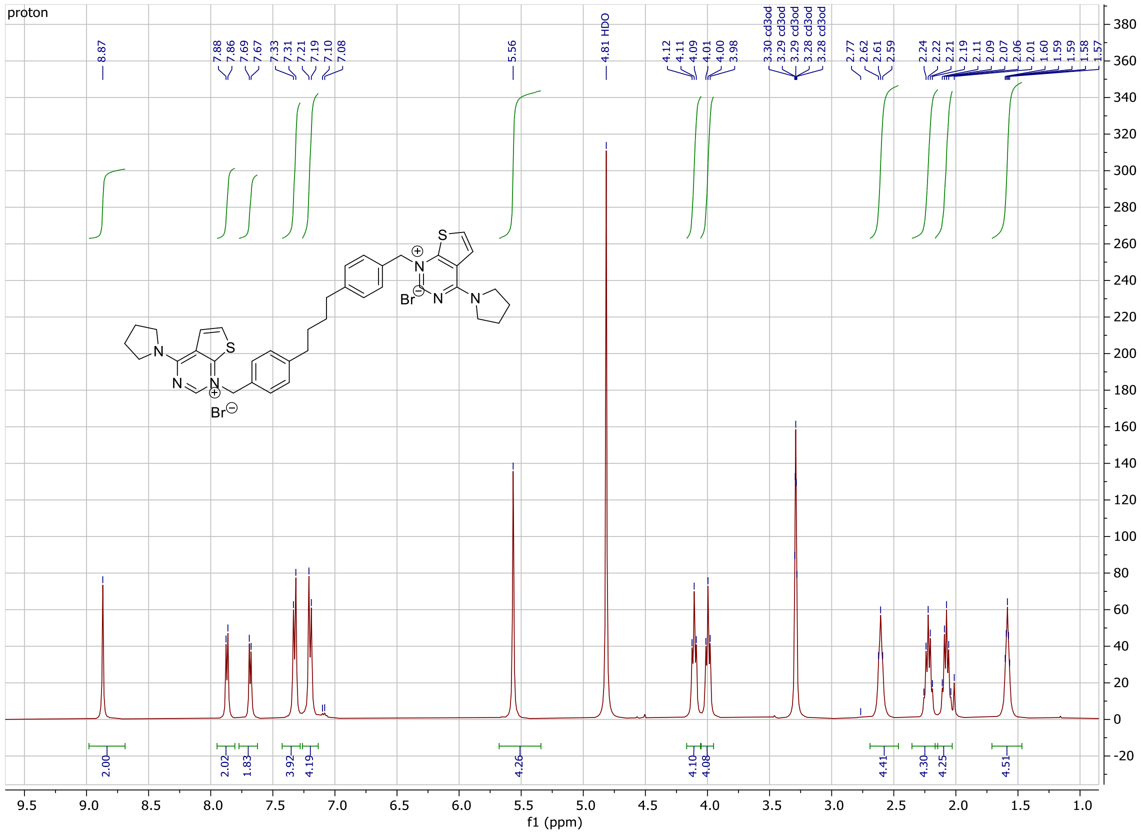
proton
8.87
7.88 7.86 7.69 7.67 7.33 7.31 7.21 7.19 7.10 7.08
5.56
4.81 HDO
4.12 4.11 4.09 4.01 4.00 3.98
3.30 cd3od 3.29 cd3od 3.29 cd3od 3.28 cd3od 3.28 cd3od
2.77 2.62 2.61 2.59
2.24 2.22 2.21 2.19 2.11 2.09 2.07 2.06 2.01 1.60 1.59 1.59 1.58 1.57
2.00 2.02 1.83 3.92 4.19 4.26 4.10 4.08 4.41 4.30 4.25 4.51
S
N
Br
f1 (ppm)

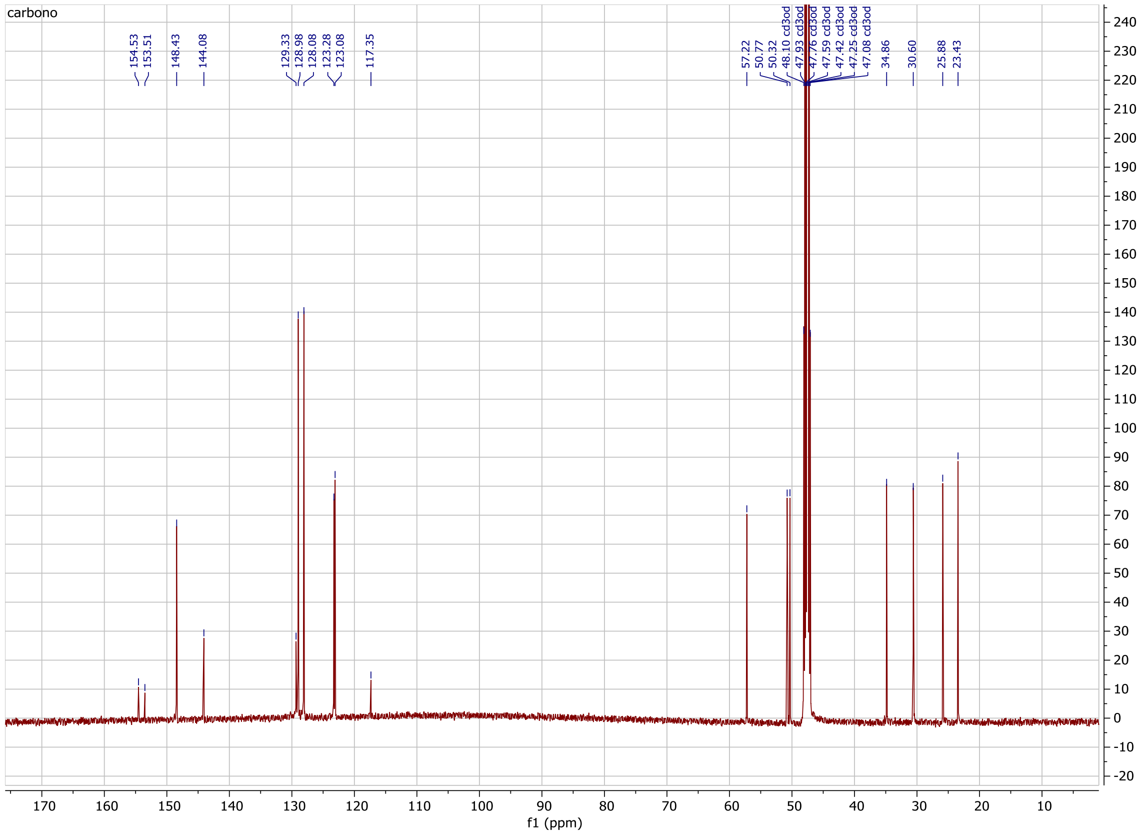
carbono
154.53 153.51 148.43 144.08
129.33 128.98 128.08 123.28 123.08 117.35
57.22 50.77 50.32 48.10 cd3od 47.93 cd3od 47.76 cd3od 47.59 cd3od 47.42 cd3od 47.25 cd3od 47.08 cd3od
34.86 30.60 25.88 23.43
f1 (ppm)

Fa-33

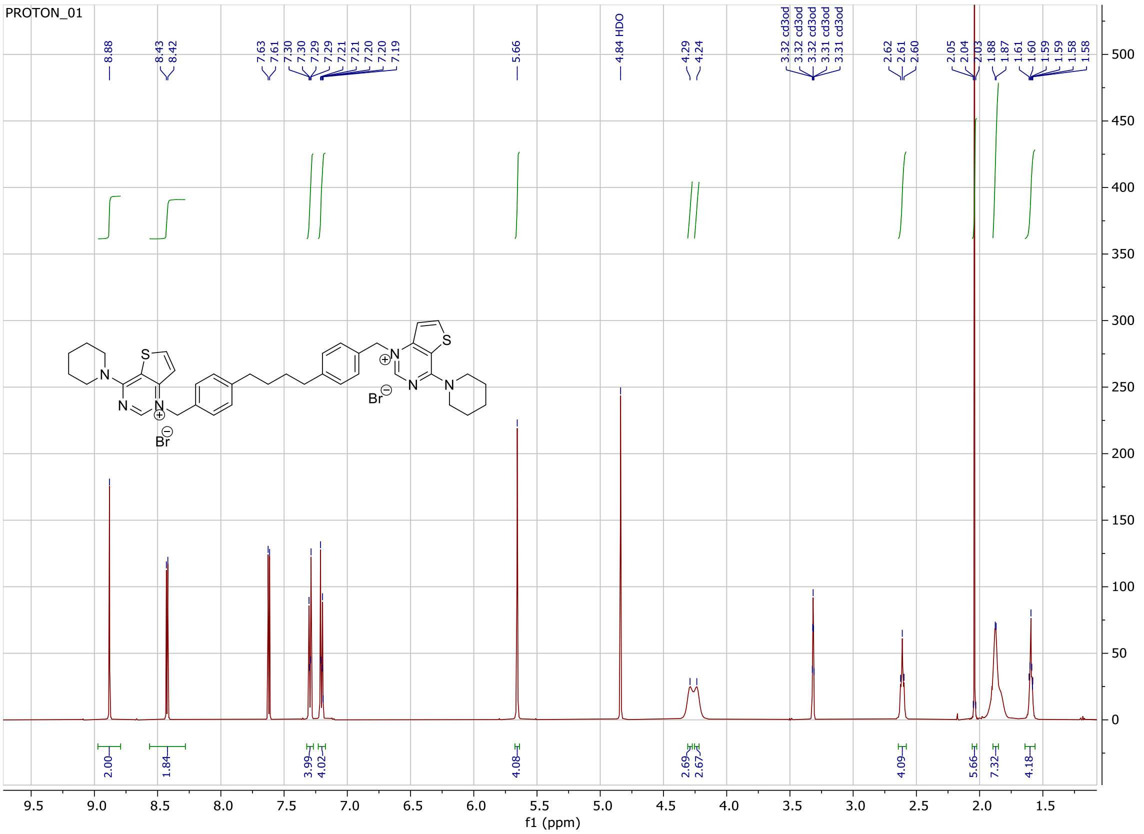
PROTON_01
8.88
8.43
8.42
7.63
7.61
7.30
7.29
7.29
7.21
7.21
7.20
7.20
7.19
5.66
4.84 HDO
4.29
4.24
3.32 cd3od
3.32 cd3od
3.32 cd3od
3.31 cd3od
3.31 cd3od
2.62
2.61
2.60
2.05
2.04
2.03
1.88
1.87
1.61
1.60
1.59
1.59
1.58
1.58
2.00
1.84
3.99
4.02
4.08
2.69
2.67
4.09
5.66
7.32
4.18
f1 (ppm)

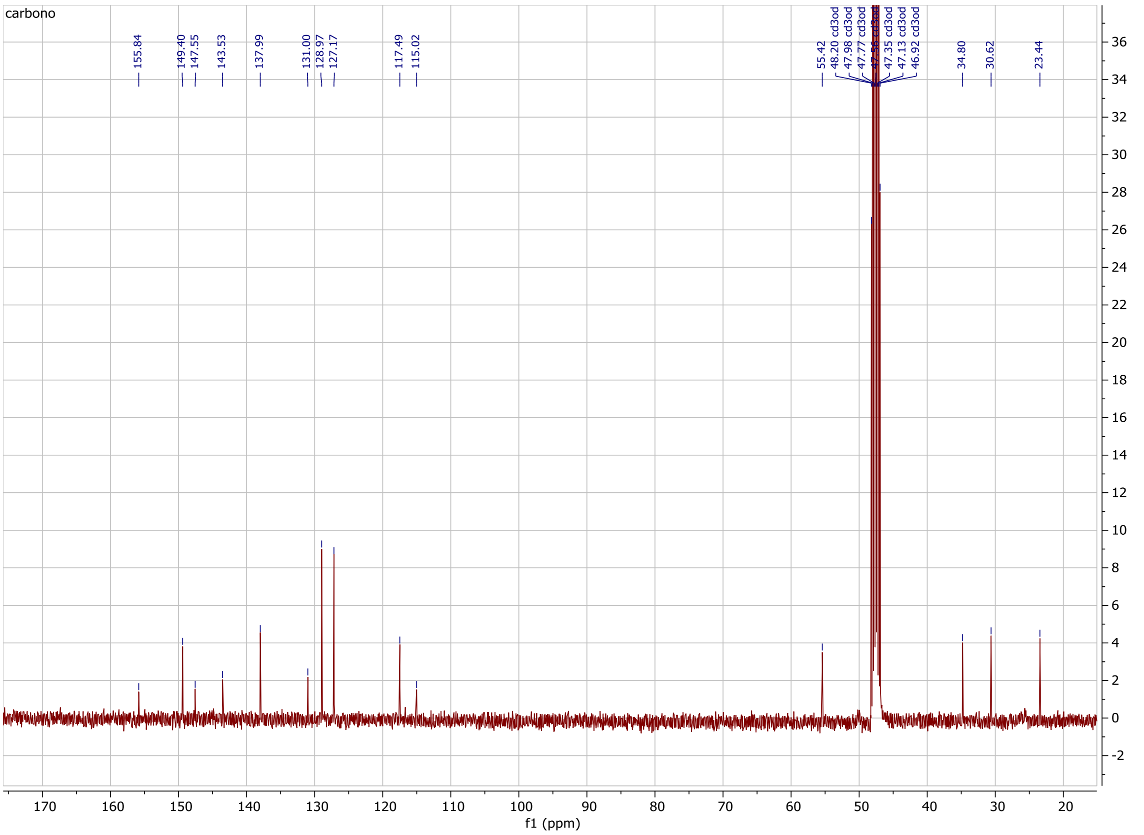
carbono
155.84
149.40
147.55
143.53
137.99
131.00
128.97
127.17
117.49
115.02
55.42
48.20 cd3od
47.98 cd3od
47.77 cd3od
47.56 cd3od
47.35 cd3od
47.13 cd3od
46.92 cd3od
34.80
30.62
23.44
f1 (ppm)

Ff-6

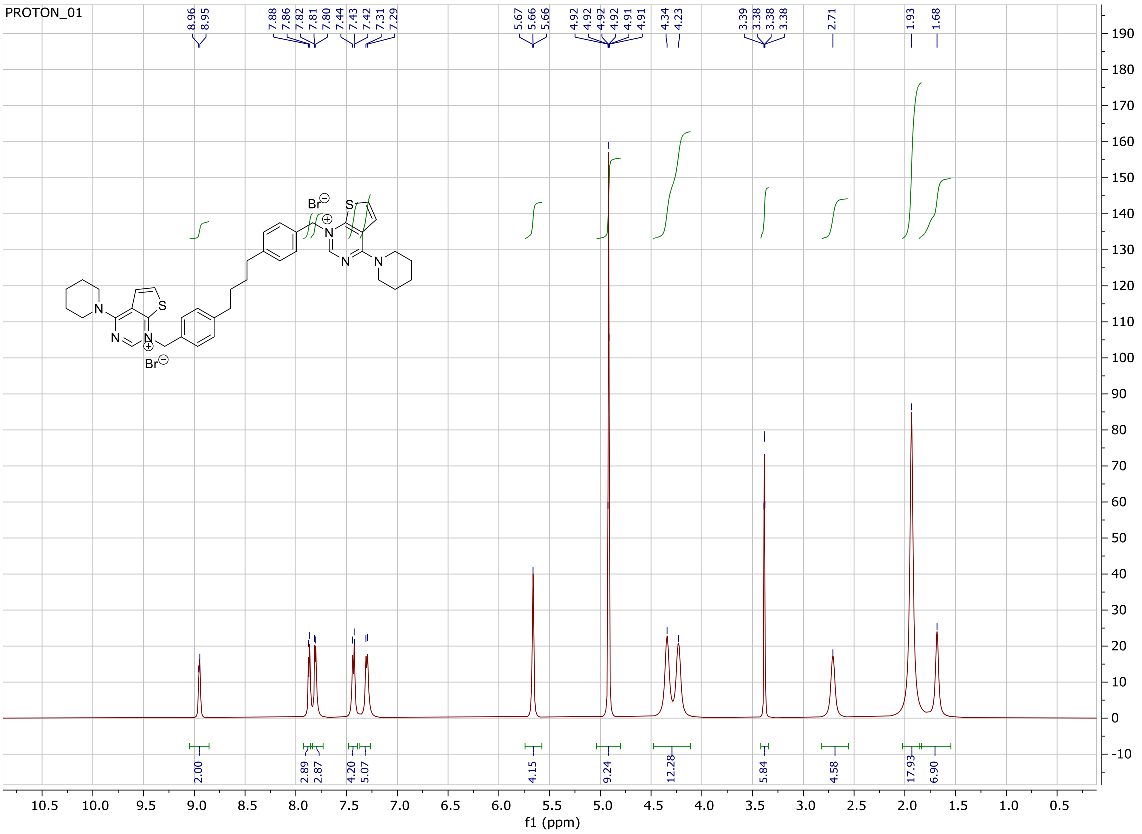
PROTON_01
8.96 8.95 7.88 7.86 7.82 7.81 7.80 7.44 7.43 7.42 7.31 7.29 5.67 5.66 5.66 4.92 4.92 4.92 4.92 4.91 4.91 4.34 4.23 3.39 3.38 3.38 3.38 2.71 1.93 1.68
2.00 2.89 2.87 4.20 5.07 4.15 9.24 12.28 5.84 4.58 17.93 6.90
Br⊖
S
N
N
Br⊖
f1 (ppm)

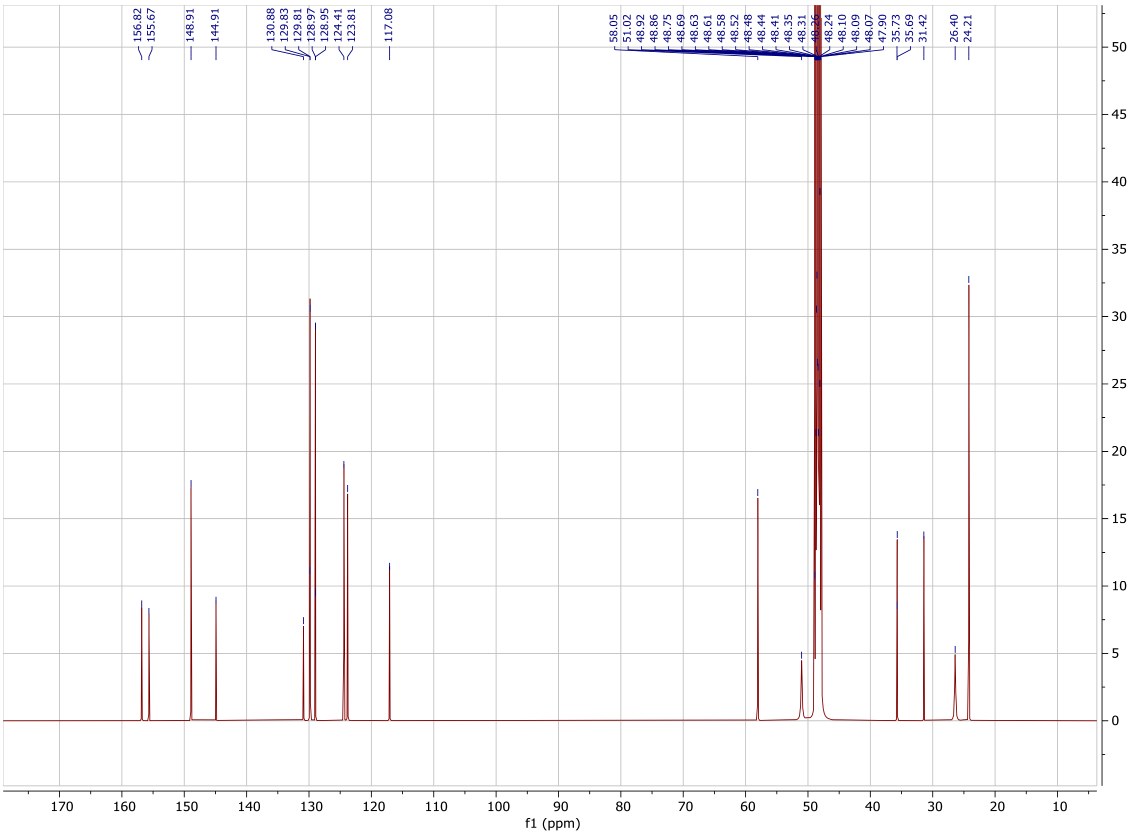
156.82 155.67 148.91 144.91 130.88 129.83 129.81 128.97 128.95 124.41 123.81 117.08
58.05 51.02 48.92 48.86 48.75 48.69 48.63 48.61 48.58 48.52 48.48 48.44 48.41 48.35 48.31 48.26 48.24 48.10 48.09 48.07 47.90 35.73 35.69 31.42 26.40 24.21
f1 (ppm)

Fa-29

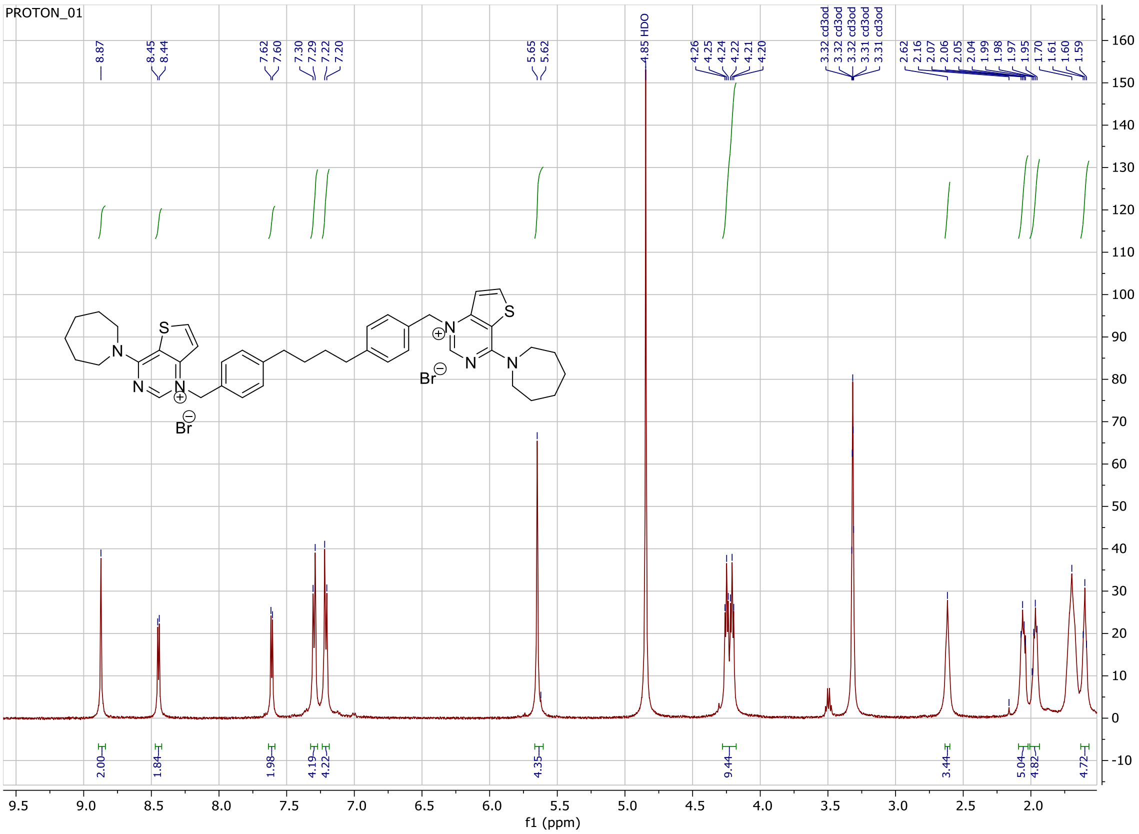
PROTON_01
8.87
8.45 8.44
7.62 7.60
7.30 7.29 7.22 7.20
5.65 5.62
4.85 HDO
4.26 4.25 4.24 4.22 4.21 4.20
3.32 cd3od 3.32 cd3od 3.32 cd3od 3.31 cd3od 3.31 cd3od 3.31 cd3od
2.62 2.16 2.07 2.06 2.05 2.04 1.99 1.98 1.97 1.95 1.70 1.61 1.60 1.59
2.00 1.84 1.98 4.19 4.22 4.35 9.44 3.44 5.04 4.82 4.72
S
N
N
Br
f1 (ppm)

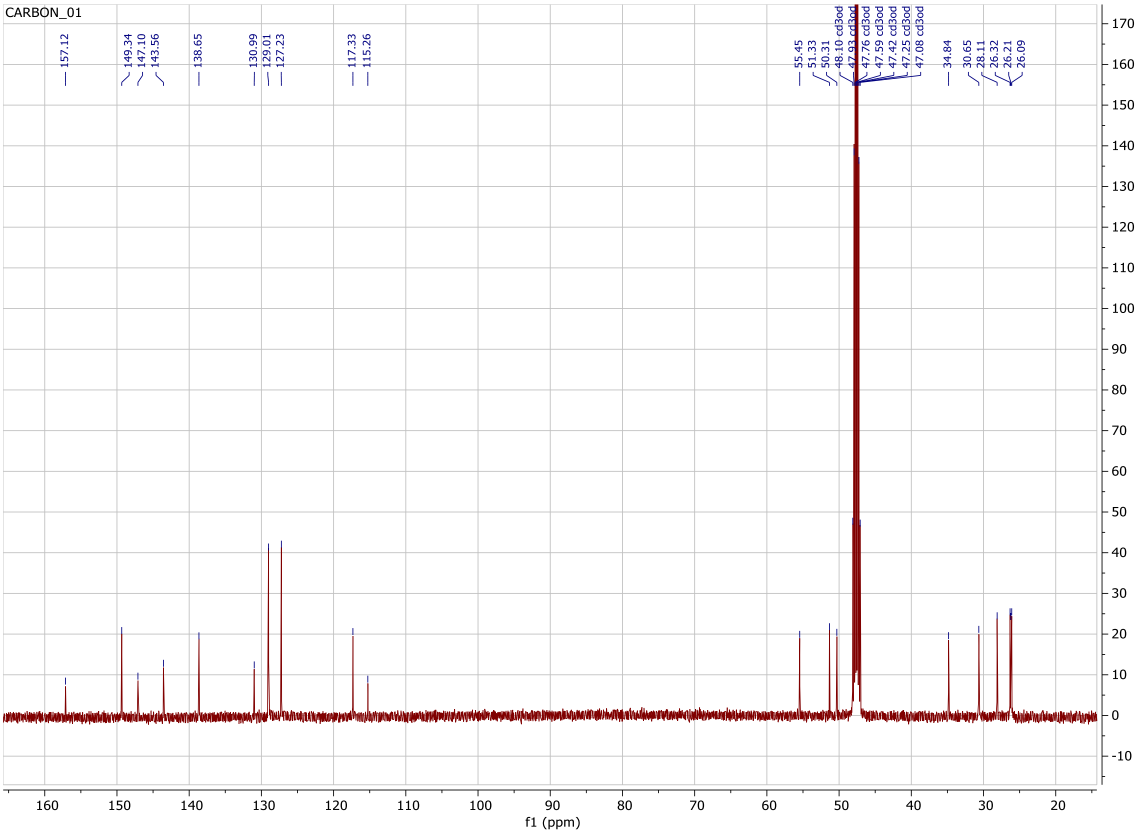
CARBON_01
157.12
149.34 147.10 143.56
138.65
130.99 129.01 127.23
117.33 115.26
55.45 51.33 50.31 48.10 cd3od 47.93 cd3od 47.76 cd3od 47.59 cd3od 47.42 cd3od 47.25 cd3od 47.08 cd3od
34.84 30.65 28.11 26.32 26.21 26.09
f1 (ppm)

Ff-35

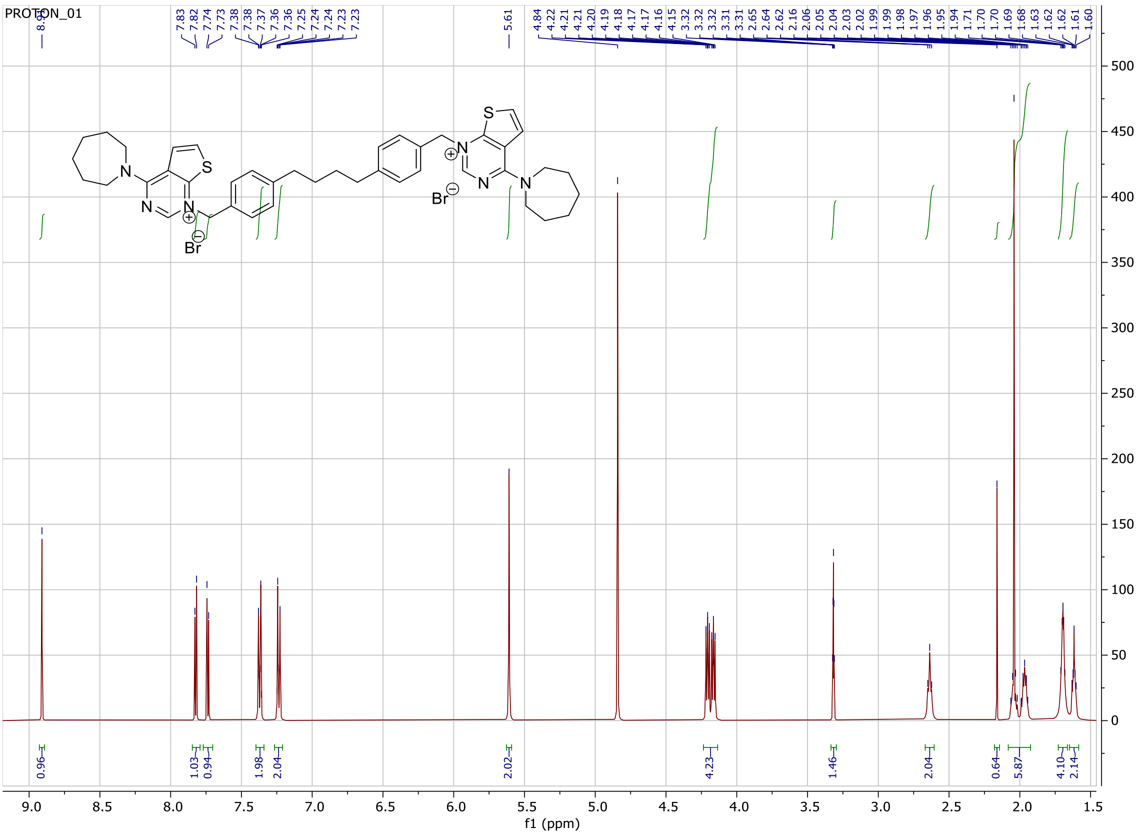

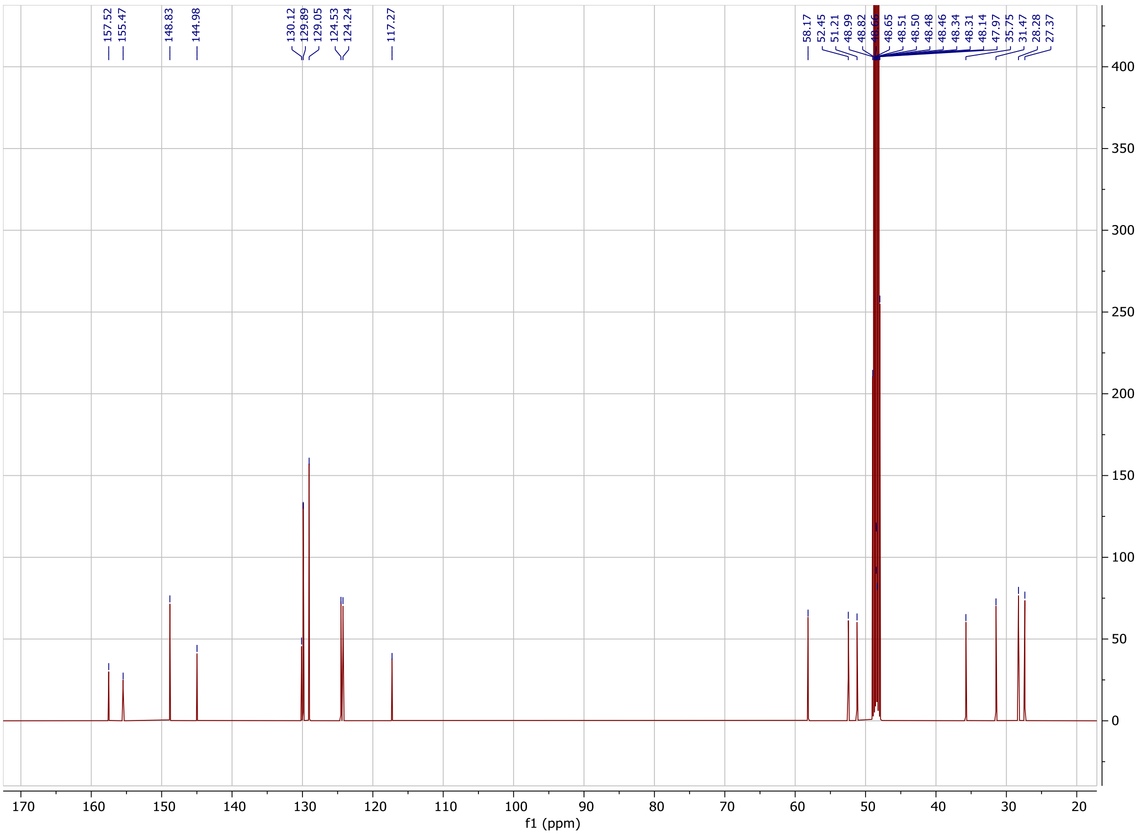

Ff-2

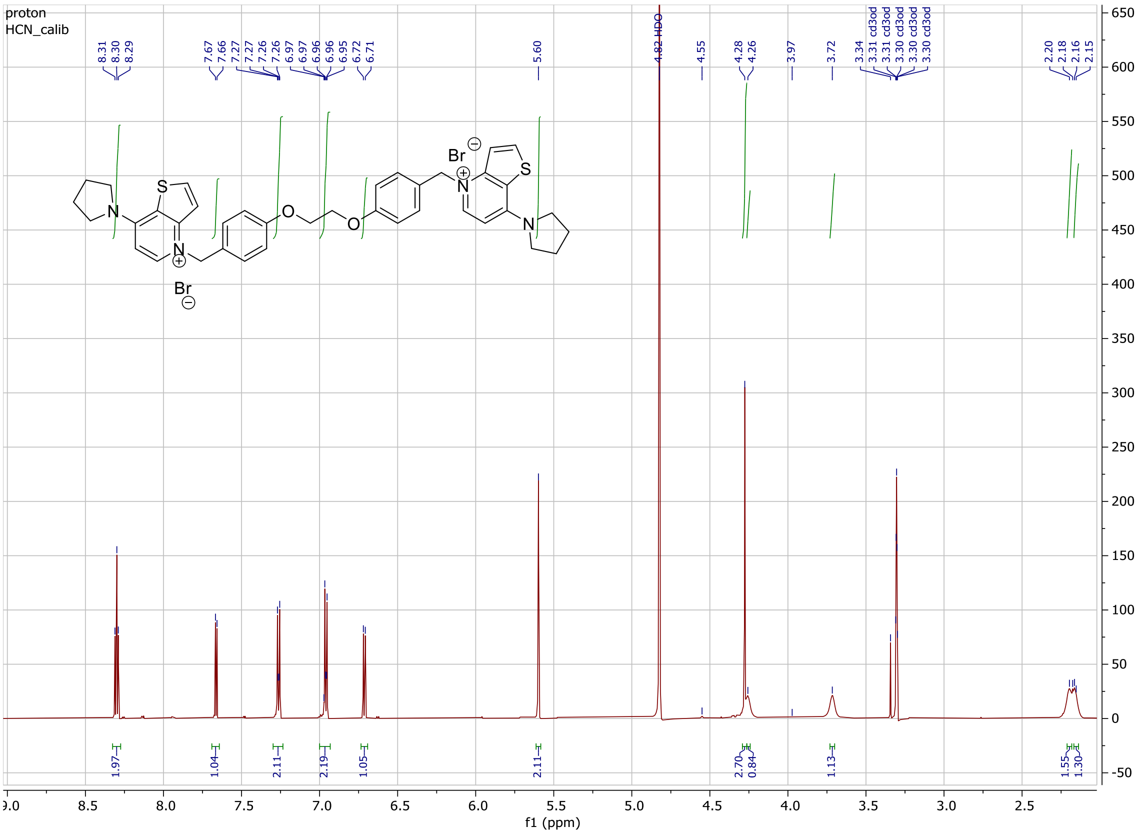
proton
HCN_calib
8.31
8.30
8.29
7.67
7.66
7.27
7.27
7.26
7.26
6.97
6.97
6.96
6.96
6.95
6.72
6.71
5.60
4.82 HDO
4.55
4.28
4.26
3.97
3.72
3.34
3.31 cd3od
3.31 cd3od
3.30 cd3od
3.30 cd3od
3.30 cd3od
2.20
2.18
2.16
2.15
1.97
1.04
2.11
2.19
1.05
2.11
2.70
0.84
1.13
1.55
1.30
f1 (ppm)

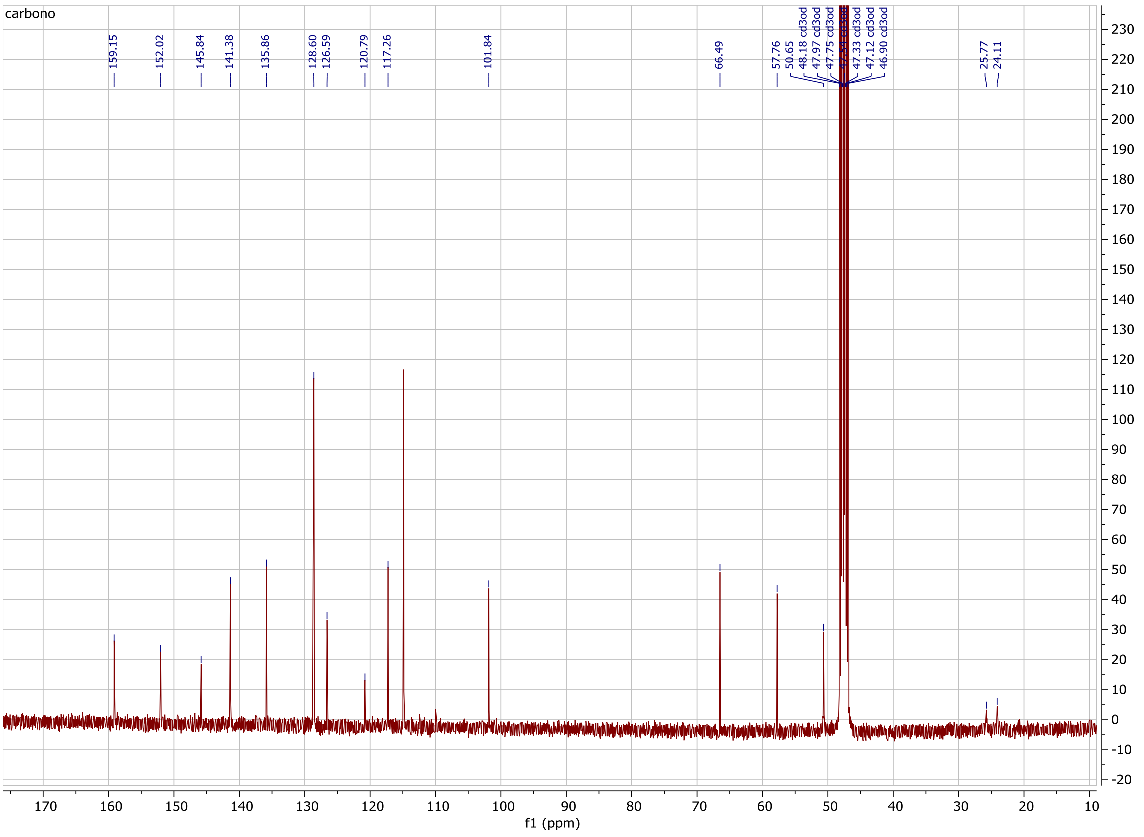
carbono
159.15
152.02
145.84
141.38
135.86
128.60
126.59
120.79
117.26
101.84
66.49
57.76
50.65
48.18 cd3od
47.97 cd3od
47.75 cd3od
47.54 cd3od
47.33 cd3od
47.12 cd3od
46.90 cd3od
25.77
24.11
f1 (ppm)

Ff-8

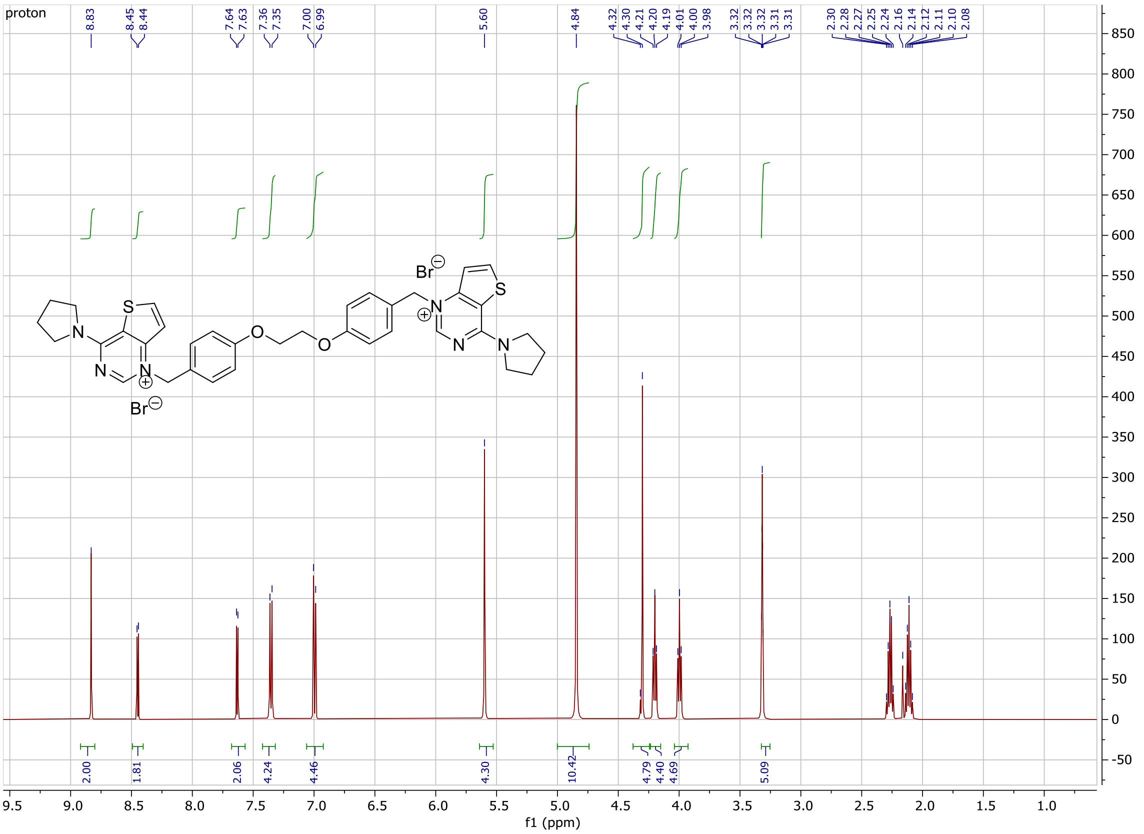

proton
8.83
8.45
8.44
7.64
7.63
7.36
7.35
7.00
6.99
5.60
4.84
4.32
4.30
4.21
4.20
4.19
4.01
4.00
3.98
3.32
3.32
3.32
3.31
3.31
2.30
2.28
2.27
2.25
2.24
2.16
2.14
2.12
2.11
2.10
2.08
2.00
1.81
2.06
4.24
4.46
4.30
10.42
4.79
4.40
4.69
5.09
f1 (ppm)
Br
S
N
O

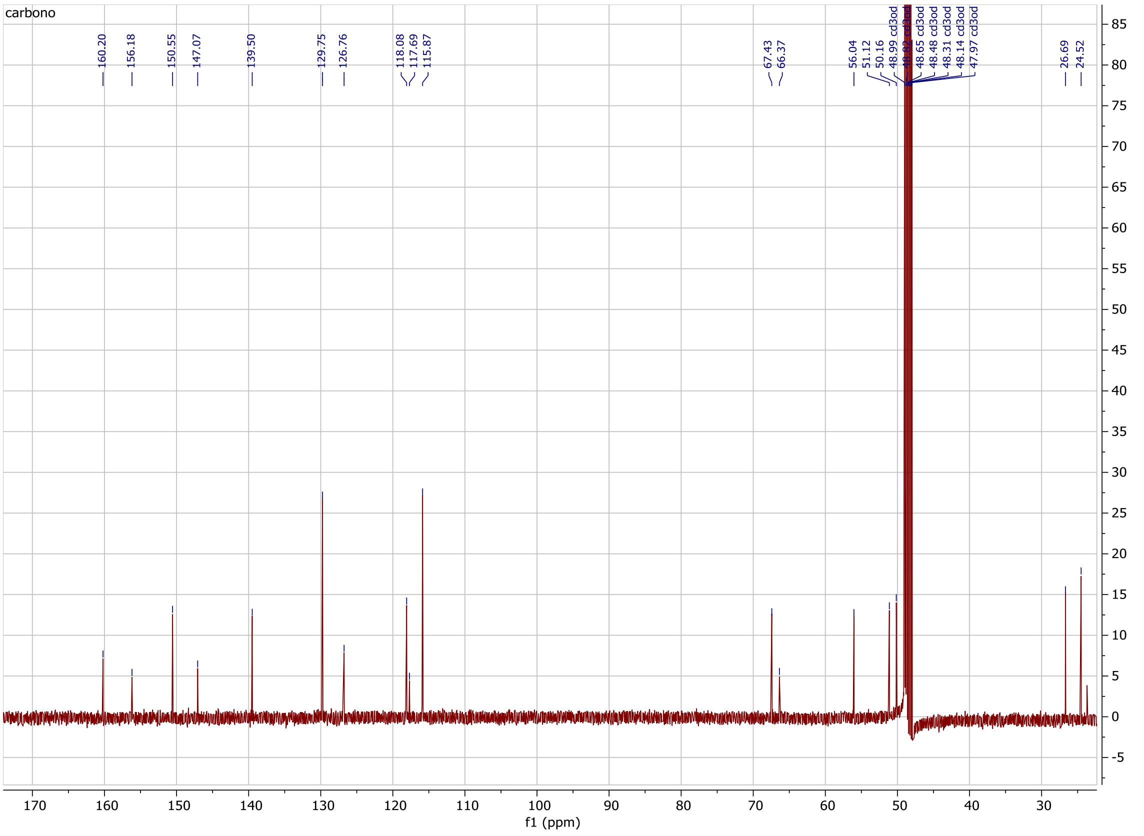

carbono
160.20
156.18
150.55
147.07
139.50
129.75
126.76
118.08
117.69
115.87
67.43
66.37
56.04
51.12
50.16
48.99 cd3od
48.82 cd3od
48.65 cd3od
48.48 cd3od
48.31 cd3od
48.14 cd3od
47.97 cd3od
26.69
24.52
f1 (ppm)

Ff-4

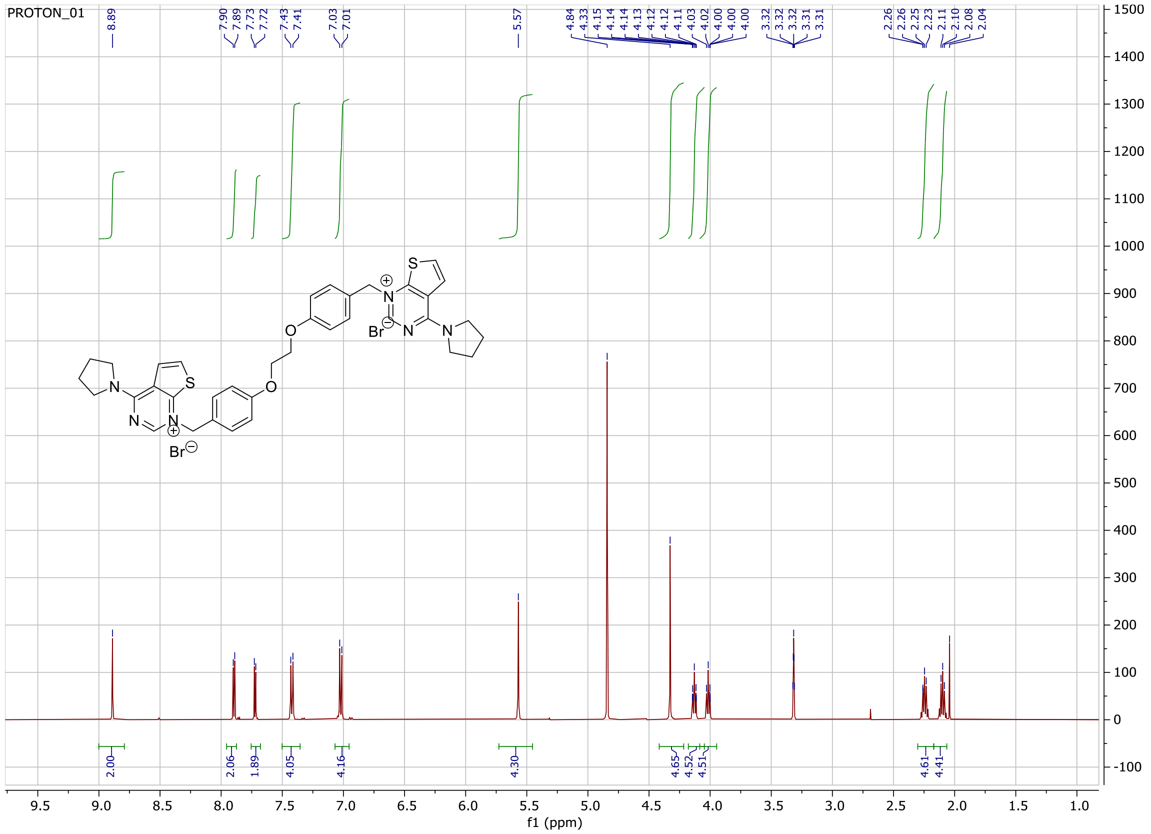

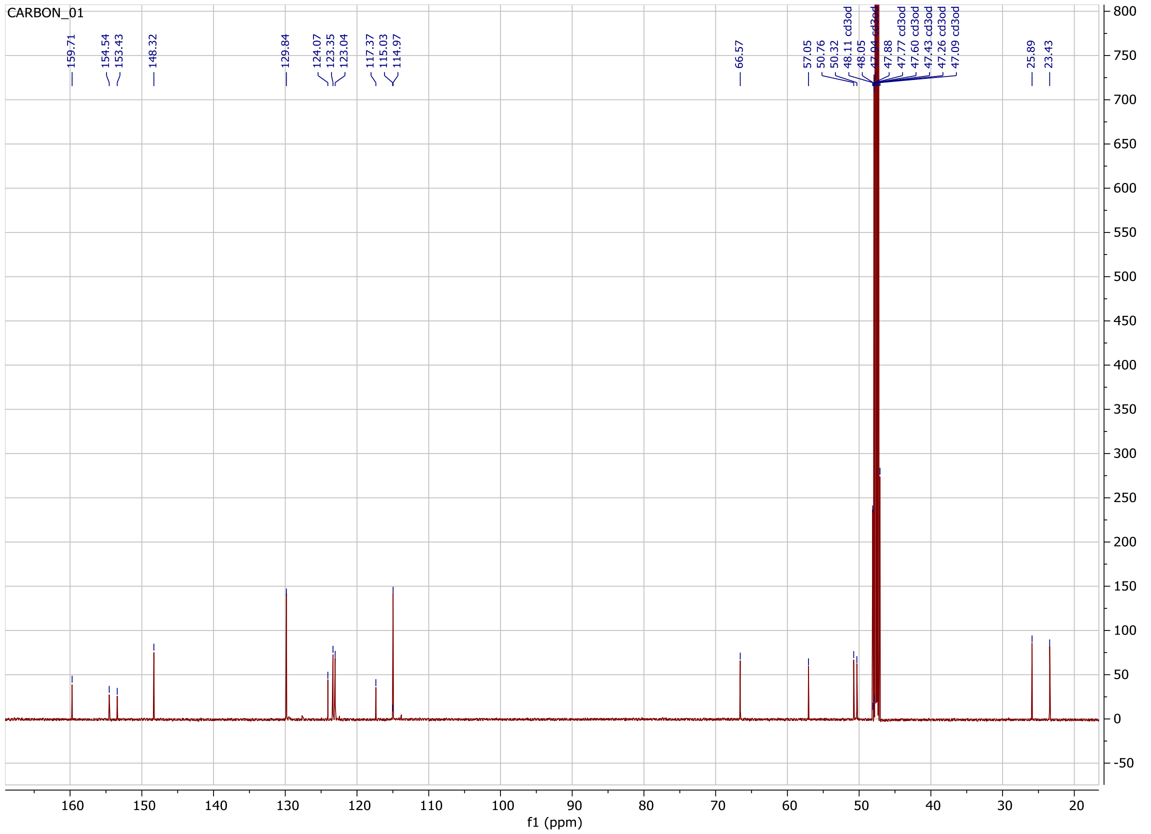

Fa-28

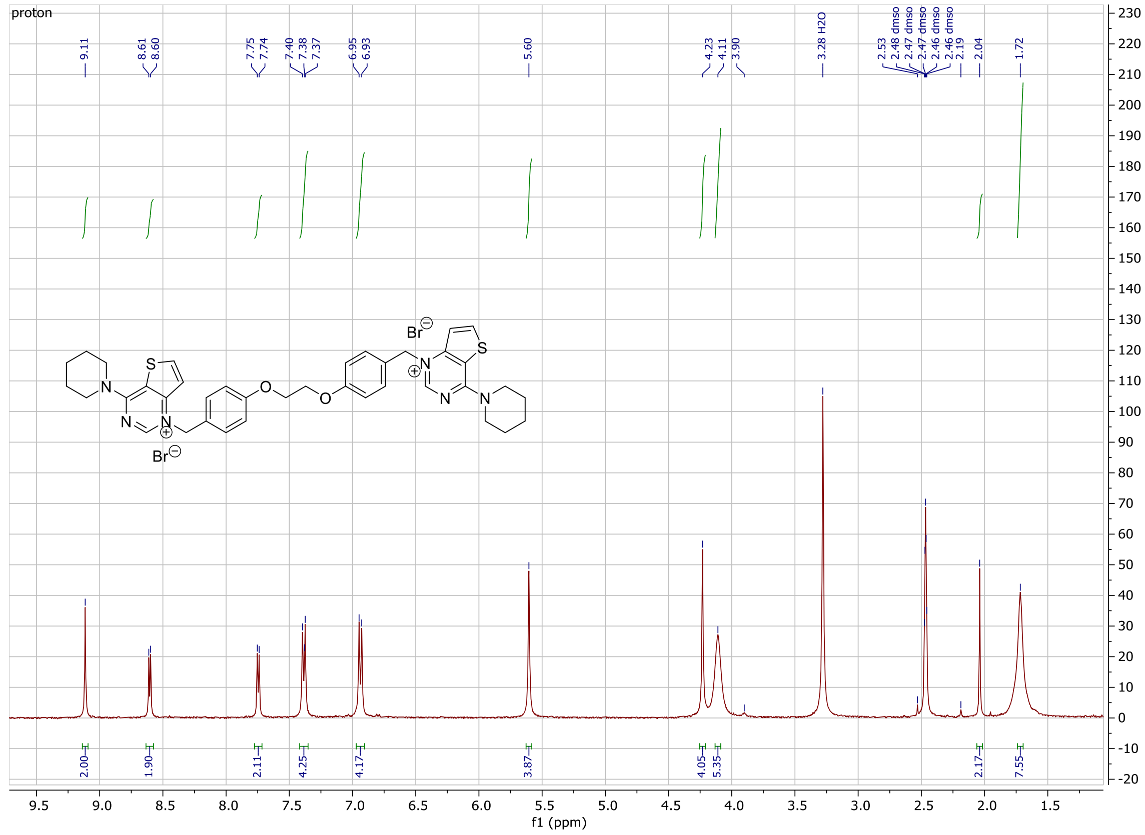
proton
9.11
8.61
8.60
7.75
7.74
7.40
7.38
7.37
6.95
6.93
5.60
4.23
4.11
3.90
3.28 H2O
2.53
2.48 dmso
2.47 dmso
2.47 dmso
2.46 dmso
2.46 dmso
2.19
2.04
1.72
2.00
1.90
2.11
4.25
4.17
3.87
4.05
5.35
2.17
7.55
Br
S
N
N
N
O
O
f1 (ppm)

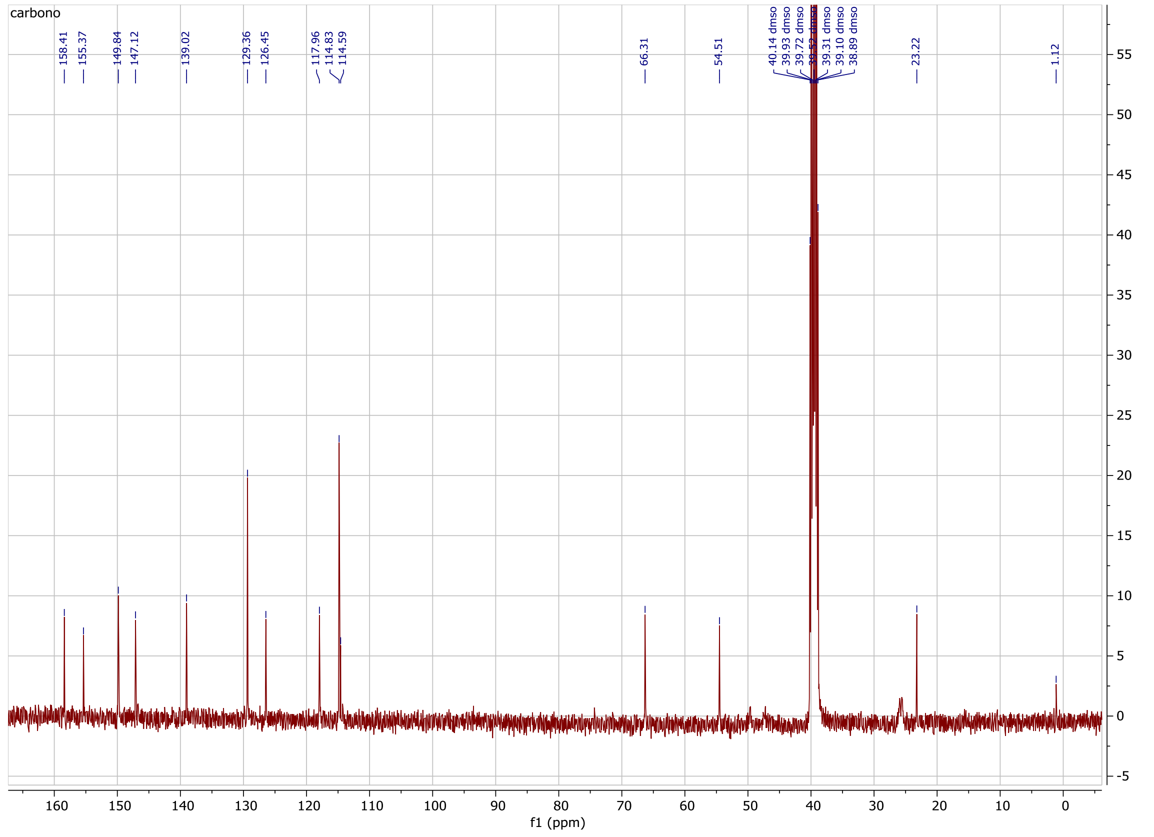
carbono
158.41
155.37
149.84
147.12
139.02
129.36
126.45
117.96
114.83
114.59
66.31
54.51
40.14 dmso
39.93 dmso
39.72 dmso
39.31 dmso
39.10 dmso
38.89 dmso
23.22
1.12
f1 (ppm)

Ff-5

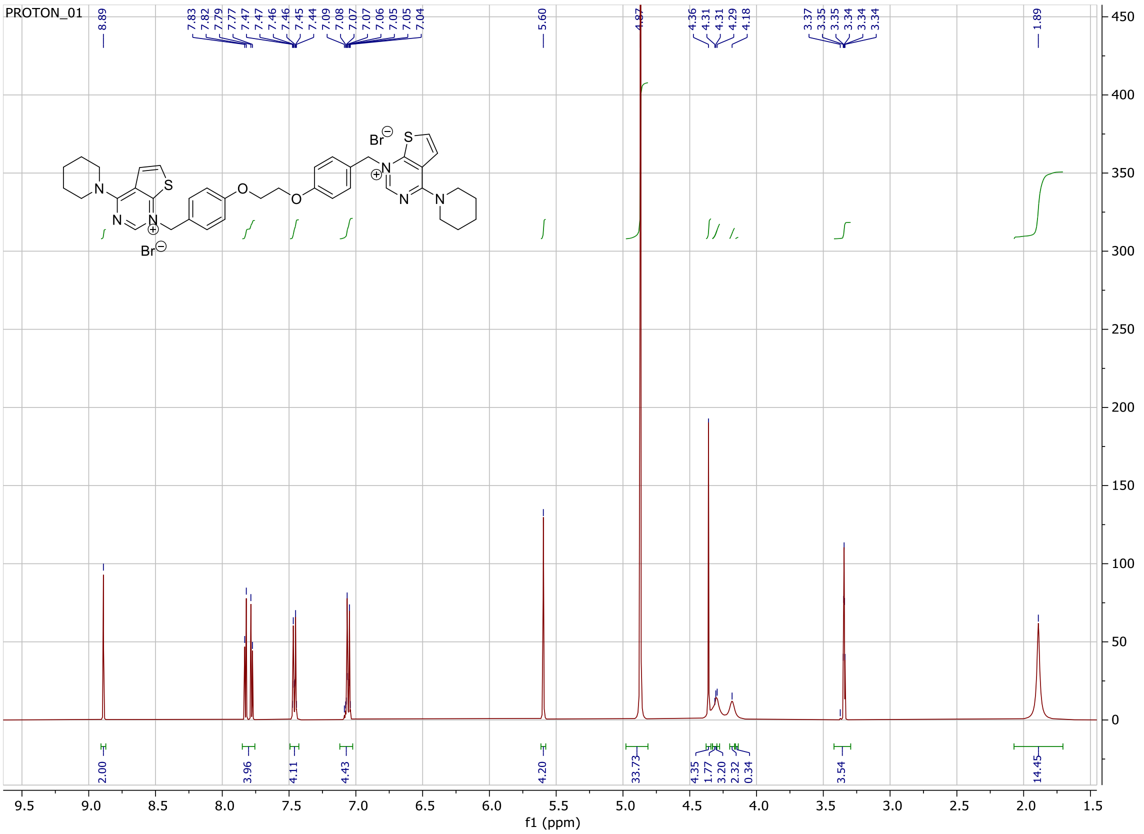

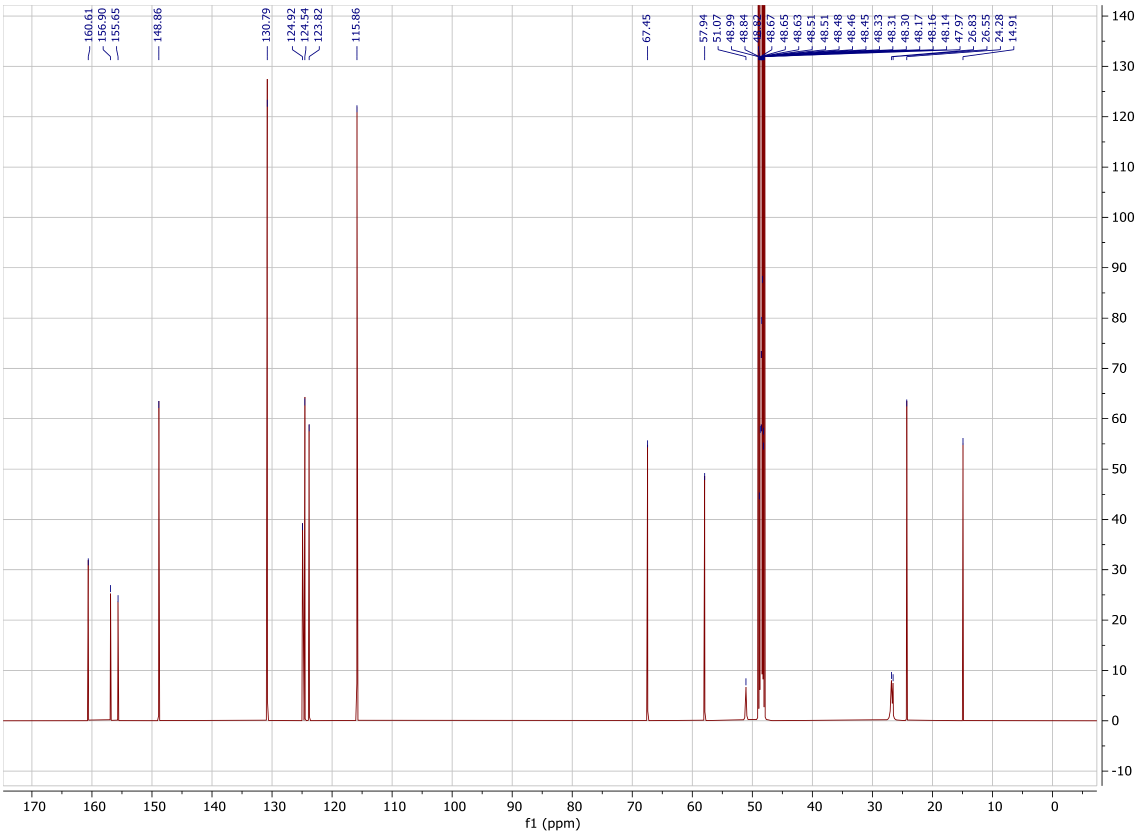

Ff-34

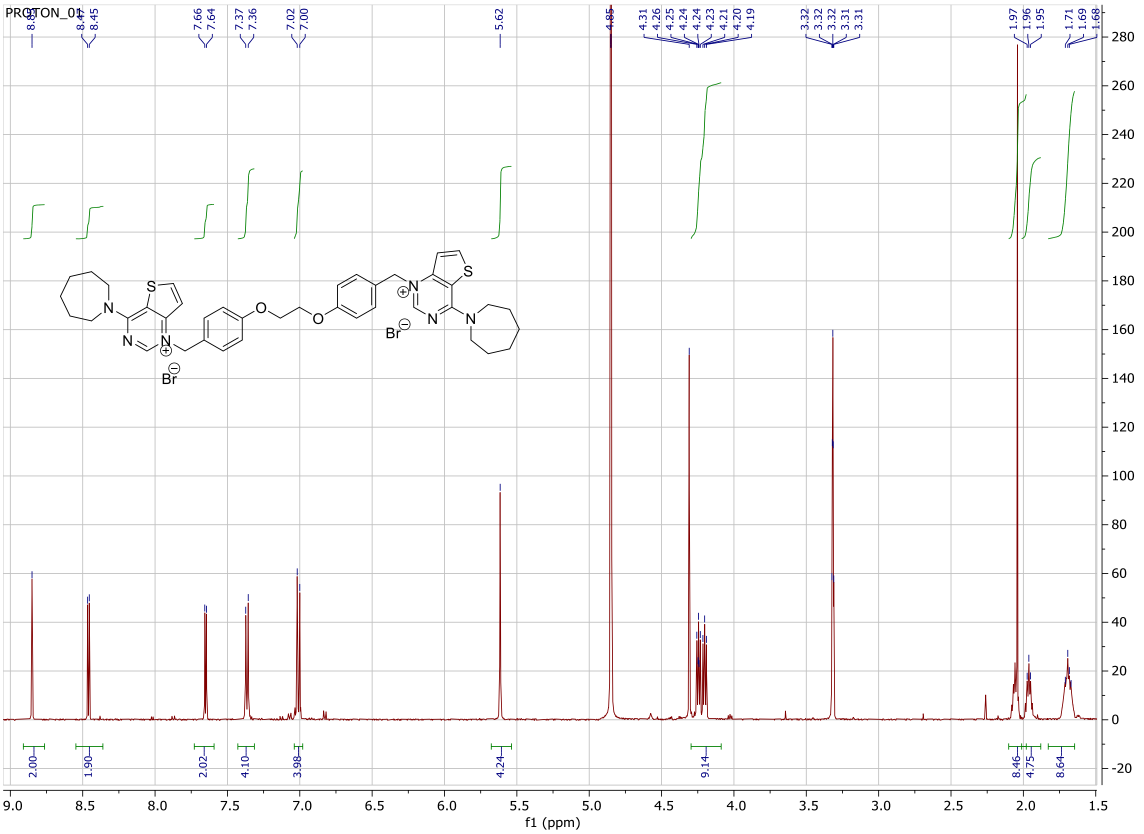
PROTON_01
8.47
8.45
7.66
7.64
7.37
7.36
7.02
7.00
5.62
4.85
4.31
4.26
4.25
4.24
4.24
4.23
4.21
4.20
4.19
3.32
3.32
3.32
3.31
3.31
1.97
1.96
1.95
1.71
1.69
1.68
2.00
1.90
2.02
4.10
3.98
4.24
9.14
8.46
4.75
8.64
S
N
N
O
O
N
S
N
N
Br
f1 (ppm)

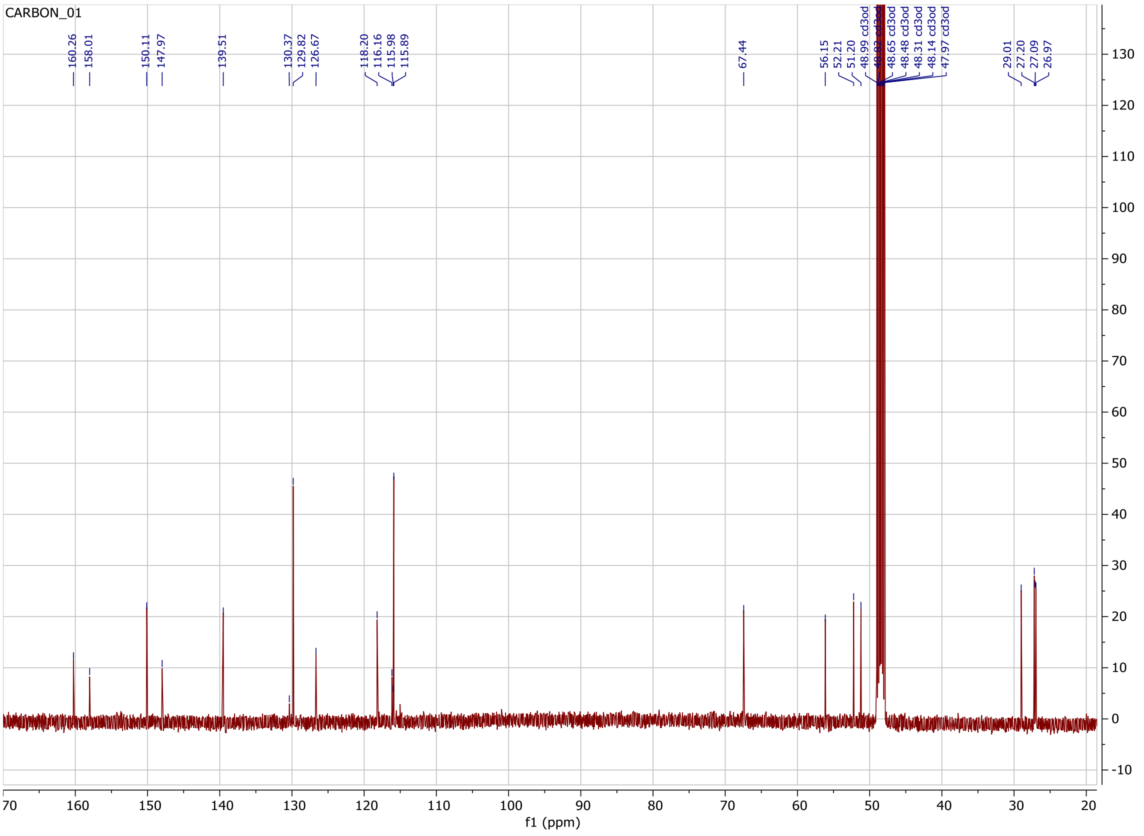
CARBON_01
160.26
158.01
150.11
147.97
139.51
130.37
129.82
126.67
118.20
116.16
115.98
115.89
67.44
56.15
52.21
51.20
48.99 cd3od
48.65 cd3od
48.48 cd3od
48.31 cd3od
48.14 cd3od
47.97 cd3od
29.01
27.20
27.09
26.97
f1 (ppm)

Ff-36

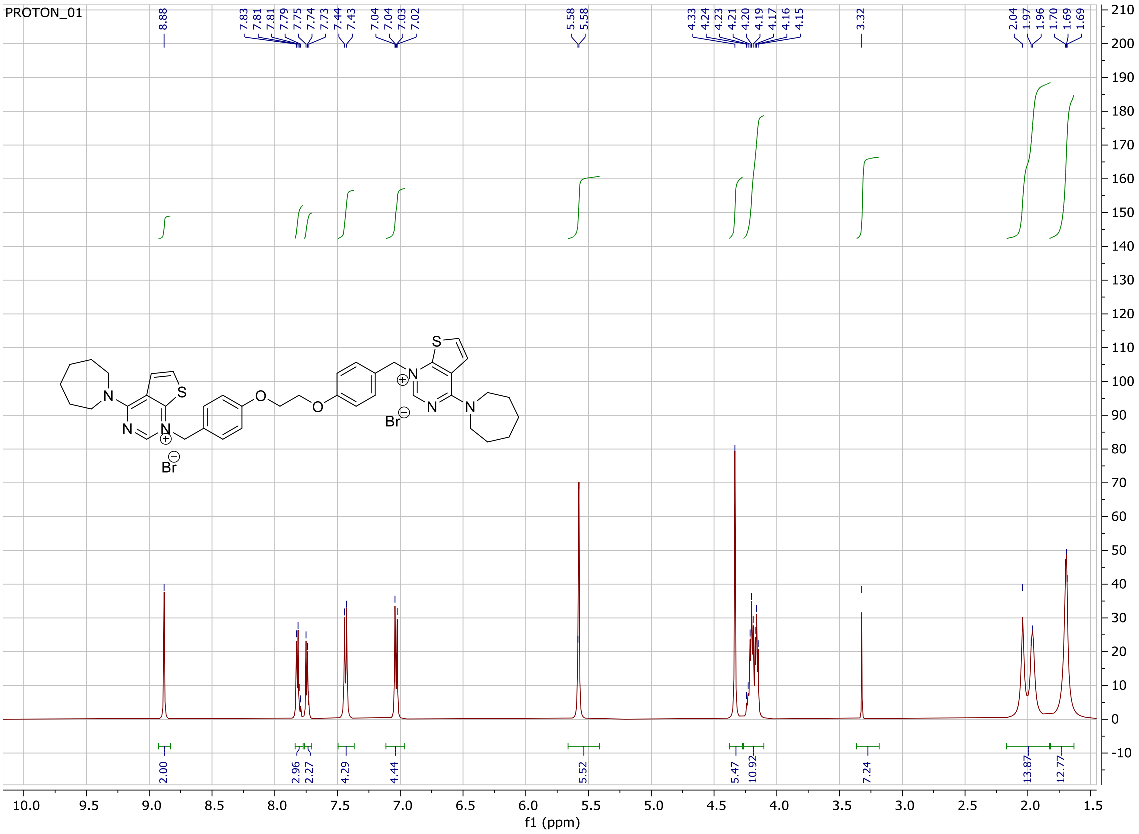

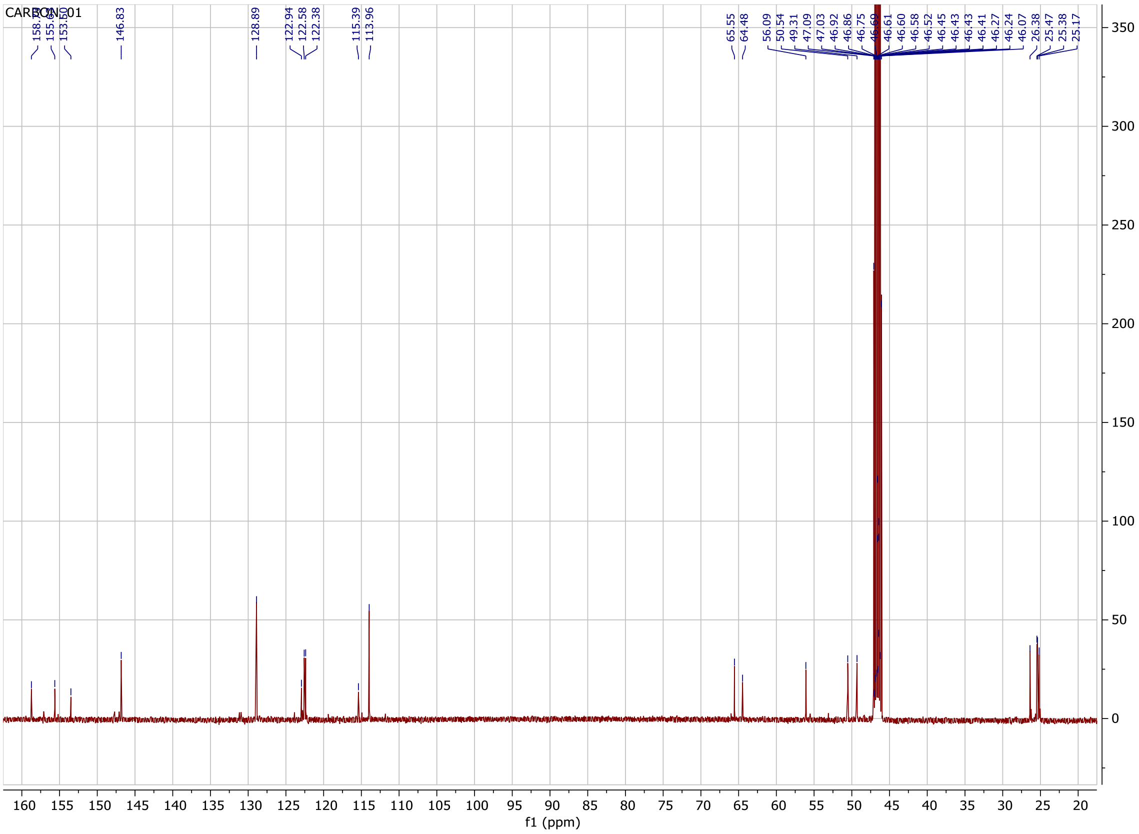

Figure S3. Spectra.
